# Raistrickiones A−E from a Highly Productive Strain of *Penicillium raistrickii* Generated through Thermo Change

**DOI:** 10.3390/md16060213

**Published:** 2018-06-18

**Authors:** De-Sheng Liu, Xian-Guo Rong, Hui-Hui Kang, Li-Ying Ma, Mark T. Hamann, Wei-Zhong Liu

**Affiliations:** 1 College of Pharmacy, Binzhou Medical University, Yantai 264003, China; desheng_liu@sina.com (D.-S.L.); binyirongxianguo@163.com (X.-G.R.); kanghuihui_1993@126.com (H.-H.K.); maliyingbz@163.com (L.-Y.M.); 2 Department of Drug Discovery and Biomedical Sciences, Medical University of South Carolina, Charleston, SC 29425, USA; hamannm@musc.edu

**Keywords:** *Penicillium raistrickii*, polyketides, diastereomers, thermo-change strategy

## Abstract

Three new diastereomers of polyketides (PKs), raistrickiones A−C (**1**–**3**), together with two new analogues, raistrickiones D and E (**4** and **5**), were isolated from a highly productive strain of *Penicillium raistrickii*, which was subjected to an experimental thermo-change strategy to tap its potential of producing new secondary metabolites. Metabolites **1** and **2** existed in a diastereomeric mixture in the crystal packing according to the X-ray data, and were laboriously separated by semi-preparative HPLC on a chiral column. The structures of **1**–**5** were determined on the basis of the detailed analyses of the spectroscopic data (UV, IR, HRESIMS, 1D, and 2D NMR), single-crystal X-ray diffractions, and comparison of the experimental and calculated electronic circular dichroism spectra. Compounds **1**–**5** represented the first case of 3,5-dihydroxy-4-methylbenzoyl derivatives of natural products. Compounds **1**–**5** exhibited moderate radical scavenging activities against 1,1-diphenyl-2-picrylhydrazyl radical 2,2-diphenyl-1-(2,4,6-trinitrophenyl) hydrazyl (DPPH).

## 1. Introduction

A plethora of publications have already delineated fungal strains from unique environments, including saline soil near the ocean, marine biospheres, and other special niches. Fungi from these locations have been shown to be an excellent biosynthetic source of chemical diversity and secondary metabolites (SMs) for pharmaceutical applications [1,2,3,4,5]. With the significant advancements in genomics and metagenomics, it has been clearly shown that the capacity for fungal strains to produce small molecules is determined by various biosynthetic gene clusters (BGCs) of their genome [6,7]. However, under routine or constant laboratory culture conditions, large amounts of BGCs remained “silent” and unexpressed [8], leading to the limited categories or numbers of SMs being produced. More recently, it was progressively elucidated that the silent BGCs could be successfully activated by manipulation of the culture conditions, such as cultivation-based and molecular approaches [9,10,11,12]. Among the cultivation-based approaches, thermo change is one of the effective strategies for triggering silent biosynthetic expression systems to enlarge the numbers of fungi-derived natural products, with only a few cases reported [13,14,15]. Strain JH-18 of *P. raistrickii*, which was isolated from marine saline soil of the coast of Bohai Bay in China, was investigated with only a few reports [16,17,18,19]. Its routine laboratory fermentation enabled the isolation of series of novel natural products including spiroketals, isocoumarins, α-pyrone, and dihydropyran derivatives [16,17,18], and some of them possess an unusual chemical skeleton and exhibit cytotoxic activity [20].

**Figure 1:**
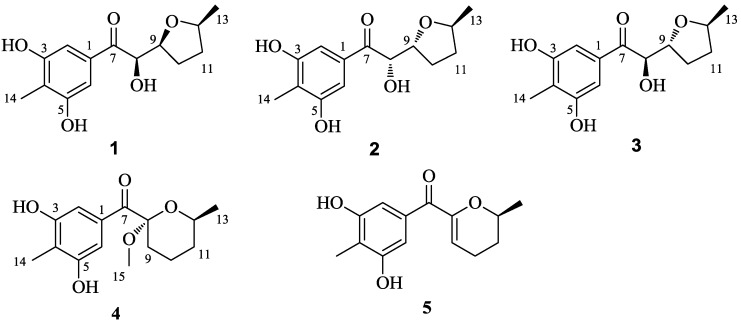
Structures of compounds **1**–**5**.

In view of the foregoing findings, a series of OSMAC (one strain–many compounds) [21] protocols were performed to investigate the possibility of unlocking the silent genes of this prolific genus to generate more new SMs. During the investigation, it was discovered that the thermo-change approach worked very well in evoking the synthetic expression systems. Five new polyketide (PKs), named raistrickiones A−E (**1**–**5**), which showed structural differences with those reported previously, were isolated in response to the fermentation temperature setting at 15 °C instead of 28 °C, with other conditions unchanged. In the current work, the isolation, purification, structure, elucidation, and biological evaluation of compounds **1**–**5** (Figure 1) were carried out.

## 2. Results

**Figure 2:**
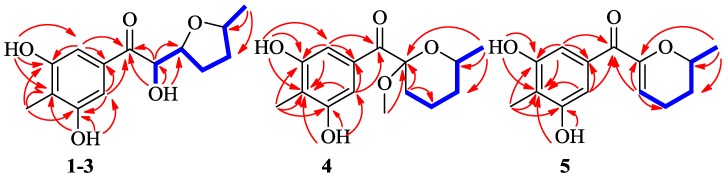
Key HMBC (red →) and ^1^H−^1^H COSY (blue ―) correlations of **1**–**5**.

**Figure 3:**
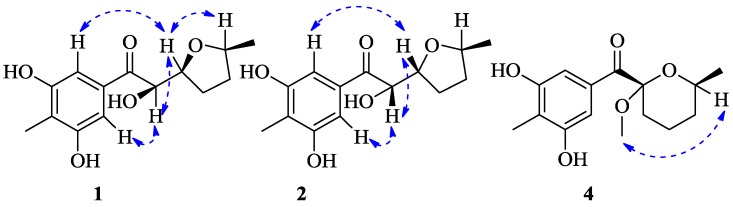
Key NOESY correlations (dashed blue arrows) of **1**, **2** and **4**.

**Figure 4:**
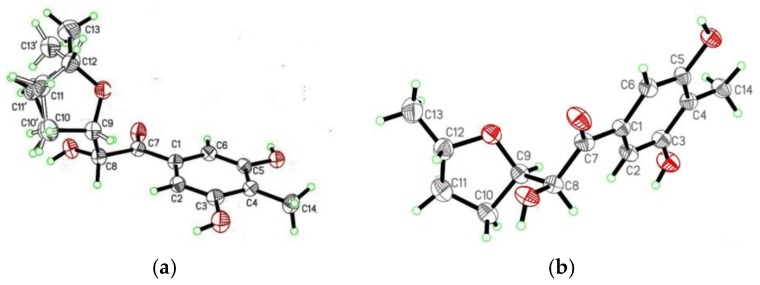
X-ray ORTEP drawings of **1** (**a**) and **2** (**b**).

**Figure 5:**
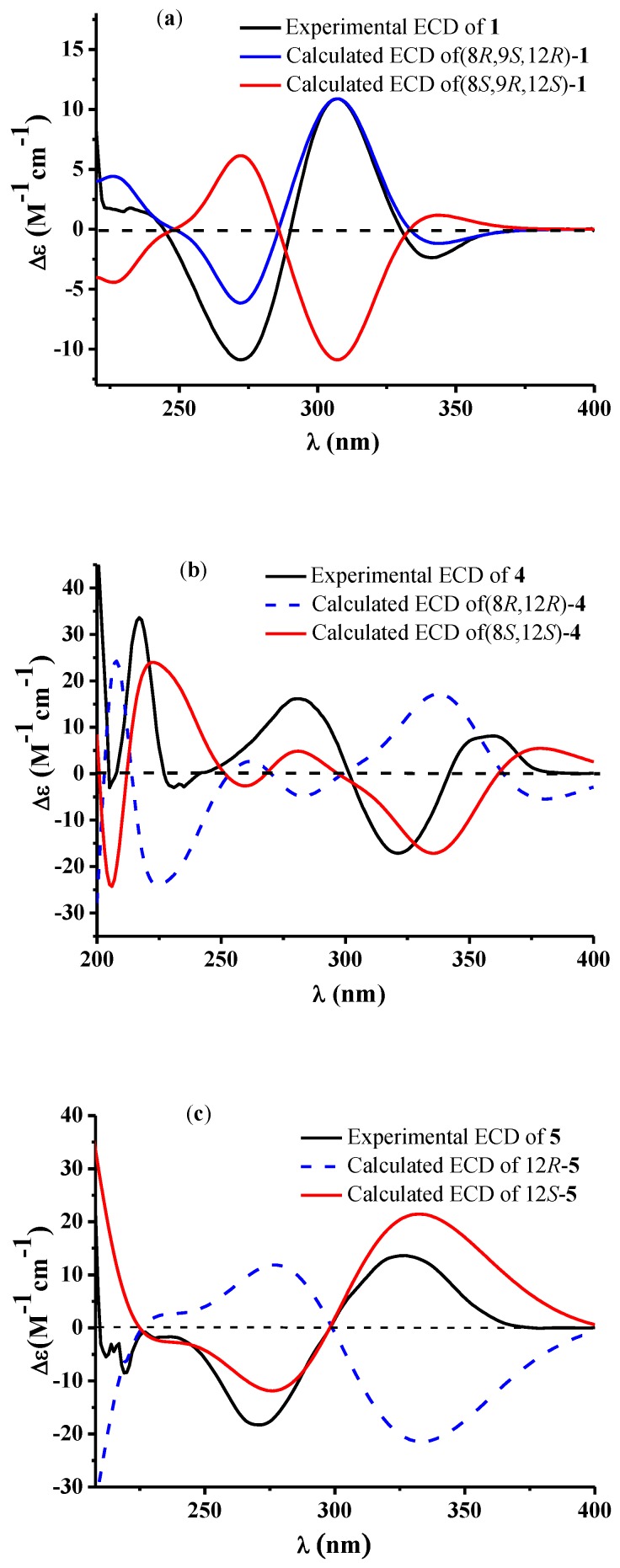
Experimental and calculated ECD of **1** (**a**), **4** (**b**) and **5** (**c**).

Raistrickione A (**1**) was separated as colorless plates in MeOH, and its molecular formula was determined as C_14_H_18_O_5_, with six indices of hydrogen deficiency, by the deprotonated molecular ion peak at *m/z* 265.1083 [M − H]^−^ (calculated for C_14_H_17_O_5_, 265.1081). The IR spectrum exhibited absorption bands for the presence of hydroxy (3259 cm^−1^, broad), keto carbonylic (1677 cm^−1^), and aromatic (1592 and 1421 cm^−1^) functionalities. The ^1^H NMR data (Table 1) in DMSO-*d*_6_ indicated two overlapped aromatic protons (δ_H_ 6.90, 2H, s), three hydroxy groups (two phenol and one alcoholic at δ_H_ 9.51, 2H, s; 4.84, 1H, d, *J* = 7.3 Hz, respectively), three oxygenated methines (δ_H_ 3.77, 1H, m; 4.13, 1H, m; 4.74, 1H, dd, *J* = 7.3, 3.5 Hz), two methylenes (δ_H_ 1.34, 1H, m; 1.84, 3H, m), and two methyls (δ_H_ 1.06, 3H, d, *J* = 6.0 Hz; 1.99, 3H, s). Analyses of the ^13^C NMR (DMSO-*d*_6_, Table 1) and DEPT data (Figure S17) demonstrated 14 carbon resonances, including two methyls, two methylenes, three oxygenated methines, one keto carbonyl, and six aromatic carbons (two tertiary and two oxygenated quaternary overlapped, respectively). The above information suggested a symmetrically tetrasubstituted phenyl ring in **1**. The upfield chemical shift (δ_C_ 8.9) of the arylmethyl group implied that it was sandwiched between two phenolic hydroxyls in the phenyl ring [16,17]. Furthermore, the upfield chemical shift (δ_C_ 199.2) of keto carbonyl suggested it to be conjugated with the phenyl ring, and located at the *para* position of the arylmethyl, which was proven by the non-chelated phenolic hydroxyl signals at δ_H_ 6.89. The presence of a 3,5-dihydroxyl-4-methylbenzoyl moiety was further disclosed by the associated HMBC correlations (Figure 2). The proton–proton correlation spectroscopy (^1^H-^1^H COSY, Figure 2) revealed a carbon chain from C-8 to C-13 (Figure 2), CHCHCH_2_CH_2_CHCH_3_, corresponding to all of the other carbons. Considering the ^1^H-^1^H COSY correlation of the alcoholic hydroxyl (δ_H_ 4.84) with H-8, C-8 connected with the hydroxyl, and the other two oxymethines at δ_C_ 74.9 (C-9) and 75.2 (C-12) should be linked through an oxygen atom to form a substituted tetrahydrofuran ring to meet the remaining index of hydrogen deficiency. The two moieties, a 3,5-dihydroxyl-4-methylbenzoyl unit and a disubstituted tetrahydrofuran ring, were linked together by the HMBC cross-peaks from the alcoholic hydroxyl to C-7, C-8, and C-9, and from H-8 (δ_H_ 4.74) to C-7 and C-9, respectively. Based on these results, the 2D structure of **1** was established and in agreement with all of the HSQC and HMBC data. In the NOESY spectrum of **1** (Figure 3), the correlations between H-9 and H-12 indicated that the two hydrogens occupied the same face. Finally, the absolute configuration of **1** was fully established as 8*R*, 9*S*, and 12*S* by the X-ray crystallography results, in which the tetrahydrofuran ring has two conformations in view of the disorder from C-10 to C-13 (Figure 4). The absolute configuration of **1** was further confirmed by the experimental and calculated electronic circular dichroism (ECD) data (Figure 5).

Raistrickione B (**2**) was also obtained as colorless plates. The negative HRESIMS data indicated that **2** has the same molecular formula of C_14_H_18_O_5_ as that of **1**. Its ^1^H and ^13^C NMR spectra (Table 1) highly resembled those of **1** with slight differences in the substituted tetrahydrofuran moiety, which further proved that **2** is a diasteremomer of **1** by 2D NMR, data as described above. The electronic circular dichroism (ECD) data (Figure S11) of **2** exhibited almost opposite-sign bands all through the spectrum in comparison with that of **1** (Figure S4). The cotton effects in the ECD spectra suggested the two compounds had an opposite configuration at C-8, and hence an 8*S* absolute configuration in **2**. The configuration of the chiral center of C-9 was assigned by comparison of the vicinal proton–proton coupling constants between H-8 and H-9 in compounds **1** and **2**, which exhibited almost an identical magnitude (^3^*J*_HH_ = 3.5 and 3.3 Hz for **1** and **2**, respectively). According to the Karplus equation [22], the dihedral angle between H-8 and H-9 in compounds **1** and **2** should possess the same geometric behavior in their relative spatial arrangement, which consequentially led to the identification of the 9*R* absolute configuration in **2**. Based on the X-ray crystallography (Figure 4) data, the absolute configuration of **2** was fully established as 8*S*, 9*R*, and 12*S*. This was coincident with the optical rotation values (+47.5 and −48.6 for **1** and **2** in MeOH, respectively), which intensified the validity of the absolute configuration of **2**.

Raistrickione C (**3**) was afforded as colorless powder. The HRESIMS data assigned the same molecular formula as those of **1** and **2**. The ^1^H and ^13^C NMR spectra of **3** (Table 1) were closely similar to those of **2**. All of the information illustrated that it was another diastereoisomer that was different from **1** and **2**. The identical ECD data of **1** and **3** were reminiscent of the same configurational behavior, and hence an 8*R* absolute configuration in **3**. The t multiplicity (dd in **2**) of H-8 and the larger *J* value (^3^*J*_HH_ = 5.8 Hz) between H-8 and H-9 (^3^*J*_HH_ = 3.3 Hz in **2**) in **3** were indicative of the absolute configuration of C-9 to be *R*. In the NOESY spectrum (Figure S31), no cross-peak was observed between H-9 and H-12, so the H-9 and H-12 should be in *trans* orientations. The configuration of C-12 should be the same as in **2**, which was supported by the high similarity of ^1^H and ^13^C NMR data (Table 1) among C-11, C-12, and C-13 within compounds **2** and **3**. Consequently, the absolute configuration of **3** was established as 8*R*, 9*R*, and 12*S*.

Raistrickione D (**4**) was isolated as colorless needles in MeOH. The molecular formula of **4** was determined on the basis of the NMR data and the HRESIMS results, which showed a deprotonated molecular ion peak [M − H]^−^ at *m*/*z* 279.1236 (calculated for C_15_H_19_O_5_, 279.1238) with 14 *amu* more than those of compounds **1**–**3**, accounting for six indices of hydrogen deficiency. In the IR spectrum of **4,** absorptions at 3337 (broad) cm^−1^, 1668 cm^−1^, and 1588 cm^−1^, were assigned to hydroxy, carbonylic, and aromatic functionalities, respectively. The UV spectrum (Figure S34) exhibited similar absorptions with those of **1**–**3**, indicating that **4** was an analogue of those compounds. The ^1^H and ^13^C NMR spectra (Table 2) presented a keto carbonyl (δ_C_ 196.5), two overlapped oxygenated aromatic quaternary carbons (δ_C_ 156.8), two aromatic quaternary carbons (δ_C_ 133.9, 117.6), two overlapped aromatic methines (δ_C_ 109.2; δ_H_ 7.36, 2H, s), an aromatic methyl (δ_C_ 9.0; δ_H_ 2.13, 3H, s), and two phenol hydroxyls (δ_H_ 8.43, 2H, s), which suggested there is a 3,5-dihydroxyl-4-methylbenzoyl moiety in **4** as in **1**. In addition, one dioxygenated quaternary carbon, one methoxyl, one methyl, three methylenes, and one methine were observed in the NMR spectra of **4**. The COSY system (Figure 2) presented a proton spin system from H-9 to H-13, which anchored at the keto carbonyl (C-7) through the dioxygenated carbon (C-8) according to the HMBC correlations (Figure 2) from H-9 to C-7 and C-8. The HMBC correlation from the methoxyl (H-15) to the dioxygenated carbon (C-8) established its placement. The remaining degree of unsaturation completed the tetrahydropyran ring. The NOESY spectrum (Figure 3) of **4** exhibited cross-peaks between H-15 and H-12, indicating that the methoxyl and H-12 were on the same face of the tetrahydropyran ring, which determined the relative configuration of C-8 and C-12. The absolute configuration of **4** was solved by comparison of its experimental ECD spectrum with the predicted one by time-dependent density functional theory (TDDFT) calculation at the B3LYP/6-311G (d, p) level. As a result, the calculated ECD curve of (8*S*, 12*S*)-**4** (Figure 5) was in line with the experimental one. Therefore, the absolute configuration of **4** was elucidated as 8*S*, 12*S*.

Raistrickione E (**5**) was afforded as colorless powder. Its molecular formula C_14_H_16_O_4_ was given based on the HREISMS data, which exhibited a deprotonated molecular ion peak [M − H]^−^ at *m*/*z* 247.0976 (calculated for C_14_H_15_O_4_, 247.0976), with seven indices of hydrogen deficiency. The ^1^H and ^13^C NMR data (Table 2) in acetone-*d_6_* were similar to those of **4**, except for the absence of a methoxyl, a dioxygenated quarternary carbon, and a methylene which existed in **4**, and the appearance of an oxygenated trisubstituted double bond (δ_C_ 152.7, 112.2; δ_H_ 5.71, 1H, t, *J* = 3.8 Hz) in **5**. This implied that there was a double bond between C-8 and C-9, which was further confirmed by the COSY spin system from H-9 to H-13, along with the HMBC correlations of H-9 with C-7, C-8, and C-10, and H-13 with C-8, C-11, and C-12 (Figure 2). Therefore, the two-dimensional (2D) structure of **5** was established. Similarly, the stereochemistry of **5** was assigned as 12*S* by comparing its experimental and theoretical ECD data (Figure 5).

Compounds **1**–**5** exhibited moderate radical scavenging activities against DPPH with IC_50_ values of 32 ± 2.5 μM, 38 ± 1.9 μM, 40 ± 3.6 μM, 49 ± 2.1 μM, and 42 ± 1.2 μM, respectively; ascorbic acid was applied as a positive control (IC_50_: 17 ± 1.7 μM). They were further evaluated for their cytotoxic effect against human leukemia (HL60) cell lines by the MTT method, and all of them were inactive (>20 μM).

## 3. Discussion

**Figure 6:**
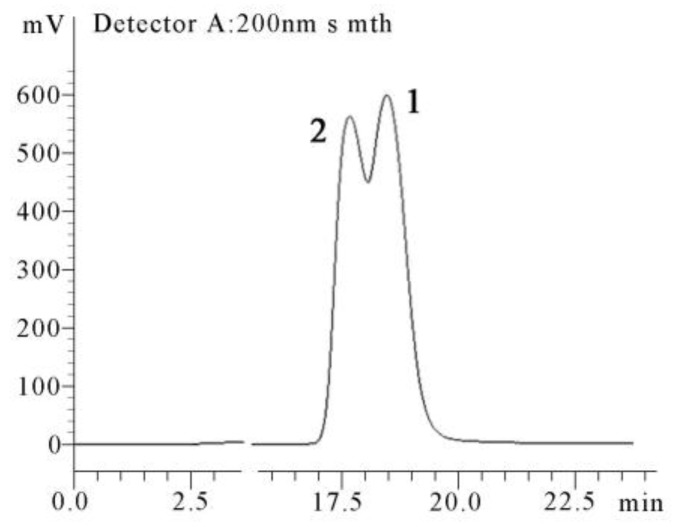
Separation of **1** and **2** on a chiral HPLC column.

Intriguingly, a mixture of **1** and **2** was superficially obtained as a pure chemical at first and displayed characteristics of a dimer of compound **3** in NMR spectroscopic data, which was contradicted by the mass spectrometric results. Those observations suggested that it was a partial racemate in nature. This assumption was subsequently verified by the single-crystal X-ray diffraction analysis (Figure 4). Then, a preparative chiral HPLC was applied to separate **1** and **2** in an extremely time-consuming and repeated purification process, with a ratio of ca. 1:1 (Figure 6). The former peak centered at 17.72 min (retention time) was identified as **2**, and the latter one centered at 18.53 min was identified as **1**. Detailed comparison of the spectroscopic data revealed that the ^1^H and ^13^C NMR spectra of the isolated diastereoisomers assembled those of their precursor mixture (Figure S5, Figure S12, and Figure S15, ^1^H spectra for **1**, **2**, and the precursor mixture, respectively; Figure S6, Figure S13, and Figure S16, ^13^C spectra for **1**, **2**, and the precursor mixture, respectively). Unlike enantiomers, most diastereomers are easy to be separated by reverse phase HPLC or normal preparative thin layer chromatography (TLC) [23,24,25,26,27,28], due to their different physical properties. Only a few diastereomers need to use chiral HPLC to isolate [29,30].

## 4. Materials and Methods

### 4.1. General Experimental Procedures

Melting points were determined with an XRC-1 micro-melting point apparatus (Sichuan University Scientific Instrument Factory, Chengdu, China) and were uncorrected. Optical rotations were measured on an Autopol V Plus digital polarimeter (Rudolph Research Analytical, Hackettstown, NJ, USA). UV spectra were obtained on a TU-1091 spectrophotometer (Beijing Purkinje General Instrument Co., Beijing, China). ECD spectra were recorded with a Chirascan spectropolarimeter (Applied Photophysics, Leatherhead, United Kingdom). IR spectra were carried out on a Nicolet 6700 spectrophotometer (Thermo Scientific, Waltham, MA, USA) by an attenuated total reflectance (ATR) approach. NMR data were obtained at 400 MHz and 100 MHz for ^1^H and ^13^C, respectively, on an Avance 400 (Bruker, Billerica, MS, USA) with TMS as the internal standard. Crystal structure determination was performed on a Bruker Smart 1000 CCD X-ray diffractometer (Bruker Biospin Group, Karlstuhe, Germany). HRESIMS was acquired on a 1200RRLC-6520 Accurate-Mass Q-TOF LC/MS mass spectrometer (Agilent, Santa Clara, CA, USA). Semi-preparative HPLC was accomplished on a LC-6AD Liquid Chromatography (Shimadzu, Kyoto, Japan) with an SPD-20A Detector by an ODS column (HyperClone 5 μm ODS (C_18_) 120 Å, 250 × 10 mm, Phenomenex, 4 mL/min.) Chiral HPLC was carried out on a column [ChiralPAK IC, 5 μm cellulose tri(3,5-dichlorophenyl carbamate), 250 × 10 mm, Daicel Chiral Technologies Co. LTD. (Shaihai, China), 4 mL/min]. Sephadex LH-20 (Ge Healthcare Bio-Sciences AB, Uppsala, Sweden), silica gel (200−300 mesh, Qingdao Marine Chemical Inc., Qingdao, China), and reversed-phase C_18_ silica gel (Pharmacia Fine Chemical Co., Ltd., Uppsala, Sweden) were used for open column chromatography.

### 4.2. Fungal Material

Strain JH-18 of *P. raistrickii* (Genbank accession No. HQ717799), was isolated from the marine saline soil as previously reported [18]. The strain is kept at College of Pharmacy, Binzhou Medical University.

### 4.3. Fermentation, Extraction, and Isolation

The fermentation and extraction procedures were almost the same as described in a previous article [18], except for the fermentation temperature setting at 15 °C instead of 28 °C. The whole culture broth (40 L) afforded 23 g of crude extract. The extract was subjected to a silica gel column, eluting with different solvents of increasing polarity from petroleum ether, chloroform to MeOH to yield eight fractions (Fr.s 1–8) based on TLC analysis. Fr. 4 (12 g) was passed through a reversed-phase column eluting with MeOH-water gradient (from 20:80 to 100:0) to afford nine subfractions (Fr.s 4.1–4.9). Fr. 4.3 (2.8 g) was further separated on a Sephadex LH-20 column eluting with MeOH to afford five subfractions (Fr.s 4.3.1–4.3.5). Fr. 4.3.2 (98.1 mg) was purified by semipreparative HPLC on an ODS column eluting with MeOH-0.2% trifluoroacetic acid (TFA) aqueous solution (*v/v*) (4:6, 4 mL/min) to yield the diastereomeric mixture (29.2 mg, *t*_R_ 12.5 min) of **1** and **2**. Then, the mixture was separated on semi-preparative HPLC using the chiral column (hexane-isopropanol, 9:1; 4 mL/min) to yield compounds **1** (12.3 mg, *t*_R_ 18.53 min) and **2** (10.0 mg, *t*_R_ 17.72 min). Fr. 4.3.4 (68.0 mg) was reloaded on semi-preparative HPLC using an ODS column eluting with MeOH-0.2% TFA aqueous solution (5:5; 4 mL/min) to afford compound **3** (12.0 mg, *t*_R_ 23.5 min). Fr. 4.4 (1.1 g) was passed through a Sephadex LH-20 column to yield subfractions (Fr.s 4.4.1–4.4.8) eluting with MeOH. Purification of Fr. 4.4.5 (30.1 mg) by semi-preparative HPLC on an ODS column (MeOH-0.2% TFA aqueous solution; 4 mL/min) afforded compound **4** (9.0 mg, *t*_R_ 13.6 min). Fr. 4.6 (0.9 g) was further chromatographed on CC of Sephadex LH-20, developed by MeOH and then purified by semipreparative HPLC on an ODS column (MeOH-0.2% TFA aqueous solution, 5:5; 4 mL/min) to yield compound **5** (11.0 mg, *t*_R_ 9.1 min).

Raistrickione A (**1**): colorless plates (MeOH); mp 198–199 °C; ${\left[\alpha \right]}_{D}^{25}$ +47.5 (*c* 0.064, MeOH); UV (MeOH) *λ*_max_ (log ε) 220 (4.20), 279 (3.86) nm; IR (ATR) v_max_ 3259 (broad), 1677, 1592, 1421, 1335, 1192, 1086, 800, 721 cm^−1^; ECD (MeOH) *λ*_max_ (Δε) 341 (−2.37), 307 (+10.88), 272 (−10.88), 232 (+1.78) nm; ^1^H and ^13^C NMR data, see Table 1; HRESIMS *m/z* 265.1083 [M − H]^−^ (calculated for C_14_H_17_O_5_, 265.1081).

Raistrickione B (**2**): colorless plates (MeOH); mp 174–176 °C; ${\left[\alpha \right]}_{D}^{25}$−48.6 (*c* 0.050, MeOH); UV (MeOH) *λ*_max_ (log ε) 220 (4.15), 280 (3.81) nm; IR (ATR) v_max_ 3224 (broad), 1678, 1596, 1423, 1336, 1094, 879, 798 cm^−1^; ECD (MeOH) *λ*_max_ (Δε) 342 (+2.62), 308 (−13.01), 273 (+14.20), 236 (−3.23) nm; ^1^H and ^13^C NMR data, see Table 1; HRESIMS *m/z* 265.1082 [M − H]^−^ (calculated for C_14_H_17_O_5_, 265.1081).

Raistrickione C (**3**): colorless powder; ${\left[\alpha \right]}_{D}^{25}$+3.3 (*c* 0.058, MeOH); UV (MeOH) *λ*_max_ (log ε) 219 (4.21), 282 (3.89) nm; IR (ATR) v_max_ 3233 (broad), 1669, 1592, 1420, 1336, 1092, 998, 868, 800 cm^−1^; ECD (MeOH) *λ*_max_ (Δε) 342 (−2.86), 310 (+5.53), 276 (−7.55), 240 (+1.86) nm; ^1^H and ^13^C NMR data, see Table 1; HRESIMS *m/z* 267.1226 [M + H]^+^ (calculated for C_14_H_19_O_5_, 267.1227).

Raistrickione D (**4**): colorless needles (MeOH); mp 154–156 °C; ${\left[\alpha \right]}_{D}^{25}$−66.8 (*c* 0.106, MeOH); UV (MeOH) *λ*_max_ (log ε) 220 (4.17), 286 (3.92) nm; IR (ATR) v_max_ 3337 (broad), 1668, 1588, 1417, 1333, 1080, 889, 788, 714 cm^−1^; ECD (MeOH) *λ*_max_ (Δε) 359 (+8.13), 321 (−17.14), 280 (+16.13) nm; ^1^H and ^13^C NMR data, see Table 2; HRESIMS *m/z* 279.1236 [M − H]^−^ (calculated for C_15_H_19_O_5_, 279.1238).

Raistrickione E (**5**): colorless needles; mp 180–182 °C; ${\left[\alpha \right]}_{D}^{25}$-28.7 (*c* 0.080, MeOH); UV (MeOH) *λ*_max_ (log ε) 207 (4.32), 287 (3.97) nm; IR (ATR) v_max_ 3315 (broad), 3137 (broad), 1617, 1577, 1415, 1336, 1213, 1070, 891, 875, 755 cm^−1^; ECD (MeOH) *λ*_max_ (Δε) 327 (+13.62), 271 (−18.28), 227 (−0.78) nm; ^1^H and ^13^C NMR data, see Table 2; HRESIMS *m/z* 247.0976 [M − H]^−^ (calculated for C_14_H_15_O_4_, 247.0976).

### 4.4. X-ray Crystallographic Analysis of the Diastereomeric Mixture of **1** and **2**

C_14_H_18_O_5_, *M* = 266.28, Monoclinic, space group *P*2(1); Unit cell dimensions were determined to be *a* = 11.7531(9) Å, *b* = 9.9251(6) Å, *c* = 11.7968(10) Å, *α* = 90°, *β* = 105.118 (2)°, *γ* = 90°, *V* = 1328.48 (17) Å^3^, *Z* = 4, *D*_calculated_ = 1.331 mg/m^3^, F(000) = 568, Crystal size 0.30 × 0.21 × 0.13 mm, *μ*(Cu Kα) = 0.840 mm^−1^. Single crystals were measured on a Bruker Smart 1000 CCD X-ray diffractometer equipped with graphite-monochromated Cu K*α* radiation (*λ =* 1.54178 Å) at 293 (2) K. A total of 7896 reflections were collected until *θ*_max_ = 66.04°, in which 4137 independent unique reflections were observed (*R*_int_ = 0.0712). The structure was solved by direct methods with the SHELXTL software package, and refined by full-matrix least-squares on *F*^2^. The final refinement gave *R*_1_ = 0.0628 and *wR*_2_ = 0.1457 (I > 2*σ*(*I*)). The crystallographic data for the structures of the diastereomeric mixture of **1** and **2** have been deposited in the Cambridge Crystallographic Data Centre (deposition number: CCDC 1839882).

### 4.5. Antioxidant Activity Assay

In the DPPH scavenging assay, the tested samples were dissolved in MeOH at the concentrations of 200 μM, 100 μM, 50 μM, 25 μM, and 12.5 μM. Then, 160 μL of the sample solutions was dispensed into the wells of a 96-well microtiter plate, and 40 μL of DPPH solutions in MeOH (400 μM, 200 μM, 100 μM, 50 μM, and 25 μM) was added to each well. The mixture was shaken vigorously and kept in the dark for 30 min. Then, the absorbance was measured at 517 nm using methanol as the blank reference. All of the experiments were performed in triplicate [31].

### 4.6. Cytotoxicity Assay

The cytotoxicity assay against human leukemia (HL60) cell lines was performed in triplicate using our previously described method, with doxorubicin as positive control (IC_50_ value of 1.56 ± 0.32 μM). Five final concentrations (from 100 μM to 1 μM) in DMSO of the tested compound solutions were set in the wells of 96-well microtiter plates [31].

## 5. Conclusions

A thermo-change strategy applied to the prolific strain JH-18 of *P. raistrickii* afforded five new antioxidant PKs: raistrickiones A−E (**1**–**5**). At first, compounds **1** and **2** were obtained as a diastereomeric mixture, and their absolute configurations were determined in a crystal by X-ray diffraction analysis. Then, they were arduously separated by semi-preparative HPLC on a chiral column. Compounds **1**–**3** were diastereomeric at the C-8 and C-9 centers, but no distinct differences were observed in light of the antioxidant and cytotoxic results. In addition, compounds **1**–**5** showed considerable difference in skeleton with those reported previously from this fungal strain. Our work provides further demonstration that environmental cues such as huge thermo change represent a powerful approach in unlocking silent BCGs to produce new chemical compounds from fungi. The additional applications of other OSMAC strategies are currently underway, which could determine future reports on the discovery of new molecules from *P. raistrickii*.

## Figures and Tables

**Table 1:** NMR Spectroscopic Data (^1^H 400 MHz and ^13^C 100 MHz) of **1**–**3** (DMSO-*d*_6_).

Position	1		2		3	
	δ_C_	δ_H_ (*J* in Hz)	δ_C_	δ_H_ (*J* in Hz)	δ_C_	δ_H_ (*J* in Hz)
1	133.0, C		132.9, C		133.5, C	
2, 6	106.2, CH	6.90, s	106.2, CH	6.89, s	106.2, CH	6.94, s
3, 5	156.2, C		156.2, C		156.3, C	
4	116.6, C		116.6, C		116.7, C	
7	199.0, C		199.2, C		199.4, C	
8	74.9, CH	4.74, dd (7.3, 3.5)	75.4, CH	4.69, dd (7.3, 3.3)	74.8, CH	4.73, t (5.8)
9	80.1, CH	4.13, m	79.5, CH	4.27, td (7.0, 3.3)	79.3, CH	4.20, q (5.8)
10	27.3, CH_2_	1.84, ^a^ m	27.8, CH_2_	1.93, ^a^ m	26.5, CH_2_	1.84, m; 1.77, m
11	32.5, CH_2_	1.84, ^a^ m; 1.34, m	33.4, CH_2_	1.93, ^a^ m; 1.31, m	33.1, CH_2_	1.99, m; 1.34, m
12	75.2, CH	3.77, m	75.4, CH	3.99, m	75.0, CH	4.04, m
13	20.6, CH_3_	1.06, d (6.0)	21.0, CH_3_	1.02, d (6.1)	21.0, CH_3_	1.04, d (6.0)
14	8.9, CH_3_	1.99, s	8.9, CH_3_	1.99, s	8.9, CH_3_	1.99, s
15						
OH-3, 5		9.51, s		9.52, s		9.49, s
OH-8		4.84, d (7.3)		4.96, d (7.3)		5.31, d (5.8)

^a^ Overlapping signals.

**Table 2:** NMR Spectroscopic Data (^1^H 400 MHz and ^13^C 100 MHz) of **4** and **5**.

Position	4 *^a^*		5 *^a^*	
	δ_C_	δ_H_ (*J* in Hz)	δ_C_	δ_H_ (*J* in Hz)
1	133.9, C		136.3, C	
2, 6	109.2, CH	7.36, s	108.7, CH	6.94, s
3, 5	156.8, C		156.8, C	
4	117.6, C		116.8, C	
7	196.5, C		190.1, C	
8	102.6, C		152.7, C	
9	32.3, CH_2_	1.80, m; 1.65, *^b^* m	112.2, CH	5.71, t (3.8)
10	19.7, CH_2_	1.91, m; 1.65, *^b^* m	21.6, CH_2_	2.30, m; 2.20, m
11	32.7, CH_2_	1.65, *^b^* m; 1.40, m	29.3, CH_2_	1.94, m; 1.55, m
12	68.0, CH	3.88, m	73.0, CH	4.04, m
13	22.0, CH_3_	1.22, d (6.3)	21.2, CH_3_	1.32, d (6.2)
14	9.0, CH_3_	2.13, s	9.0, CH_3_	2.15, s
15	50.5, CH_3_	3.17, s		
OH-3, 5		8.43, s		8.45, s
OH-8				

*^a^* NMR spectra obtained in acetone-*d*_6_. *^b^* Overlapping signals.

## Supplementary Materials

The following are available online at http://www.mdpi.com/1660-3397/16/6/213/s1, Figure S1. HRESIMS of raistrickione A (**1**), Figure S2: IR spectrum (ATR approach) of raistrickione A (**1**), Figure S3: UV spectrum (MeOH) of raistrickione A (**1**), Figure S4: ECD spectrum (MeOH) of raistrickione A (**1**), Figure S5: ^1^H NMR spectrum (400 MHz DMSO-*d*_6_) of raistrickione A (**1**), Figure S6: ^13^C NMR spectrum (100 MHz DMSO-*d*_6_) of raistrickione A (**1**), Figure S7: NOSEY spectrum (DMSO-*d*_6_) of raistrickione A (**1**), Figure S8: HRESIMS of raistrickione B (**2**), Figure S9: IR spectrum (ATR approach) of raistrickione B (**2**), Figure S10:. UV spectrum (MeOH) of raistrickione B (**2**), Figure S11: ECD spectrum (MeOH) of raistrickione B (**2**), Figure S12: ^1^H NMR spectrum (400 MHz DMSO-*d*_6_) of raistrickione B (**2**), Figure S13:. ^13^C NMR spectrum (100 MHz DMSO-*d*_6_) of raistrickione B (**2**), Figure S14:. NOSEY spectrum (DMSO-*d*_6_) of raistrickione B (**2**), Figure S15: ^1^H NMR spectrum (400 MHz DMSO-*d*_6_) of the diastereoisomeric mixture (**1** and **2**), Figure S16: ^13^C NMR spectrum (100 MHz DMSO-*d*_6_) of the diastereoisomeric mixture (**1** and **2**), Figure S17: DEPT of the diastereoisomeric mixture (**1** and **2**), Figure S18:. COSY of the diastereoisomeric mixture (**1** and **2**), Figure S19: HSQC of the diastereoisomeric mixture (**1** and **2**), Figure S20:. HMBC of the diastereoisomeric mixture (**1** and **2**), Figure S21: HRESIMS of raistrickione C (**3**), Figure S22:. IR of raistrickione C (**3**), Figure S23: UV spectrum (MeOH) of raistrickione C (**3**), Figure S24: ECD spectrum (MeOH) of raistrickione C (**3**), Figure S25: ^1^H NMR spectrum (400 MHz DMSO-*d*_6_) of raistrickione C (**3**), Figure S26: ^13^C NMR spectrum (100 MHz DMSO-*d*_6_) of raistrickione C (**3**), Figure S27: DEPT of raistrickione C (**3**), Figure S28: COSY of raistrickione C (**3**), Figure S29: HSQC of raistrickione C (**3**), Figure S30: HMBC of raistrickione C (**3**), Figure S31: NOESY of raistrickione C (**3**), Figure S32: HRESIMS of raistrickione D (**4**), Figure S33: IR of raistrickione D (**4**), Figure S34: UV spectrum (MeOH) of raistrickione D (**4**), Figure S35: ^1^H NMR spectrum (400 MHz acetone-*d*_6_) of raistrickione D (**4**), Figure S36: ^13^C NMR spectrum (100 MHz acetone-*d*_6_) of raistrickione D (**4**), Figure S37: DEPT of raistrickione D (**4**), Figure S38: COSY of raistrickione D (**4**), Figure S39: HSQC of raistrickione D (**4**), Figure S40: HMBC of raistrickione D (**4**), Figure S41: NOSEY of raistrickione D (**4**), Figure S42: HRESIMS of raistrickione E (**5**), Figure S43: IR of raistrickione E (**5**), Figure S44: UV spectrum (MeOH) of raistrickione E (**5**), Figure S45: ^1^H NMR spectrum (400 MHz acetone-*d*_6_) of raistrickione E (**5**), Figure S46: ^13^C NMR spectrum (100 MHz acetone-*d*_6_) of raistrickione E (**5**), Figure S47: DEPT of raistrickione E (**5**), Figure S48: COSY of raistrickione E (**5**), Figure S49: HSQC of raistrickione E (**5**), Figure S50: HMBC of raistrickione E (**5**), and the computational parts.
