# Supplementary material for: Raistrickiones A−E from a Highly Productive Strain of Penicillium raistrickii Generated through Thermo Change
Source: Mar Drugs. 2018 Jun 18;16(6):213. doi: 10.3390/md16060213 (PMC6025261; doi:10.3390/md16060213)
Supplement: Supplementary file 1 [file marinedrugs-16-00213-s001.pdf]

SUPPORTING INFORMATION

Raistrickiones A−E from a highly productive strain of *Penicillium raistrickii* generated through thermo change

De-Sheng Liu [1], Xian-Guo Rong[1], Hui-Hui Kang[1] , Li-Ying Ma[1] , Mark T Hamann[2] and Wei-Zhong Liu[1,*]

[1] College of Pharmacy, Binzhou Medical University, Yantai, 264003, China; E-mail: desheng_liu@sina.com (D.-S.L.); binyirongxianguo@163.com (X.-G.R.); kanghuihui_1993@126.com (H.-H.K.); maliyingbz@163.com (L.-Y.M.)

[2] Department of Drug Discovery and Biomedical Sciences, Medical University of South Carolina, Charleston, South Carolina 29425, United States; hamannm@musc.edu

* Correspondence: lwz1963@163.com; Tel.: +86-535-691-3205

List of supporting information

Figure S1. HRESIMS of raistrickione A (**1**) 4
Figure S2. IR spectrum (ATR approach) of raistrickione A (**1**) 4
Figure S3. UV spectrum (MeOH) of raistrickione A (**1**) 5
Figure S4. ECD spectrum (MeOH) of raistrickione A (**1**) 5
Figure S5. ^{1}H NMR spectrum (400 MHz DMSO-d_6) of raistrickione A (**1**) 6
Figure S6. ^{13}C NMR spectrum (100 MHz DMSO-d_6) of raistrickione A (**1**) 6
Figure S7. NOSEY spectrum (DMSO-d_6) of raistrickione A (**1**) 7
Figure S8. HRESIMS of raistrickione B (**2**) 7
Figure S9. IR spectrum (ATR approach) of raistrickione B (**2**) 8
Figure S10. UV spectrum (MeOH) of raistrickione B (**2**) 8
Figure S11. ECD spectrum (MeOH) of raistrickione B (**2**) 9
Figure S12. ^{1}H NMR spectrum (400 MHz DMSO-d_6) of raistrickione B (**2**) 9
Figure S13. ^{13}C NMR spectrum (100 MHz DMSO-d_6) of raistrickione B (**2**) 10
Figure S14. NOSEY spectrum (DMSO-d_6) of raistrickione B (**2**) 10
Figure S15. ^{1}H NMR spectrum (400 MHz DMSO-d_6) of the diastereoisomeric mixture (**1** and **2**) 11
Figure S16. ^{13}C NMR spectrum (100 MHz DMSO-d_6) of the diastereoisomeric mixture (**1** and **2**) 11
Figure S17. DEPT of the diastereoisomeric mixture (**1** and **2**) 12
Figure S18. COSY of the diastereoisomeric mixture (**1** and **2**) 12
Figure S19. HSQC of the diastereoisomeric mixture (**1** and **2**) 13
Figure S20. HMBC of the diastereoisomeric mixture (**1** and **2**) 13
Figure S21. HRESIMS of raistrickione C (**3**) 14
Figure S22. IR spectrum (ATR approach) of raistrickione C (**3**) 14
Figure S23. UV spectrum (MeOH) of raistrickione C (**3**) 15
Figure S24. ECD spectrum (MeOH) of raistrickione C (**3**) 15
Figure S25. ^{1}H NMR spectrum (400 MHz DMSO-d_6) of raistrickione C (**3**) 16
Figure S26. ^{13}C NMR spectrum (100 MHz DMSO-d_6) of raistrickione C (**3**) 16
Figure S27. DEPT of raistrickione C (**3**) 17
Figure S28. COSY of raistrickione C (**3**) 17
Figure S29. HSQC of raistrickione C (**3**) 18
Figure S30. HMBC of raistrickione C (**3**) 18
Figure S31. NOESY of raistrickione C (**3**) 19
Figure S32. HRESIMS of raistrickione D (**4**) 19
Figure S33. IR spectrum (ATR approach) of raistrickione D (**4**) 20
Figure S34. UV spectrum (MeOH) of raistrickione D (**4**) 20
Figure S35. ^{1}H NMR spectrum (400 MHz acetone-d_6) of raistrickione D (**4**) 21
Figure S36. ^{13}C NMR spectrum (100 MHz acetone-d_6) of raistrickione D (**4**) 21
Figure S37. DEPT of raistrickione D (**4**) 22
Figure S38. COSY of raistrickione D (**4**) 22
Figure S39. HSQC of raistrickione D (**4**) 23
Figure S40. HMBC of raistrickione D (**4**) 23
Figure S41. NOSEY of raistrickione D (**4**) 24

Figure S42. HRESIMS of raistrickione E (**5**)..24
Figure S43. IR spectrum (ATR approach) of raistrickione E (**5**) 25
Figure S44. UV spectrum (MeOH) of raistrickione E (**5**)..25
Figure S45. ^{1}H NMR spectrum (400 MHz acetone-d_6) of raistrickione E (**5**)..26
Figure S46. ^{13}C NMR spectrum (100 MHz acetone-d_6) of raistrickione E (**5**)..26
Figure S47. DEPT of raistrickione E (**5**)...27
Figure S48. COSY of raistrickione E (**5**) 27
Figure S49. HSQC of raistrickione E (**5**) 28
Figure S50. HMBC of raistrickione E (**5**) 28
Computational parts 29

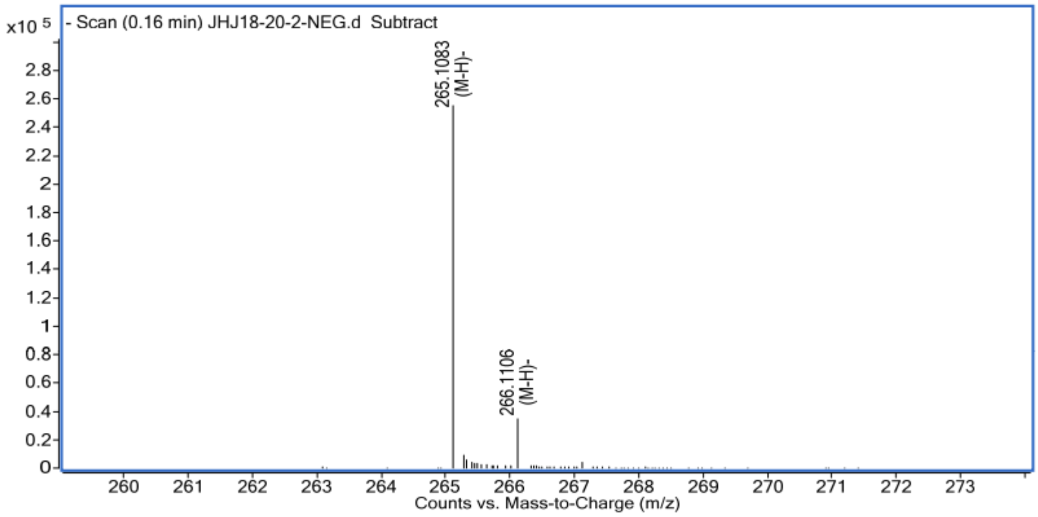

Name	Formula	Score	Mass	Mass (MFG)	Diff (ppm)	Diff (abs. ppm)	Diff (mDa)
	C14 H18 O5	97.2	266.1156	266.11542	-0.67	0.67	-0.18

Species	Ion Formula	m/z	Height	Score (MFG)	Score (MFG, MS)	Score (MFG, mass)	Score (MFG, abund)	Score (MFG, iso. spacing)
(M-H)-	C14 H17 O5	265.1081	254862.1	97.2	97.2	99.74	96.82	92.57

m/z	m/z (Calc)	Diff (ppm)	Diff (mDa)	Height	Height (Calc)	Height %	Height % (Calc)	Height Sum %	Height Sum% (Calc)
265.1083	265.1081	-0.68	-0.2	254862.1	250001.9	100	100	86.6	85
266.1106	266.1115	3.47	0.9	34940.5	38820.3	13.7	15.5	11.9	13.2
267.1142	267.1137	-1.93	-0.5	4397.5	5377.9	1.7	2.2	1.5	1.8

Figure S1. HRESIMS of raistrickione A (**1**)

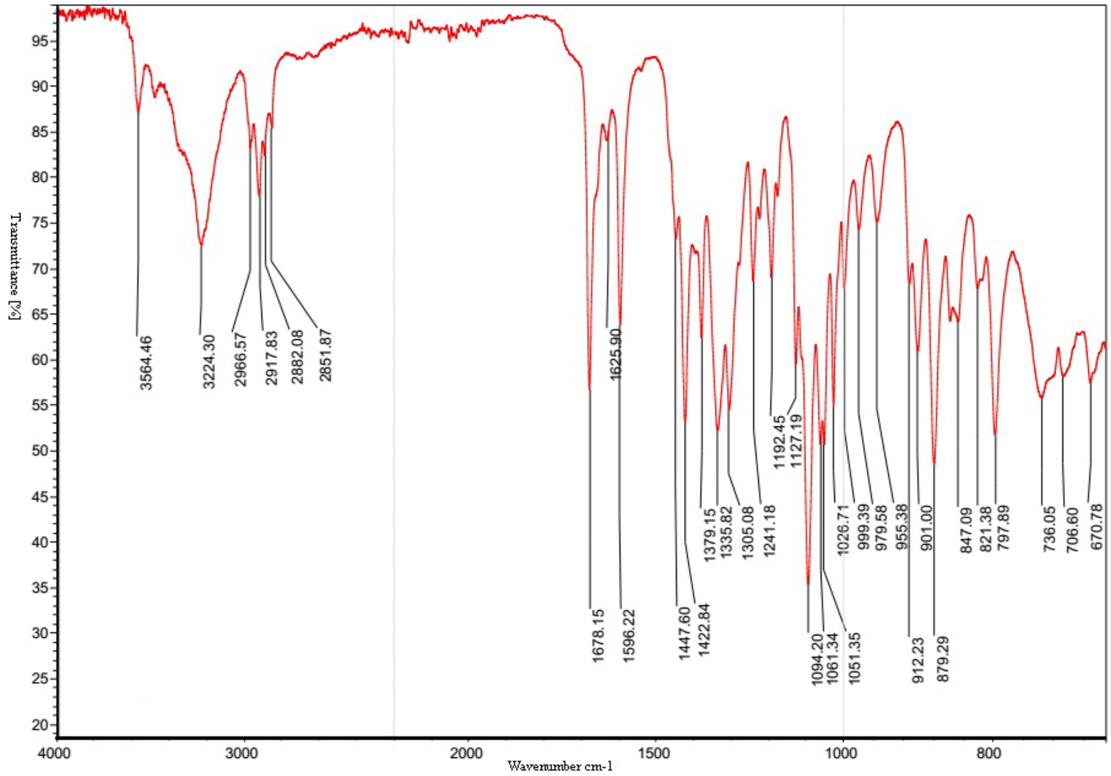

Figure S2. IR spectrum (ATR approach) of raistrickione A (**1**)

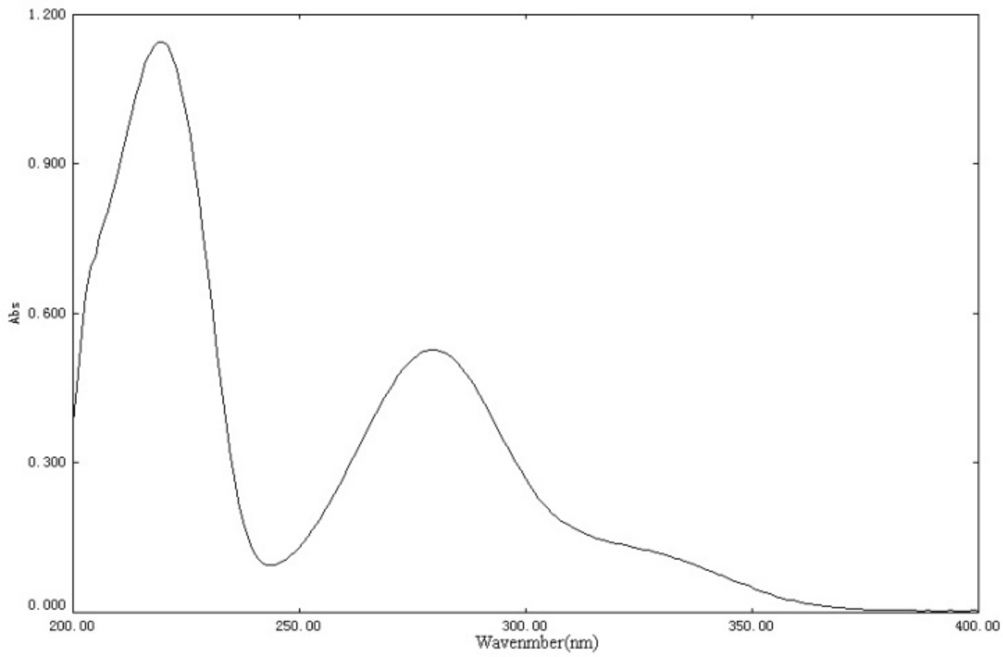

Figure S3. UV spectrum (MeOH) of raistrickione A (**1**)

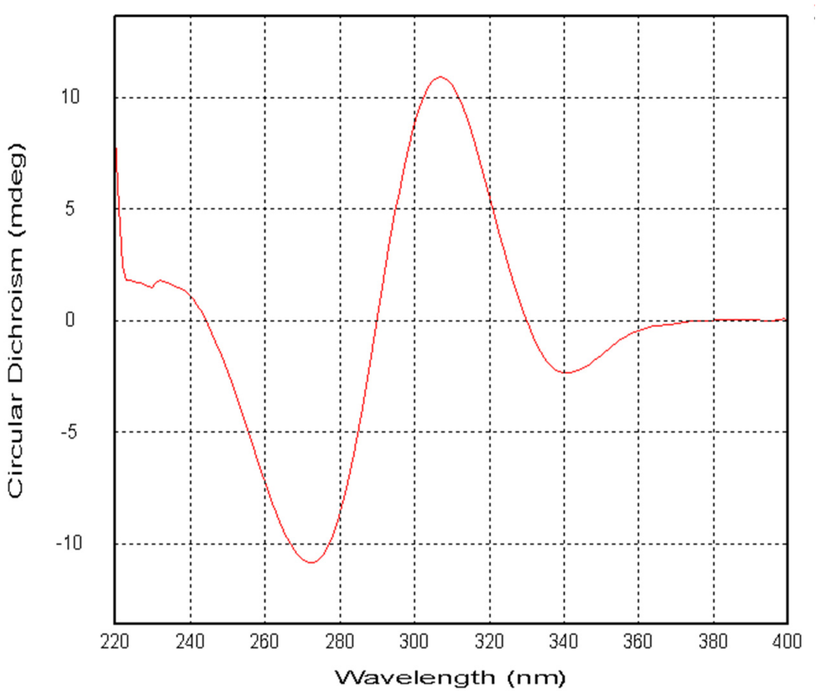

Figure S4. ECD spectrum (MeOH) of raistrickione A (**1**)

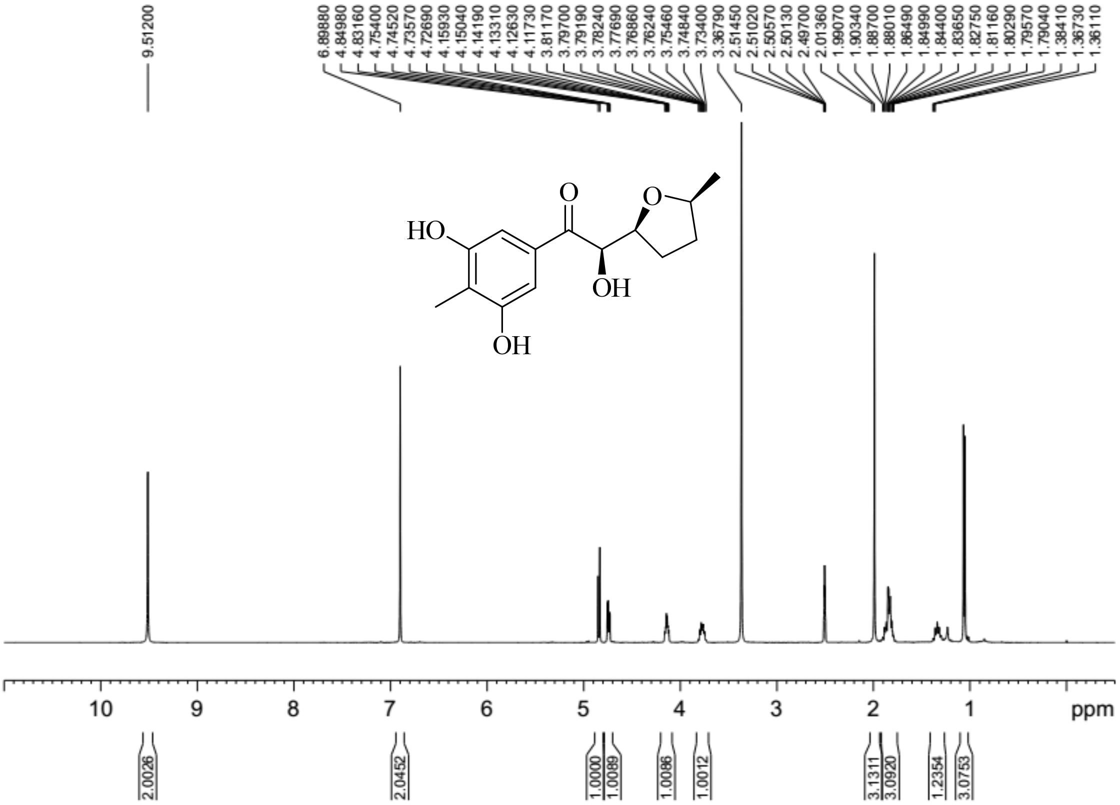

Figure S5. ^{1}H NMR spectrum (400 MHz DMSO-d_6) of raistrickione A (**1**)

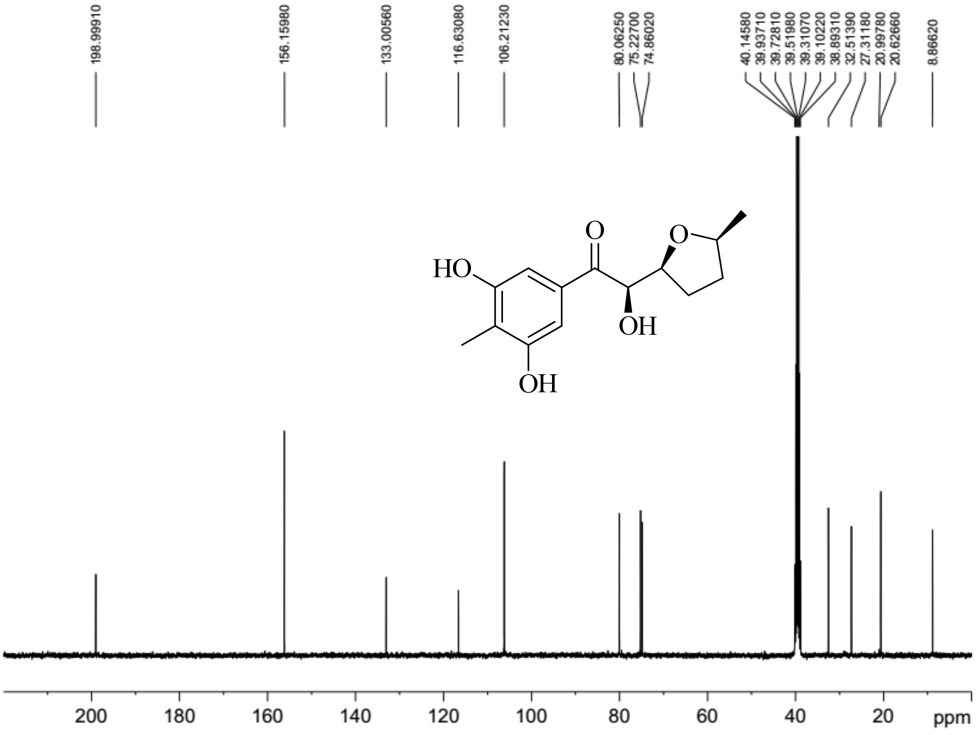

Figure S6. ^{13}C NMR spectrum (100 MHz DMSO-d_6) of raistrickione A (**1**)

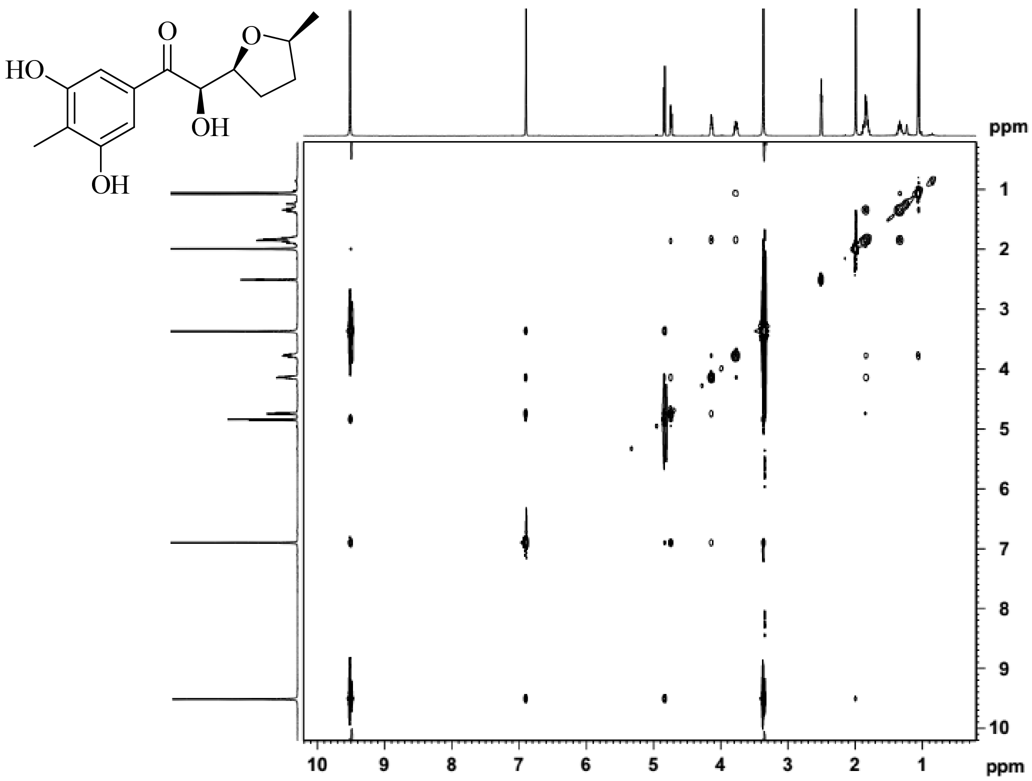

Figure S7. NOSEY spectrum (DMSO-d_6) of raistrickione A (**1**)

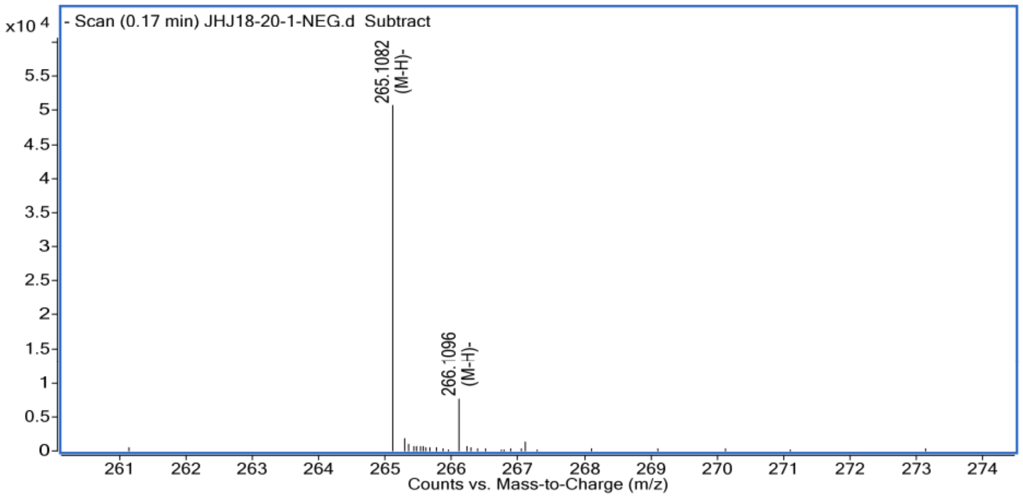

Name	Formula	Score	Mass	Mass (MFG)	Diff (ppm)	Diff (abs. ppm)	Diff (mDa)
	C14 H18 O5	95.13	266.11546	266.11542	-0.14	0.14	-0.04

Species	Ion Formula	m/z	Height	Score (MFG)	Score (MFG, MS)	Score (MFG, mass)	Score (MFG, abund)	Score (MFG, iso. spacing)
(M-H)-	C14 H17 O5	265.1081	50685.9	95.13	95.13	99.99	99.48	80.19

m/z	m/z (Calc)	Diff (ppm)	Diff (mDa)	Height	Height (Calc)	Height %	Height % (Calc)	Height Sum %	Height Sum% (Calc)
265.1082	265.1081	-0.16	0	50685.9	50699.5	100	100	85	85
266.1096	266.1115	7.15	1.9	7657.8	7872.6	15.1	15.5	12.8	13.2
267.116	267.1137	-8.44	-2.3	1319.1	1090.6	2.6	2.2	2.2	1.8

Figure S8. HRESIMS of raistrickione B (**2**)

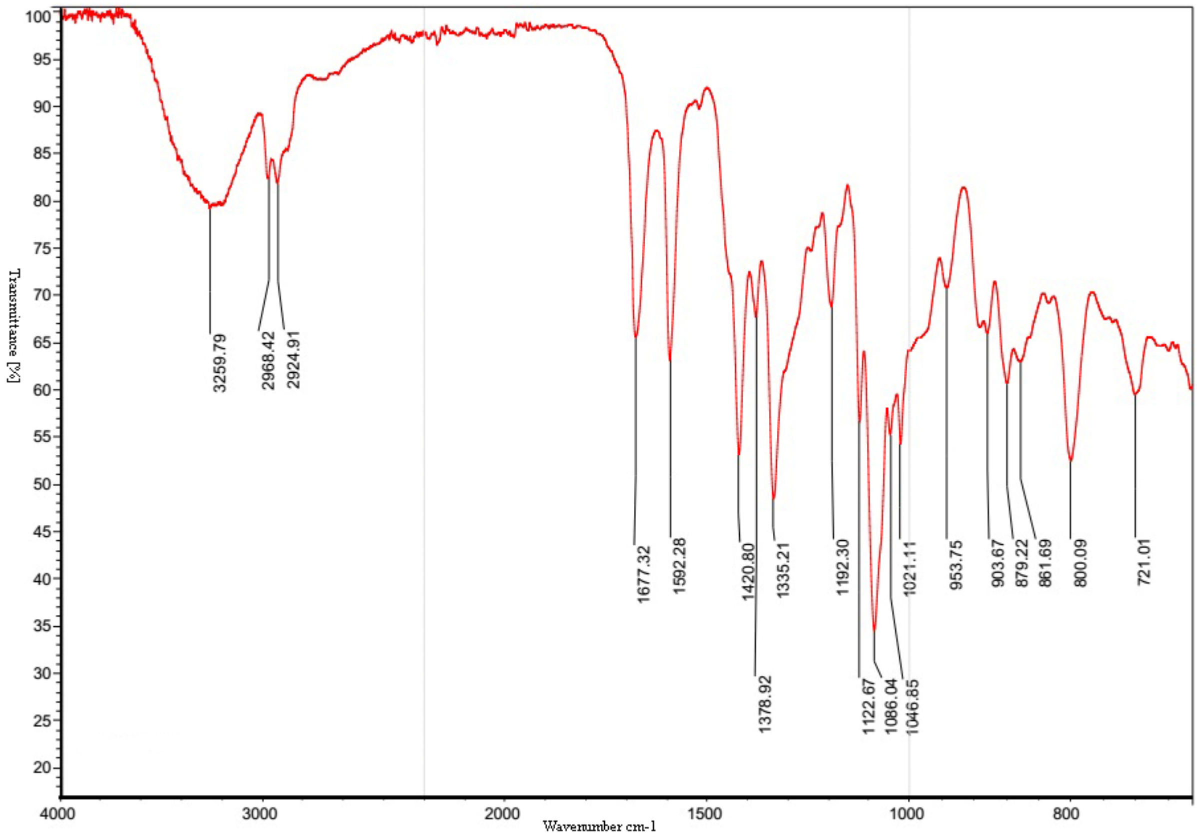

Figure S9. IR spectrum (ATR approach) of raistrickione B (**2**)

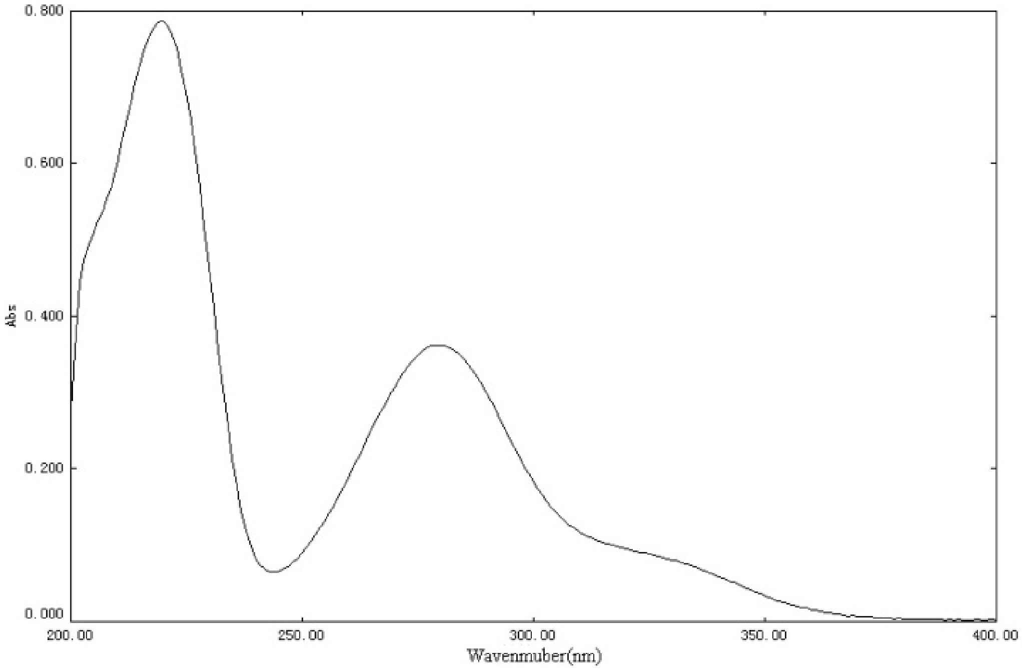

Figure S10. UV spectrum (MeOH) of raistrickione B (**2**)

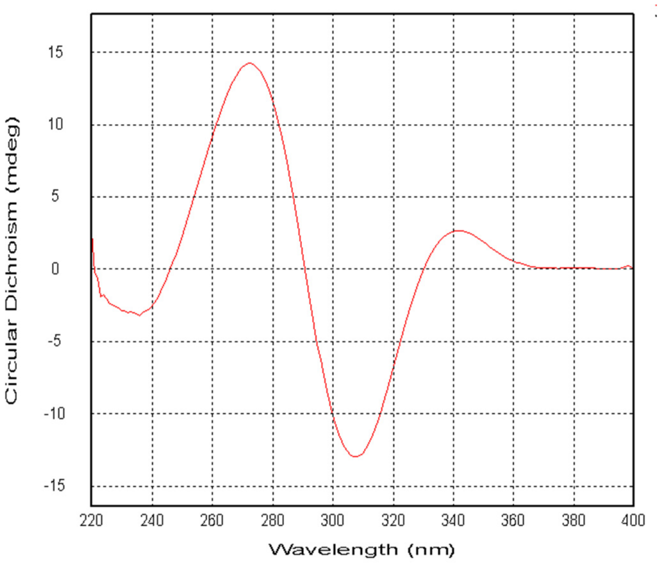

Figure S11. ECD spectrum (MeOH) of raistrickione B (**2**)

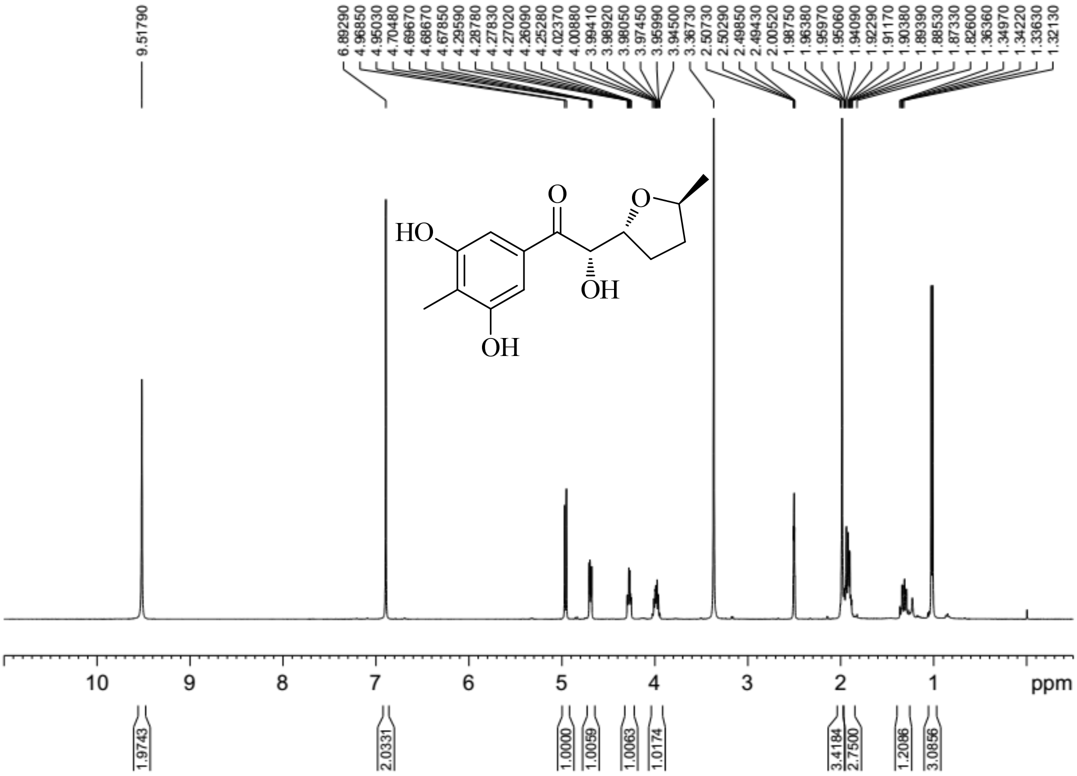

Figure S12. ^{1}H NMR spectrum (400 MHz DMSO-d_6) of raistrickione B (**2**)

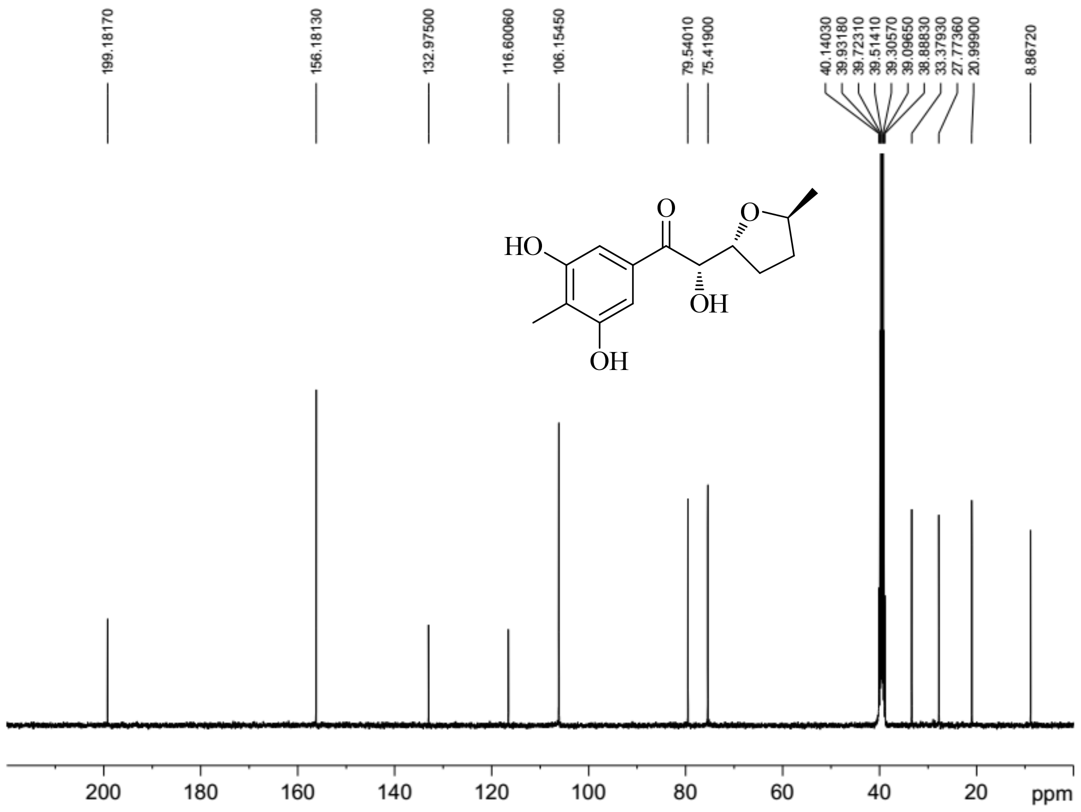

Figure S13. ^{13}C NMR spectrum (100 MHz DMSO-d_6) of raistrickione B (**2**)

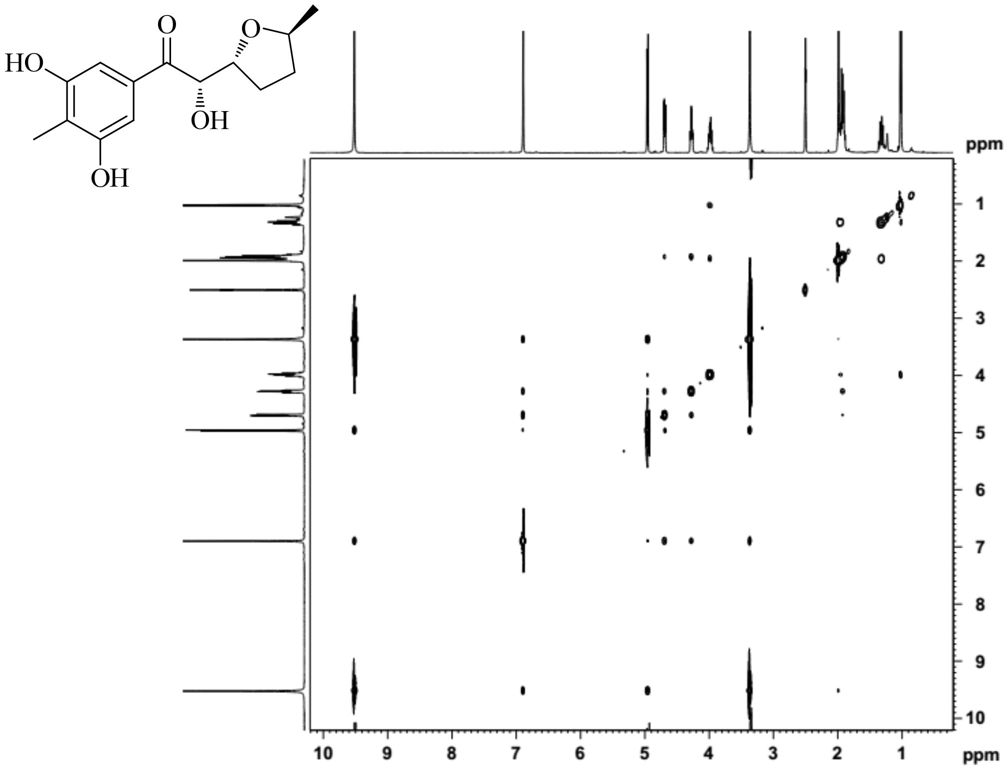

Figure S14. NOSEY spectrum (DMSO-d_6) of raistrickione B (**2**)

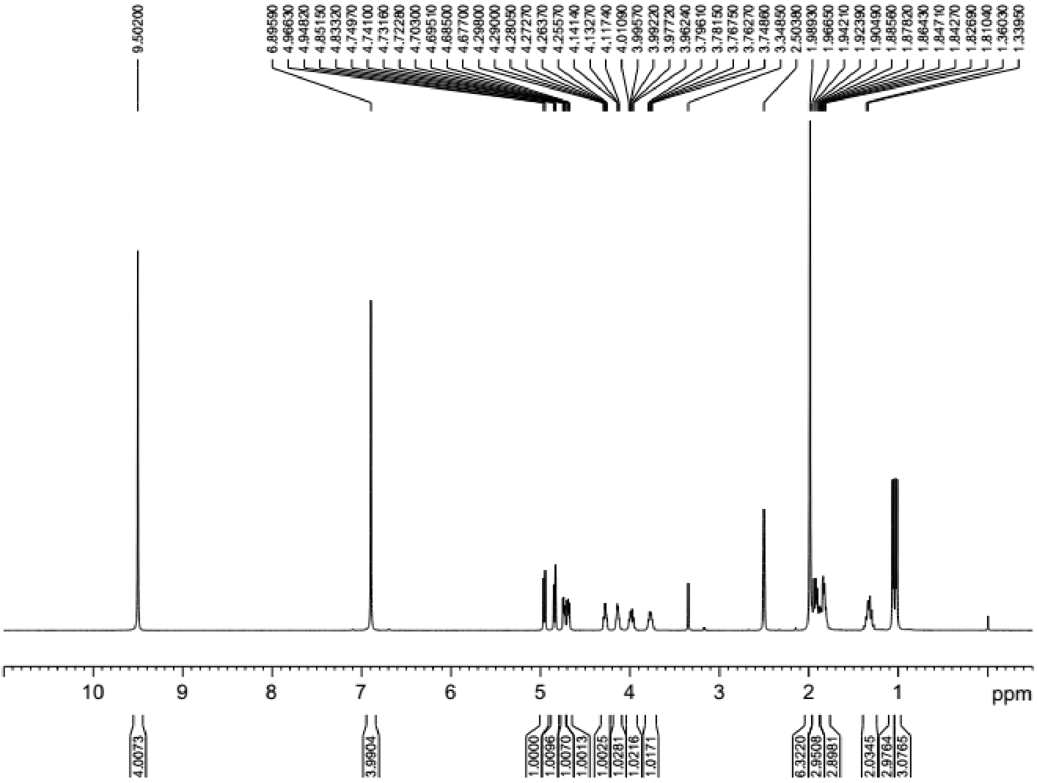

Figure S15. ^{1}H NMR spectrum (400 MHz DMSO-d_6) of the diastereoisomeric mixture (**1** and **2**)

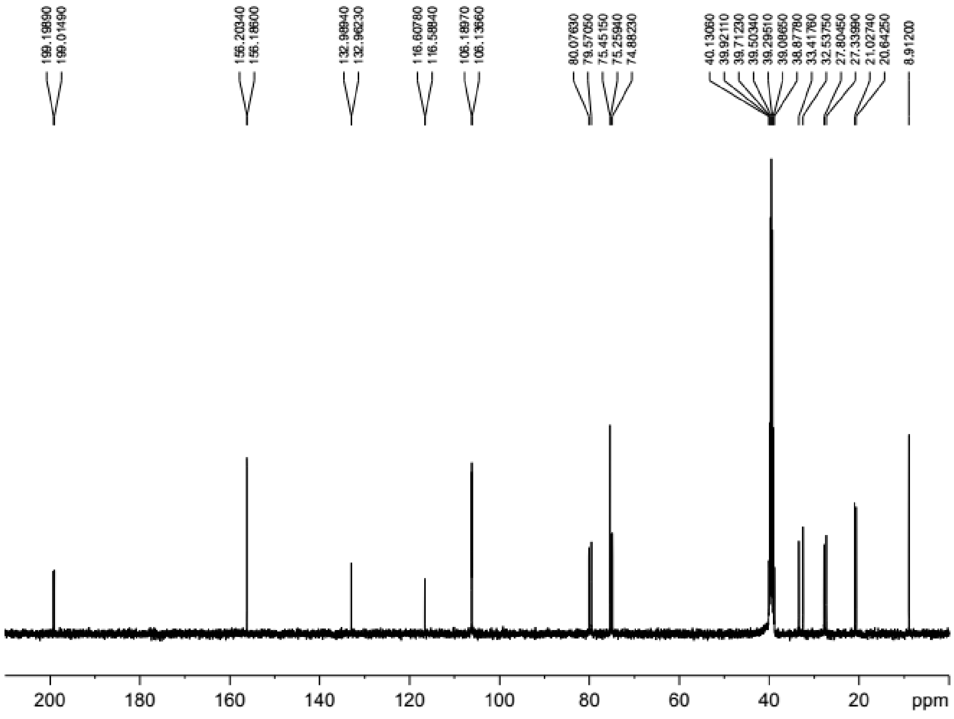

Figure S16. ^{13}C NMR spectrum (100 MHz DMSO-d_6) of the diastereoisomeric mixture (**1** and **2**)

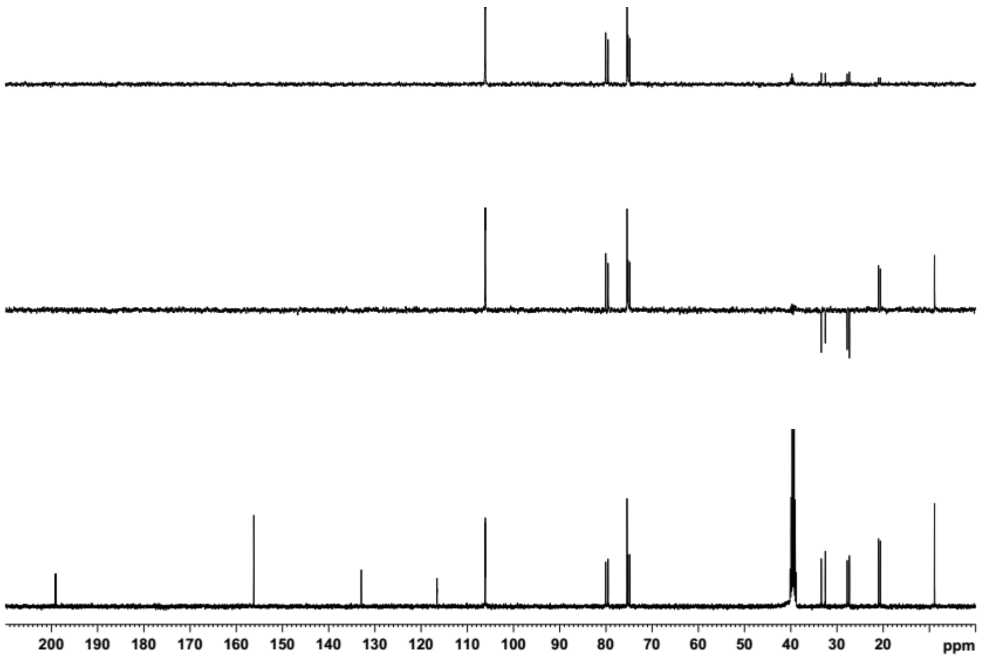

Figure **S17.** DEPT of the diastereoisomeric mixture (**1** and **2**)

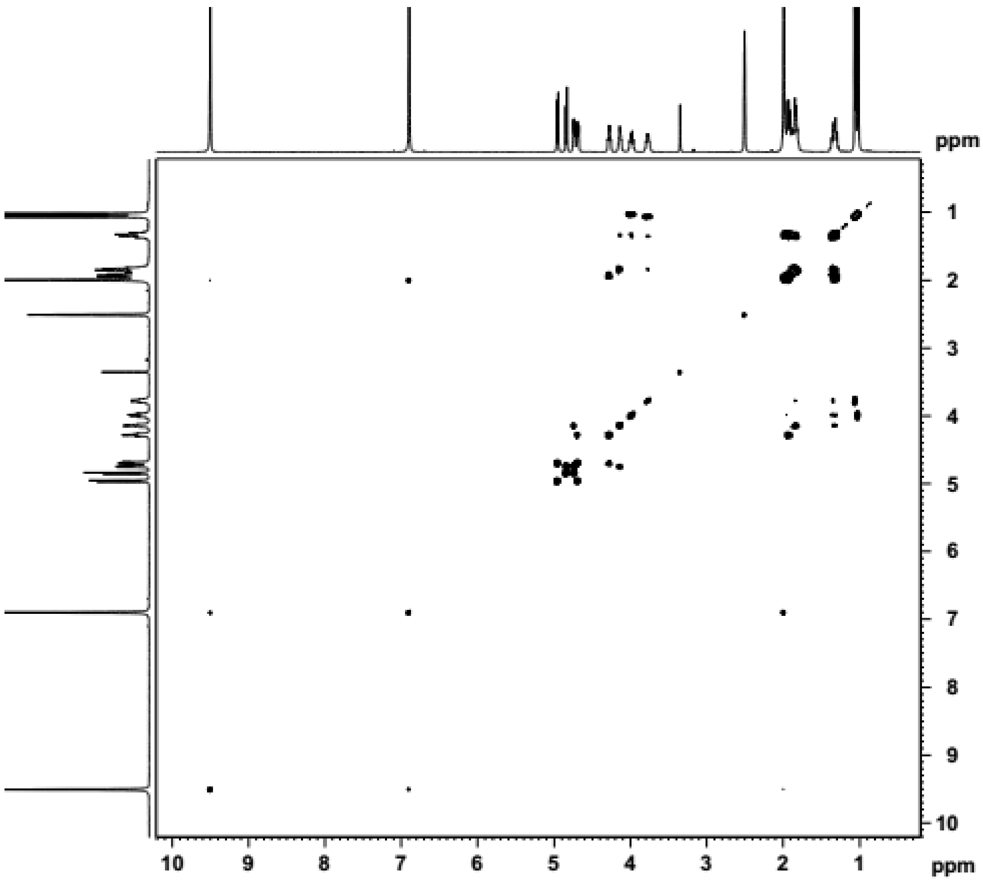

Figure **S18.** COSY of the diastereoisomeric mixture (**1** and **2**)

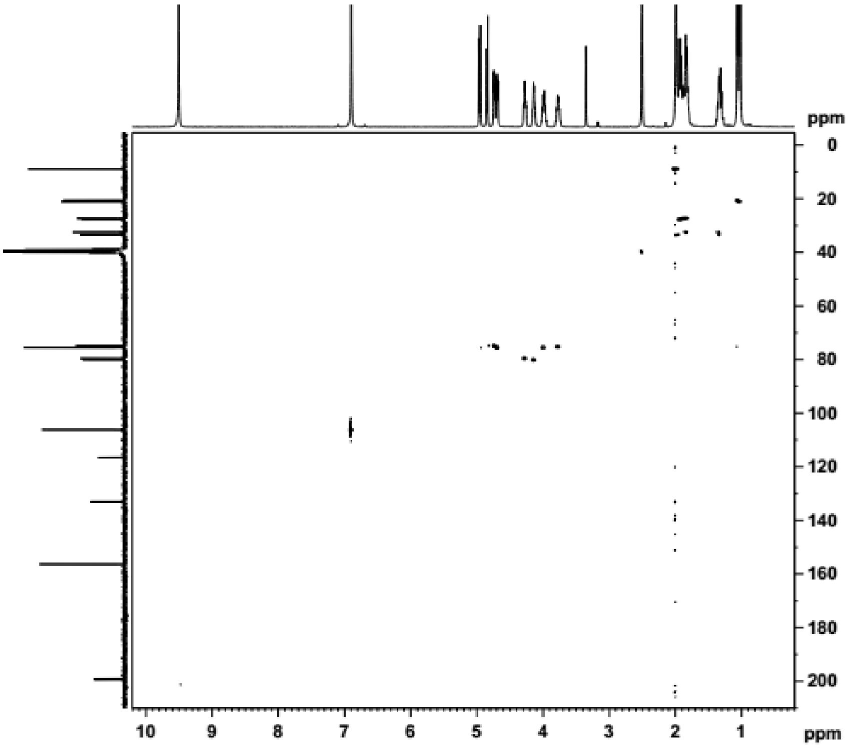

Figure S19. HSQC of the diastereoisomeric mixture (**1** and **2**)

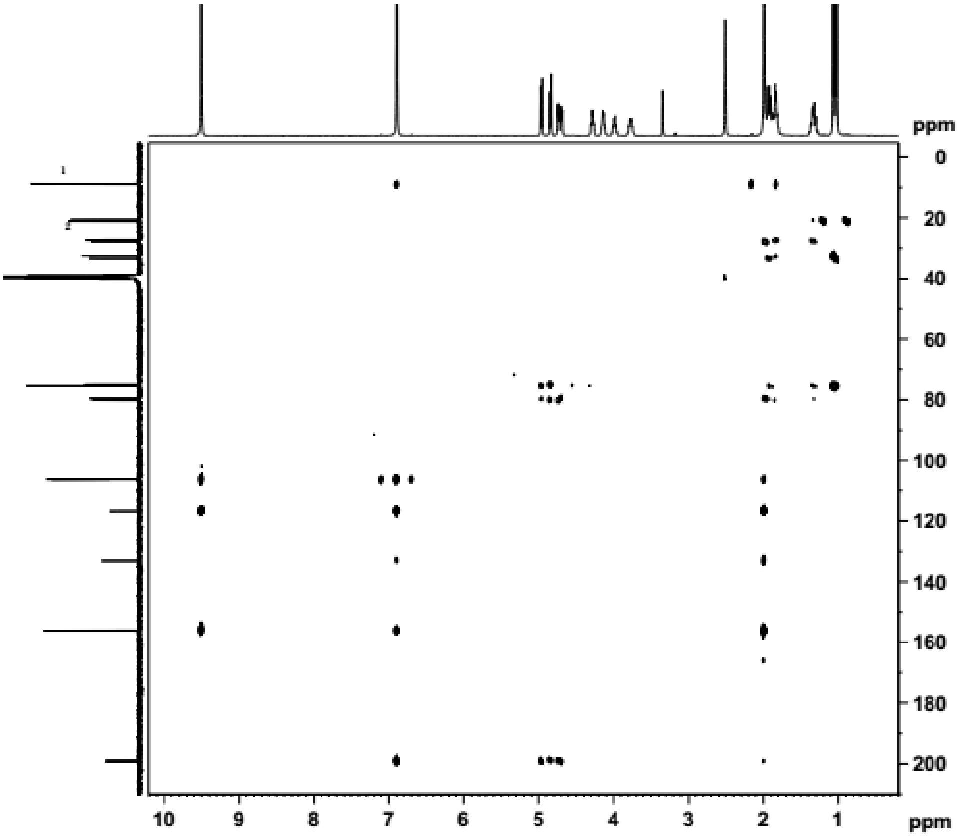

Figure S20. HMBC of the diastereoisomeric mixture (**1** and **2**)

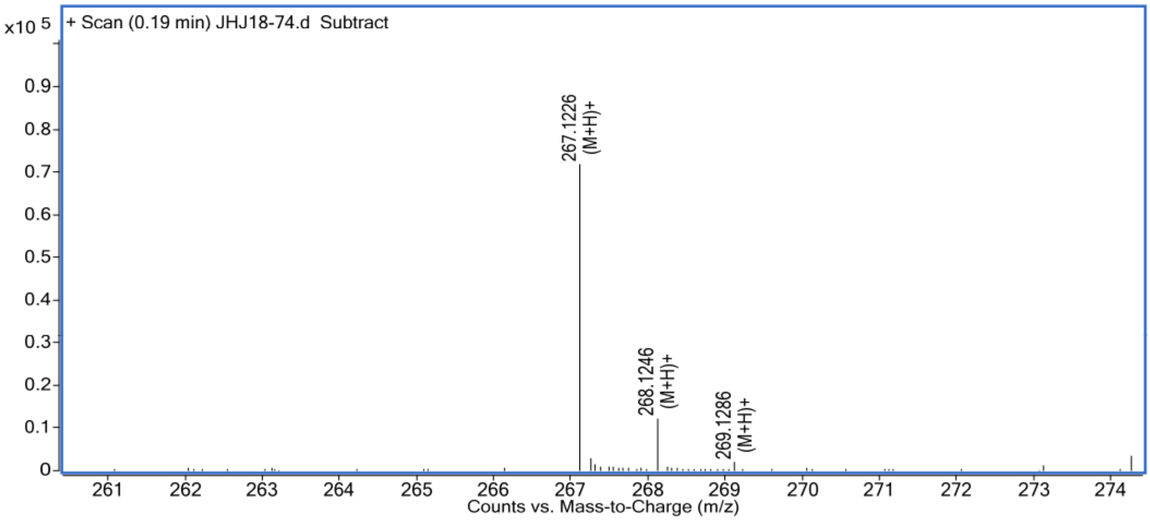

Name	Formula	Score	Mass	Mass (DB)	Mass (MFG)	Diff (ppm)	Diff (abs. ppm)	Diff (mDa)
	C14 H18 O5	96.97	266.11529		266.11542	0.52	0.52	0.14

Species	Ion Formula	m/z	Height	Score (MFG)	Score (MFG, MS)	Score (MFG, mass)	Score (MFG, abund)	Score (MFG, iso. spacing)
(M+H)+	C14 H19 O5	267.1227	71662.1	96.97	96.97	99.84	98.17	89.78

m/z	m/z (Calc)	Diff (ppm)	Diff (mDa)	Height	Height (Calc)	Height %	Height % (Calc)	Height Sum %	Height Sum% (Calc)
267.1226	267.1227	0.49	0.1	71662.1	72757.9	100	100	83.7	85
268.1246	268.1261	5.44	1.5	12091.1	11314.6	16.9	15.6	14.1	13.2
269.1286	269.1283	-1.21	-0.3	1886.9	1567.7	2.6	2.2	2.2	1.8

Figure S21. HRESIMS of raistrickione C (**3**)

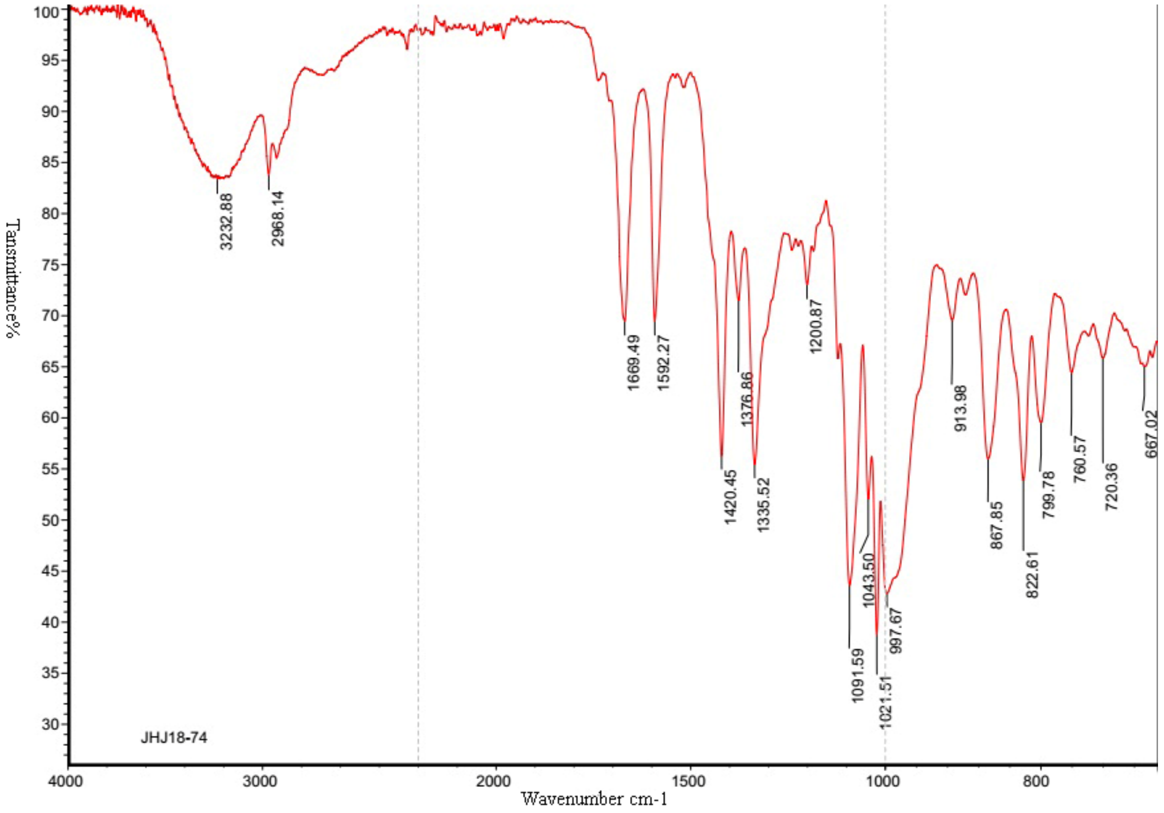

Figure S22. IR spectrum (ATR approach) of raistrickione C (**3**)

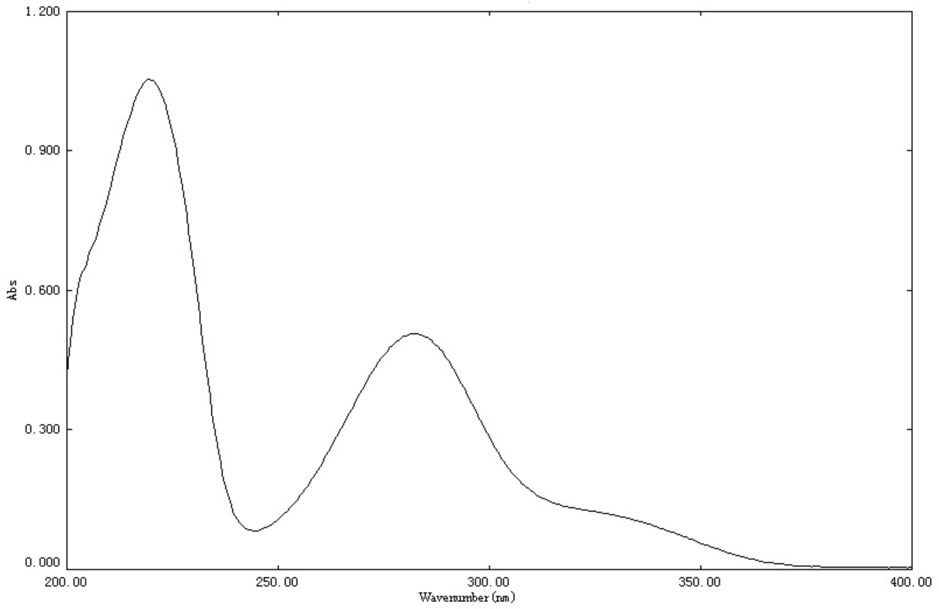

Figure S23. UV spectrum (MeOH) of raistrickione C (**3**)

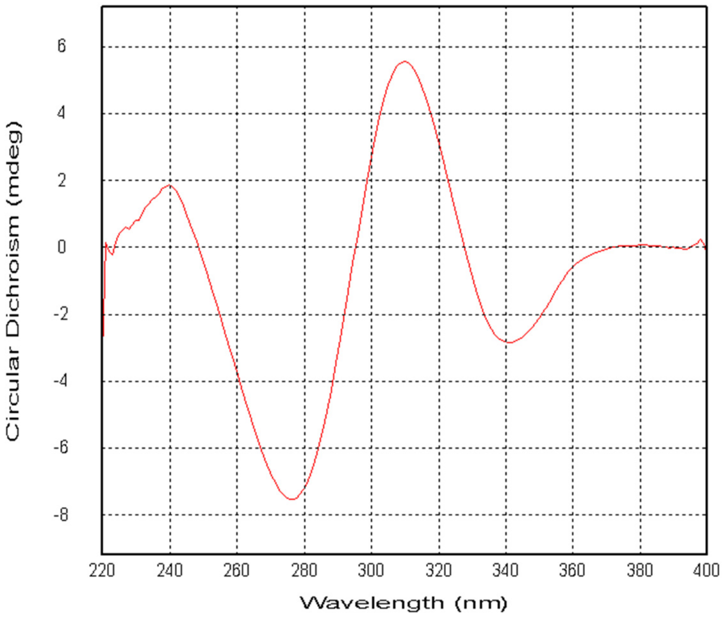

Figure S24. ECD spectrum (MeOH) of raistrickione C (**3**)

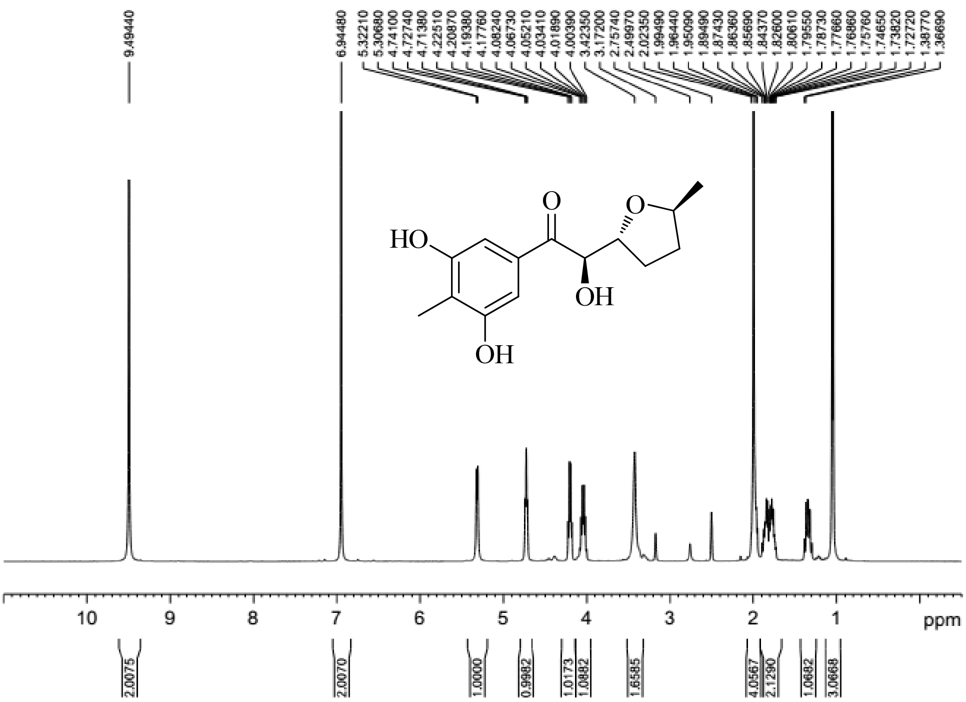

Figure S25. ^{1}H NMR spectrum (400 MHz DMSO-d_6) of raistrickione C (**3**)

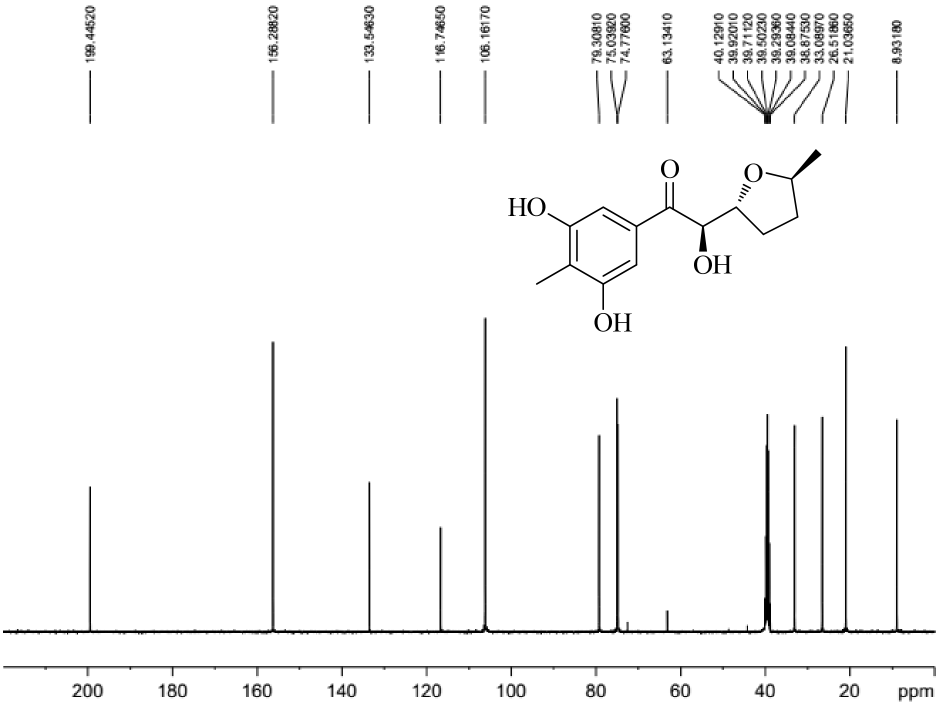

Figure S26. ^{13}C NMR spectrum (100 MHz DMSO-d_6) of raistrickione C (**3**)

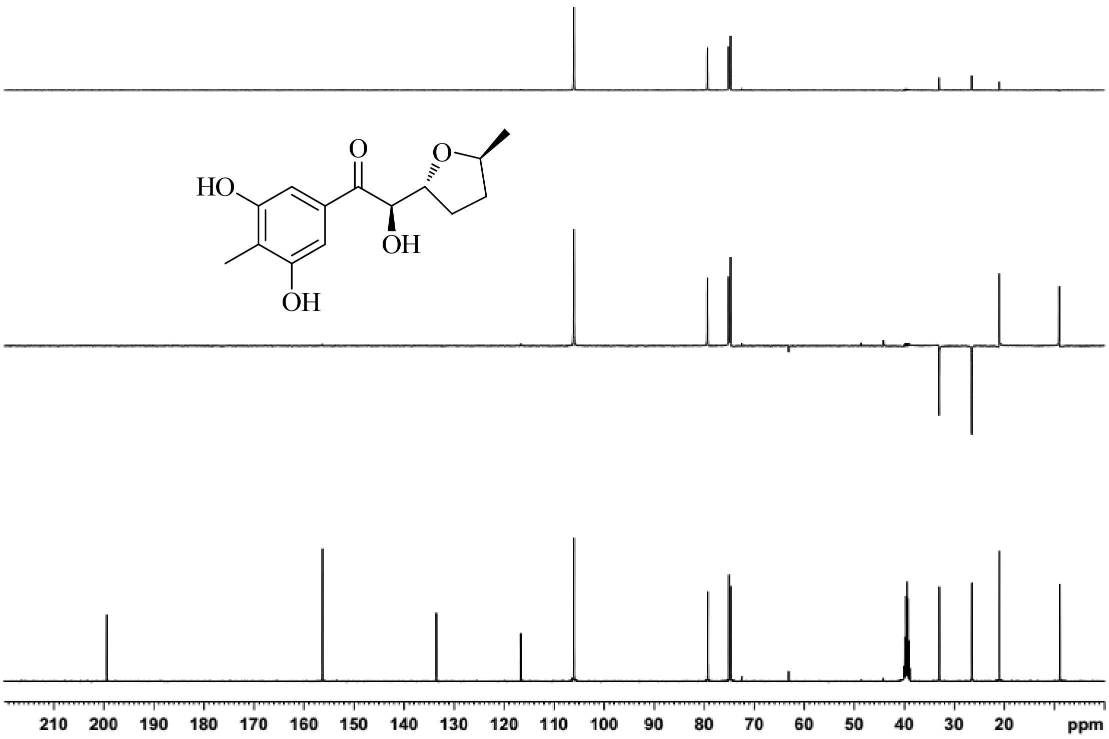

Figure S27. DEPT of raistrickione C (3)

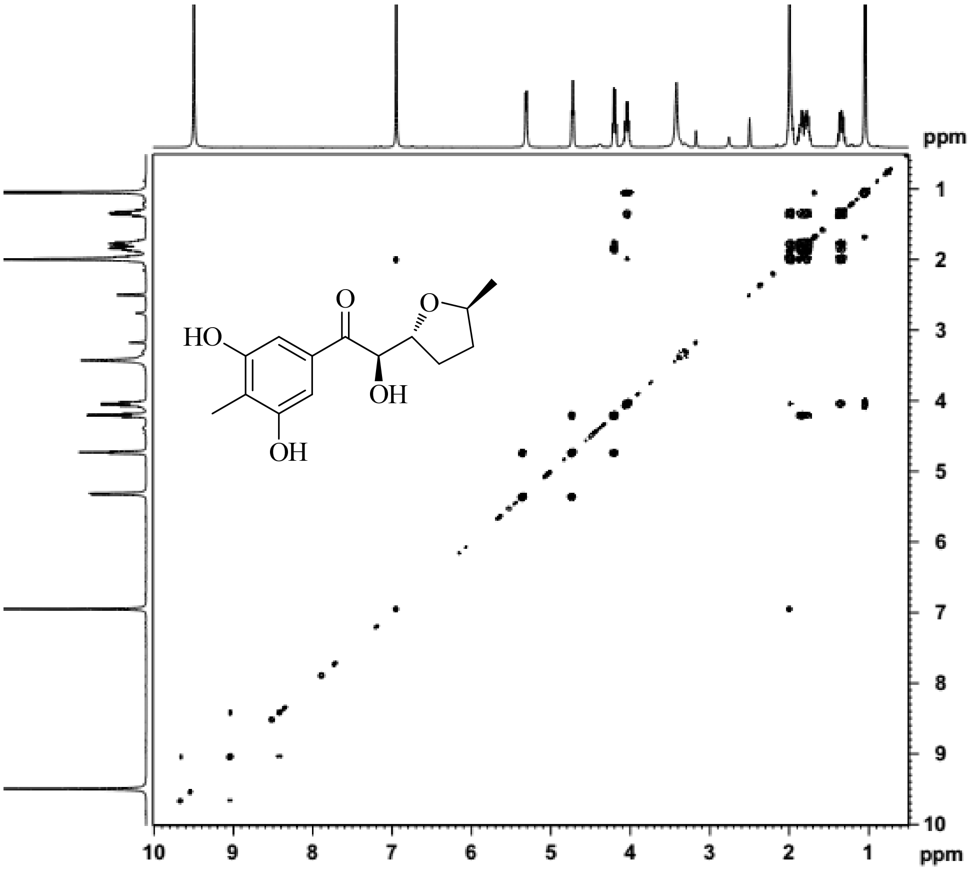

Figure S28. COSY of raistrickione C (3)

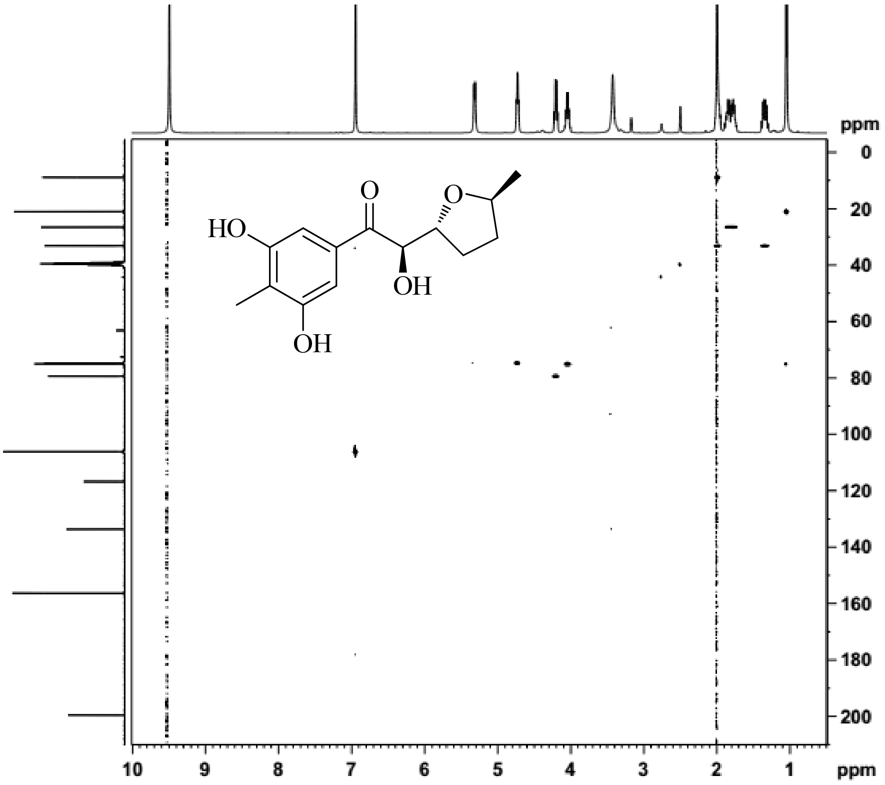

Figure S29. HSQC of raistrickione C (**3**)

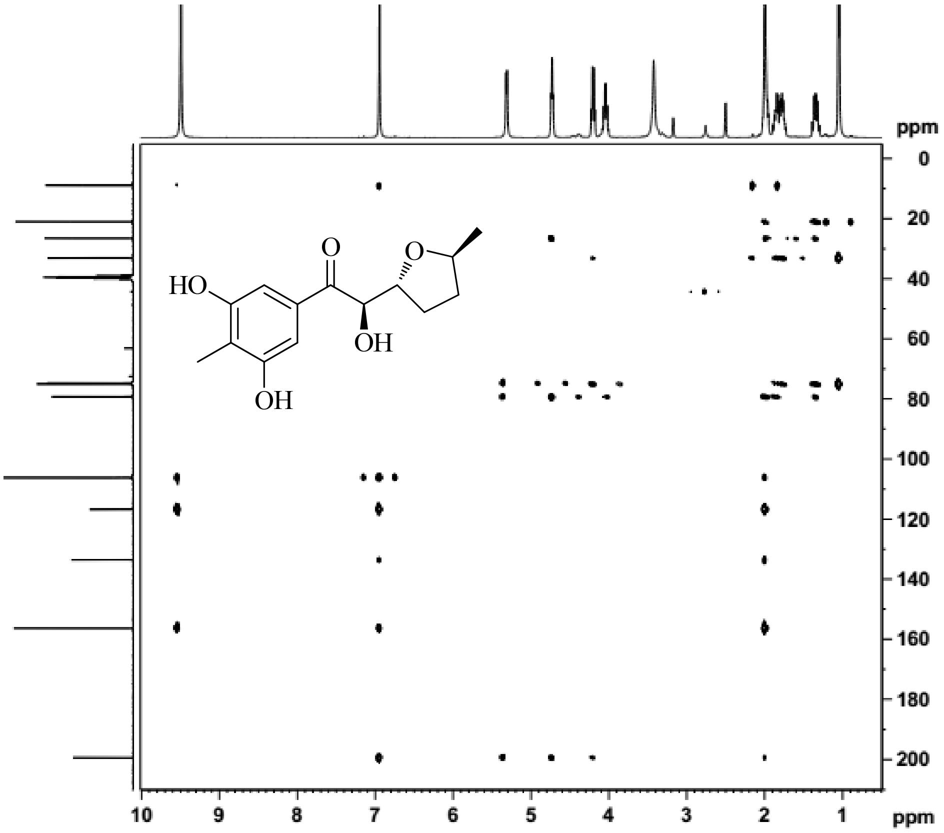

Figure S30. HMBC of raistrickione C (**3**)

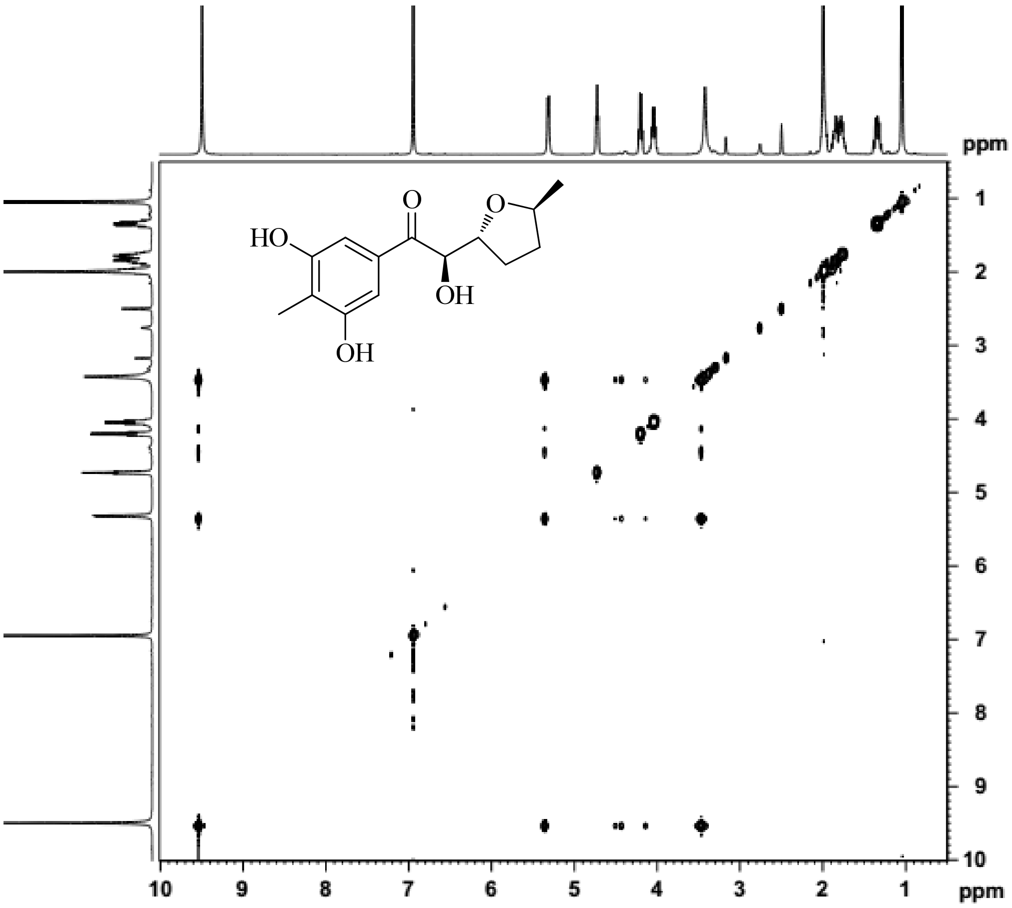

Figure S31. NOESY of raistrickione C (**3**)

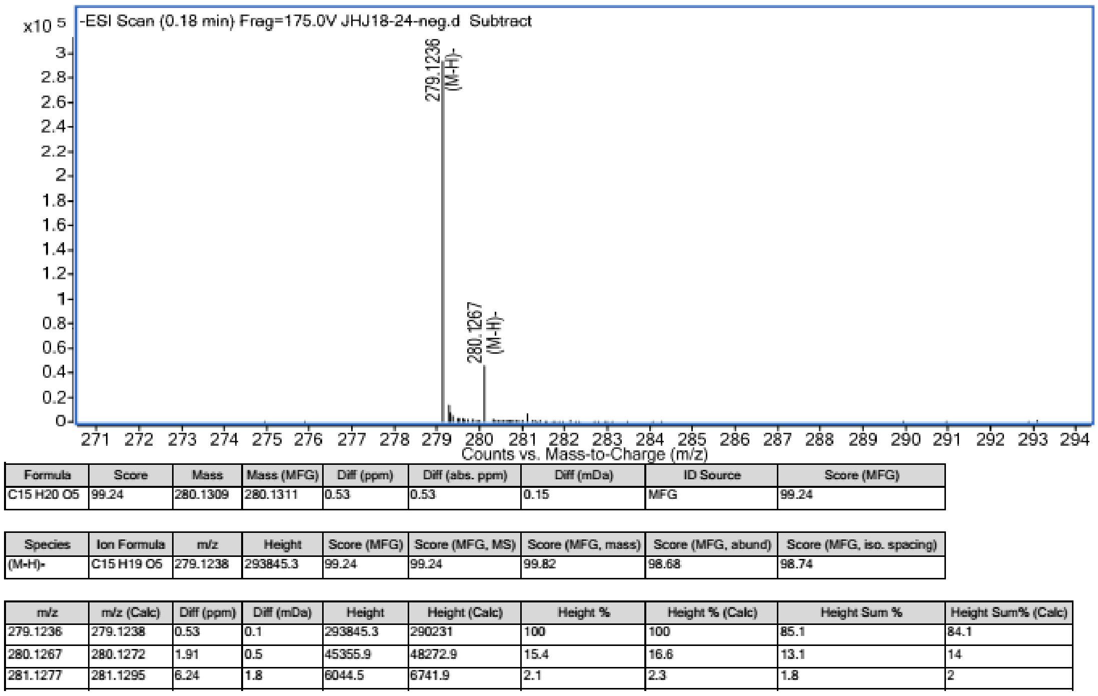

Formula	Score	Mass	Mass (MFG)	Diff (ppm)	Diff (abs. ppm)	Diff (mDa)	ID Source	Score (MFG)
C15 H20 O5	99.24	280.1309	280.1311	0.53	0.53	0.15	MFG	99.24

Species	Ion Formula	m/z	Height	Score (MFG)	Score (MFG, MS)	Score (MFG, mass)	Score (MFG, abund)	Score (MFG, iso. spacing)
(M-H)-	C15 H19 O5	279.1238	293845.3	99.24	99.24	99.82	98.68	98.74

m/z	m/z (Calc)	Diff (ppm)	Diff (mDa)	Height	Height (Calc)	Height %	Height % (Calc)	Height Sum %	Height Sum% (Calc)
279.1236	279.1238	0.53	0.1	293845.3	290231	100	100	85.1	84.1
280.1267	280.1272	1.91	0.5	45355.9	48272.9	15.4	16.6	13.1	14
281.1277	281.1295	6.24	1.8	6044.5	6741.9	2.1	2.3	1.8	2

Figure S32. HRESIMS of raistrickione D (**4**)

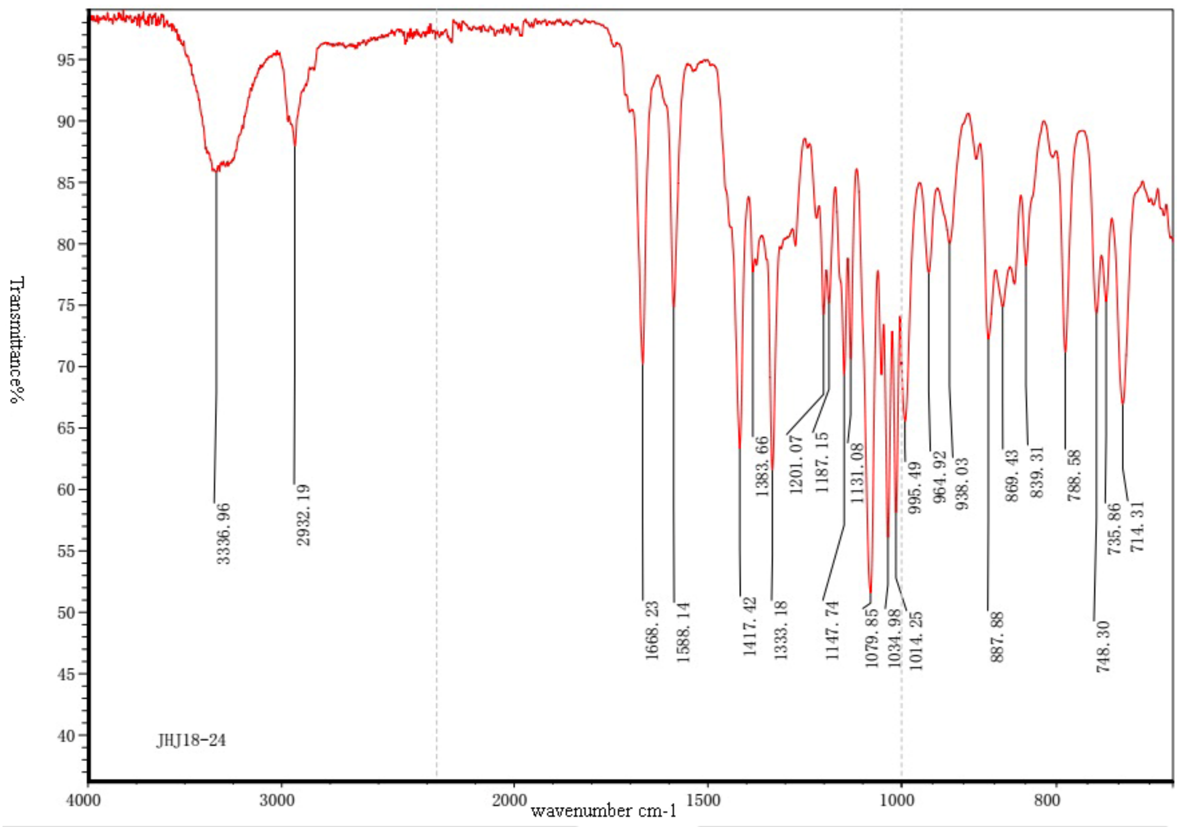

Figure S33. IR spectrum (ATR approach) of raistrickione D (**4**)

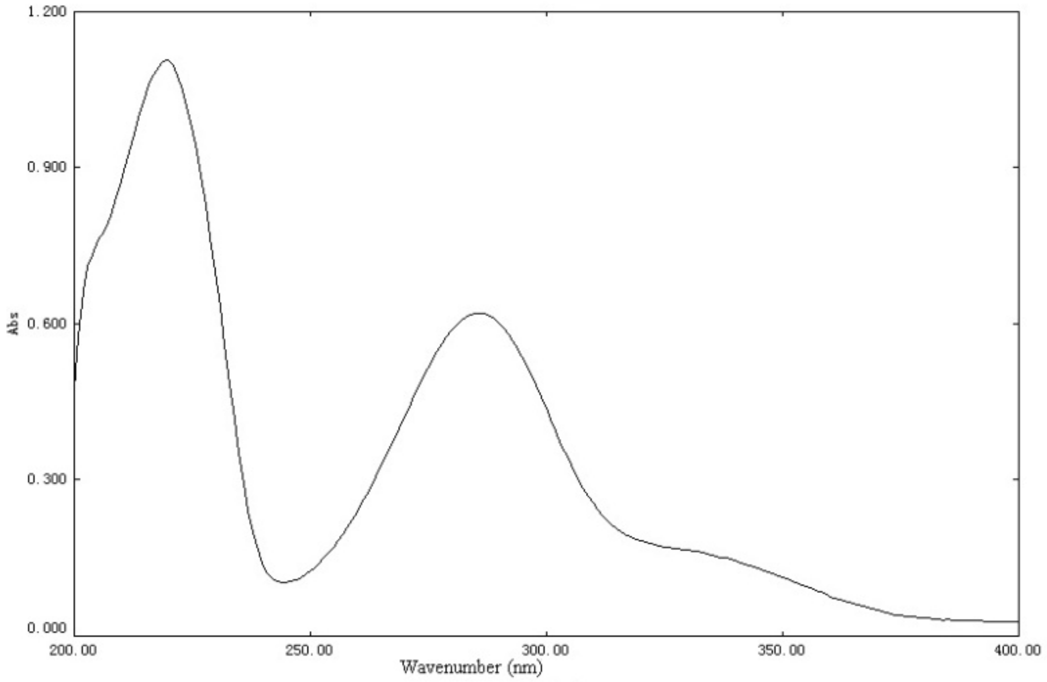

Figure S34. UV spectrum (MeOH) of raistrickione D (**4**)

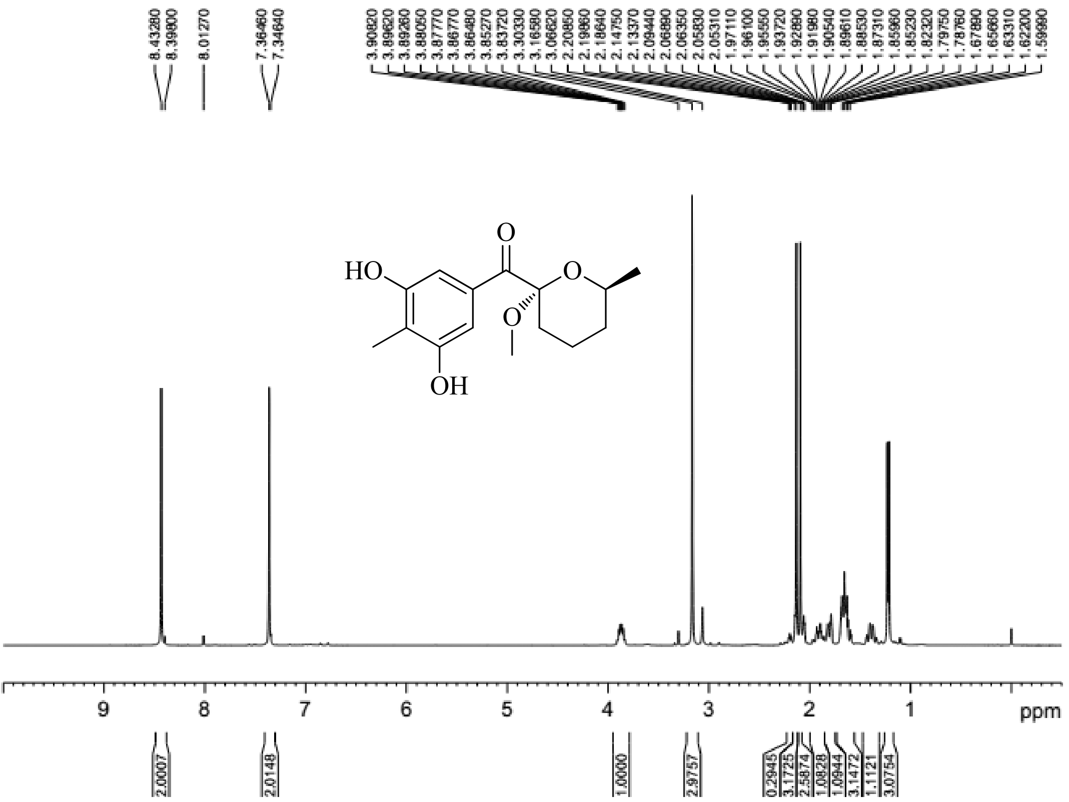

Figure S35. ^{1}H NMR spectrum (400 MHz acetone-d_6) of raistrickione D (**4**)

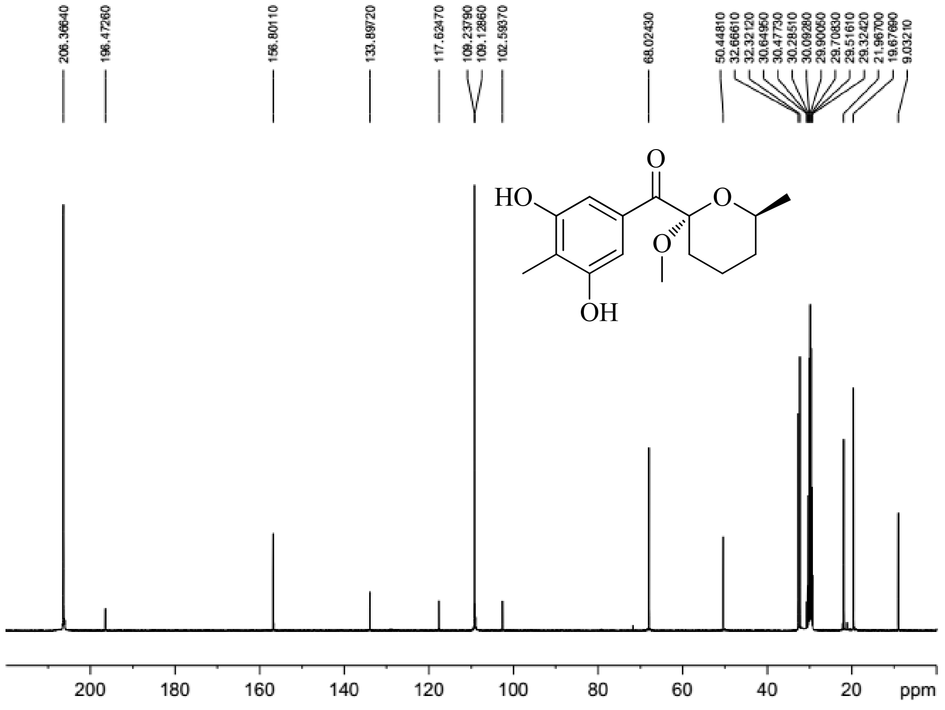

Figure S36. ^{13}C NMR spectrum (100 MHz acetone-d_6) of raistrickione D (**4**)

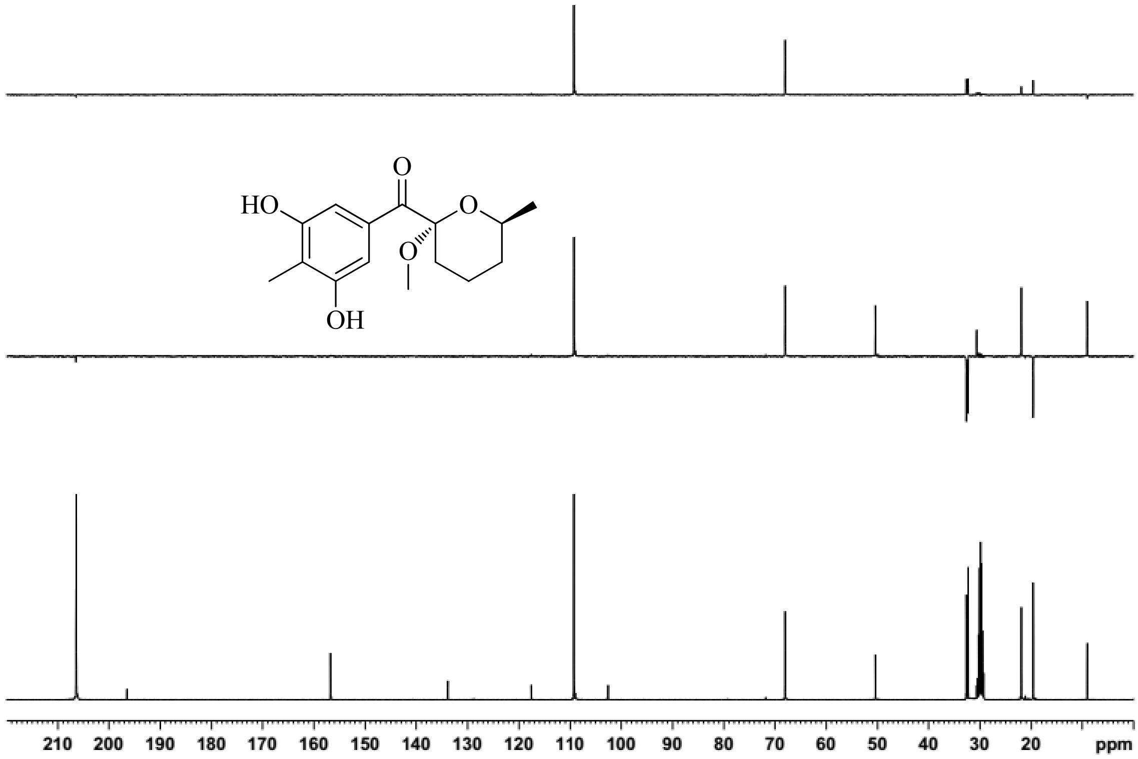

Figure S37. DEPT of raistrickione D (**4**)

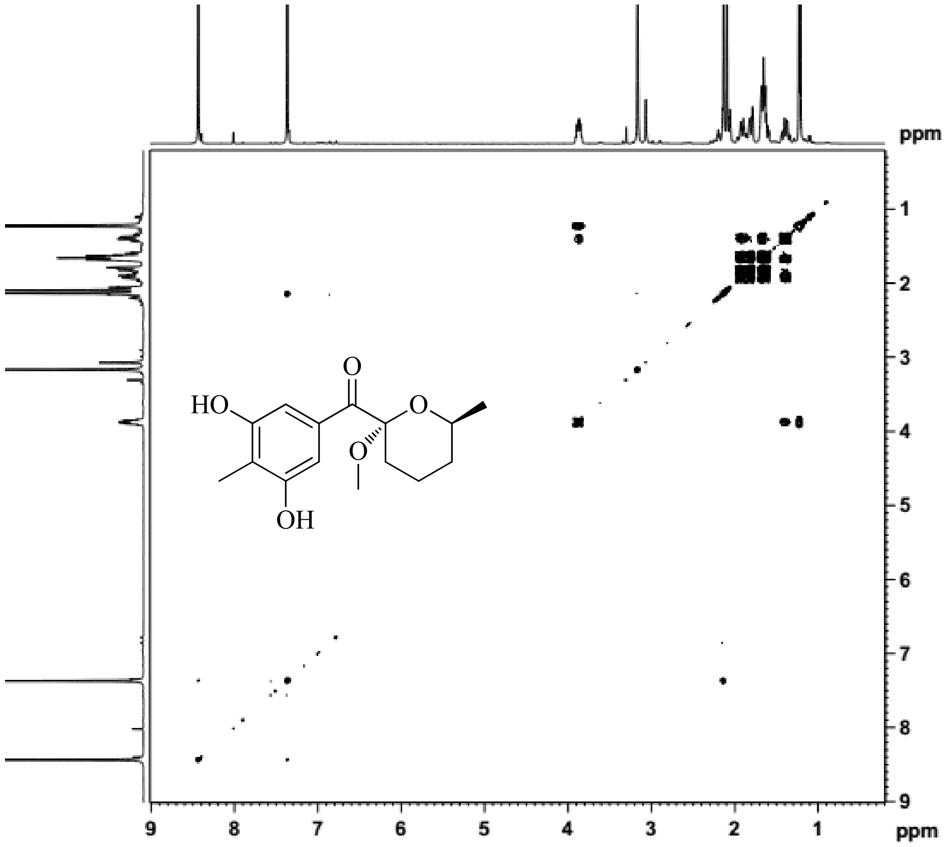

Figure S38. COSY of raistrickione D (**4**)

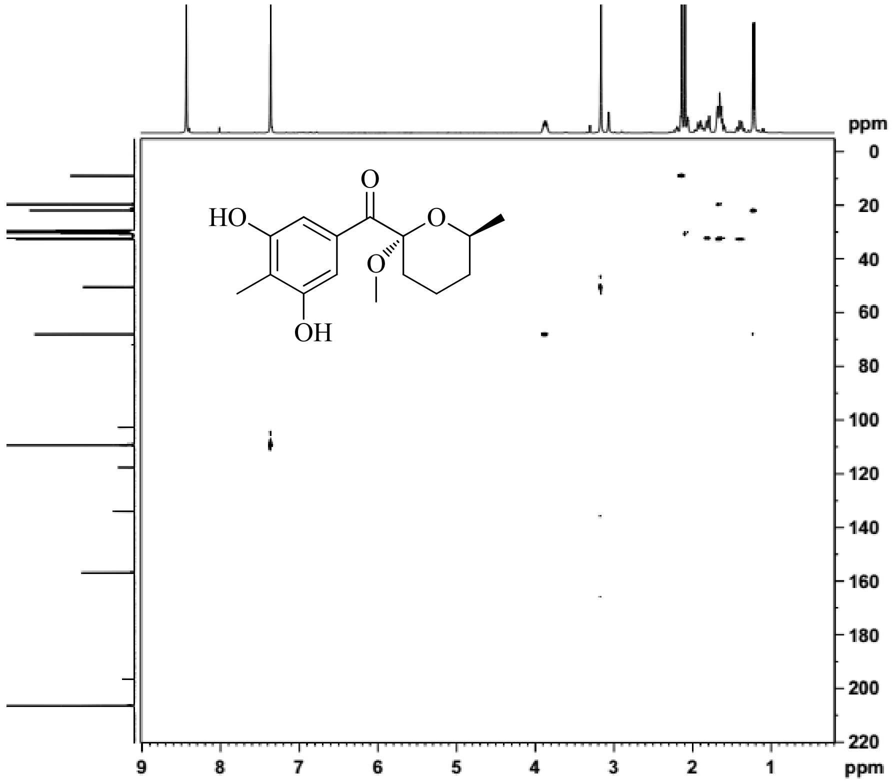

Figure S39. HSQC of raistrickione D (**4**)

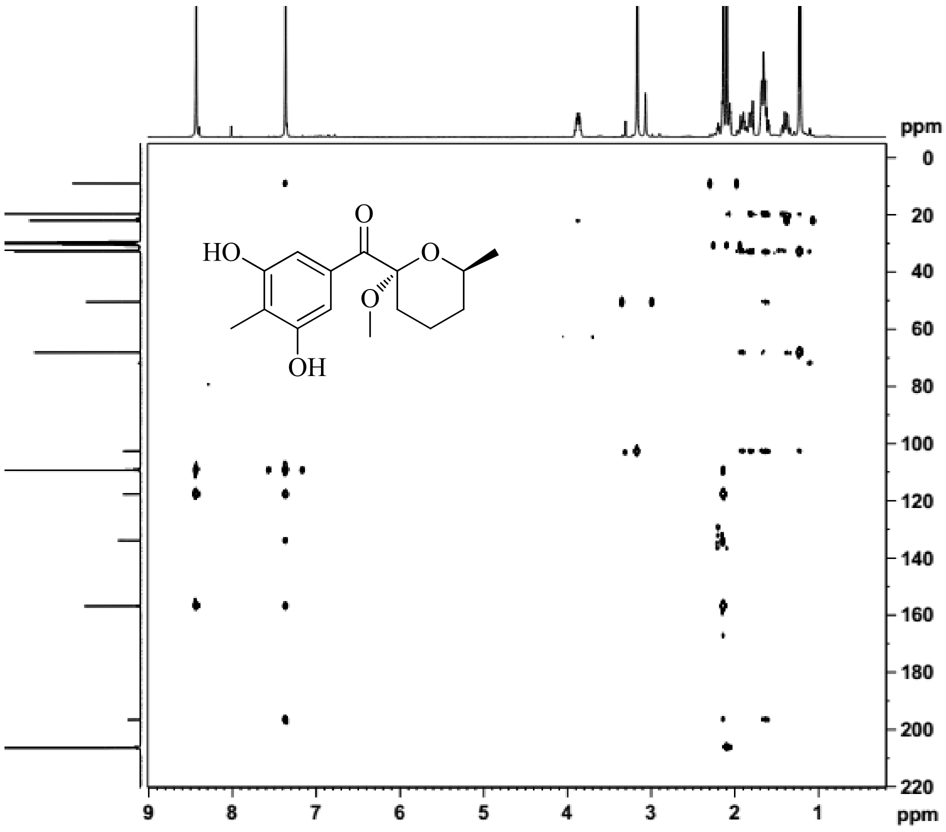

Figure S40. HMBC of raistrickione D (**4**)

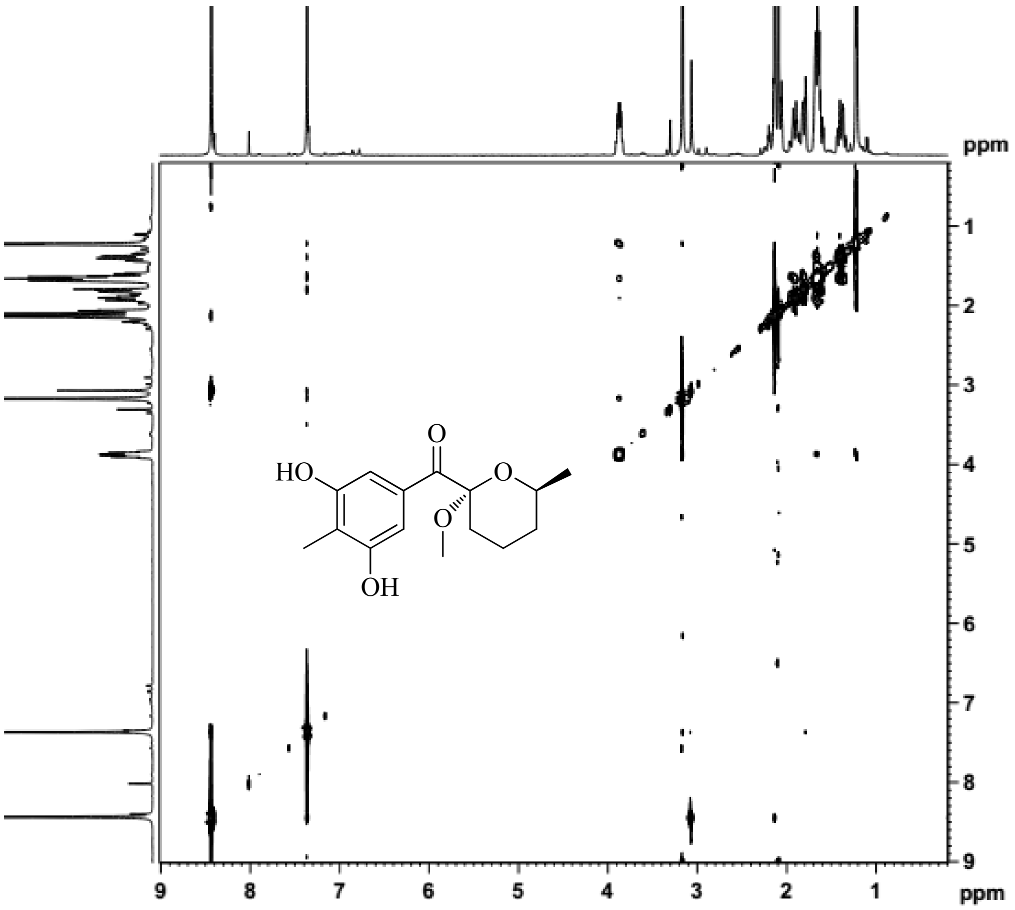

Figure S41. NOSEY of raistrickione D (**4**)

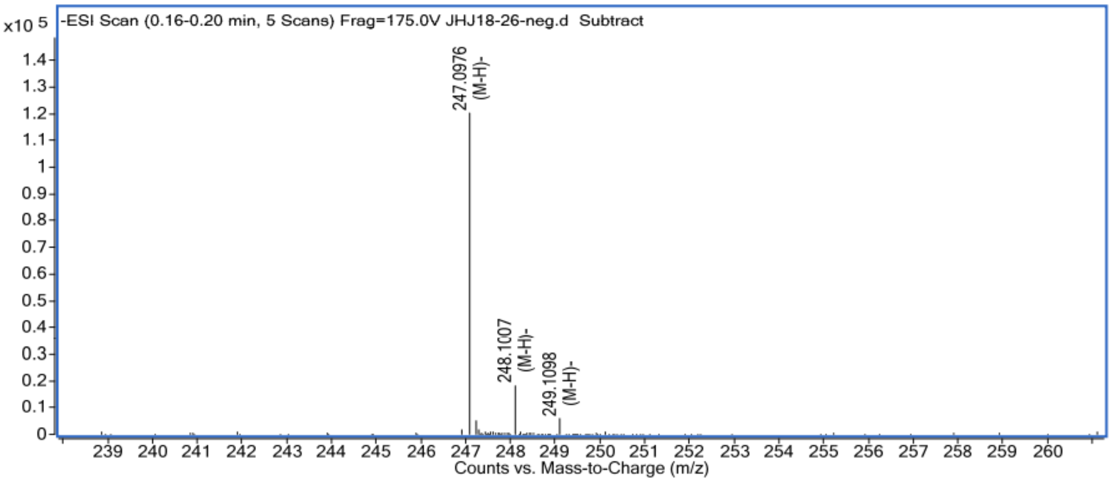

Formula	Score	Mass	Mass (MFG)	Diff (ppm)	Diff (abs. ppm)	Diff (mDa)	ID Source	Score (MFG)
C14 H16 O4	91.64	248.1048	248.1049	0.07	0.07	0.02	MFG	91.64

Species	Ion Formula	m/z	Height	Score (MFG)	Score (MFG, MS)	Score (MFG, mass)	Score (MFG, abund)	Score (MFG, iso. spacing)
(M-H)-	C14 H15 O4	247.0976	120274.9	91.64	91.64	100	89.62	77.35

m/z	m/z (Calc)	Diff (ppm)	Diff (mDa)	Height	Height (Calc)	Height %	Height % (Calc)	Height Sum %	Height Sum% (Calc)
247.0976	247.0976	0.07	0	120274.9	123077.5	100	100	83.2	85.2
248.1007	248.101	1.24	0.3	18327.4	19036.3	15.2	15.5	12.7	13.2
249.1098	249.1033	-25.99	-6.5	5894.6	2383	4.9	1.9	4.1	1.6

Figure S42. HRESIMS of raistrickione E (**5**)

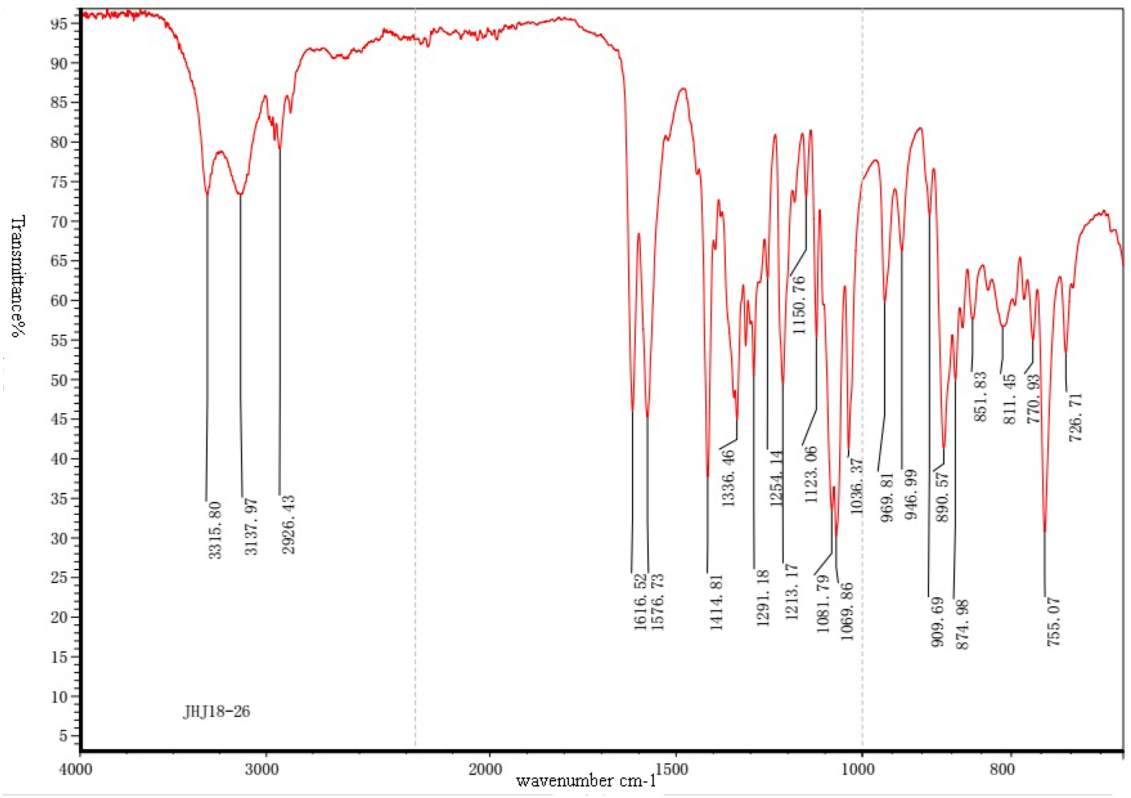

Figure S43. IR spectrum (ATR approach) of raistrickione E (**5**)

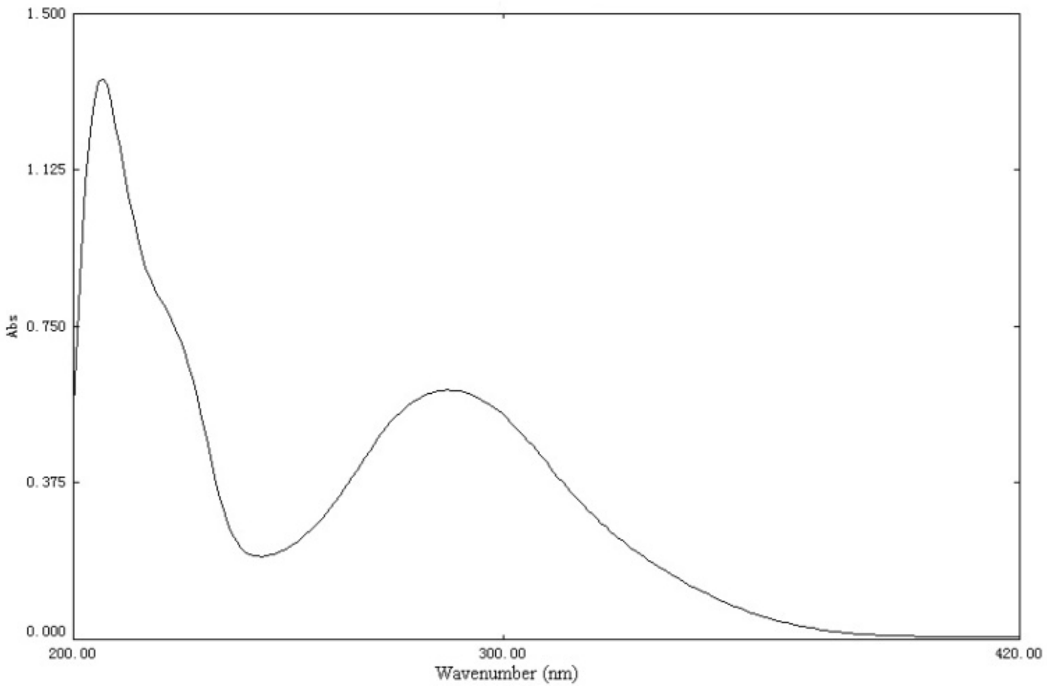

Figure S44. UV spectrum (MeOH) of raistrickione E (**5**)

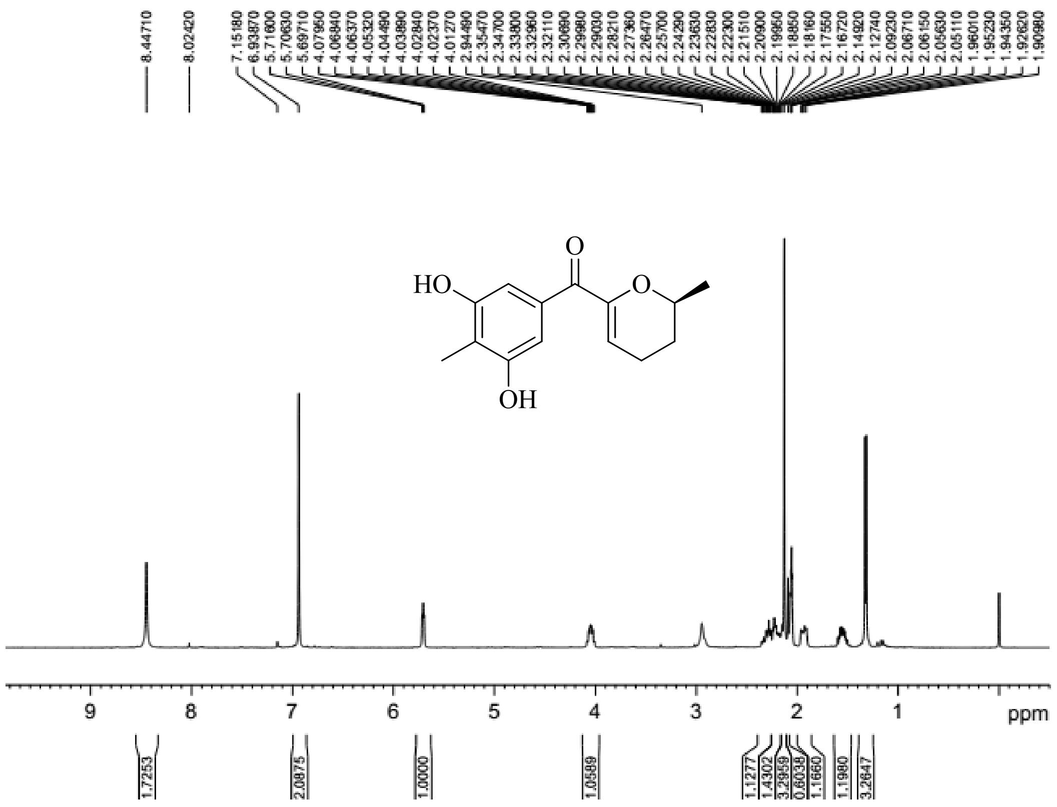

Figure S45. ^{1}H NMR spectrum (400 MHz acetone-d_6) of raistrickione E (**5**)

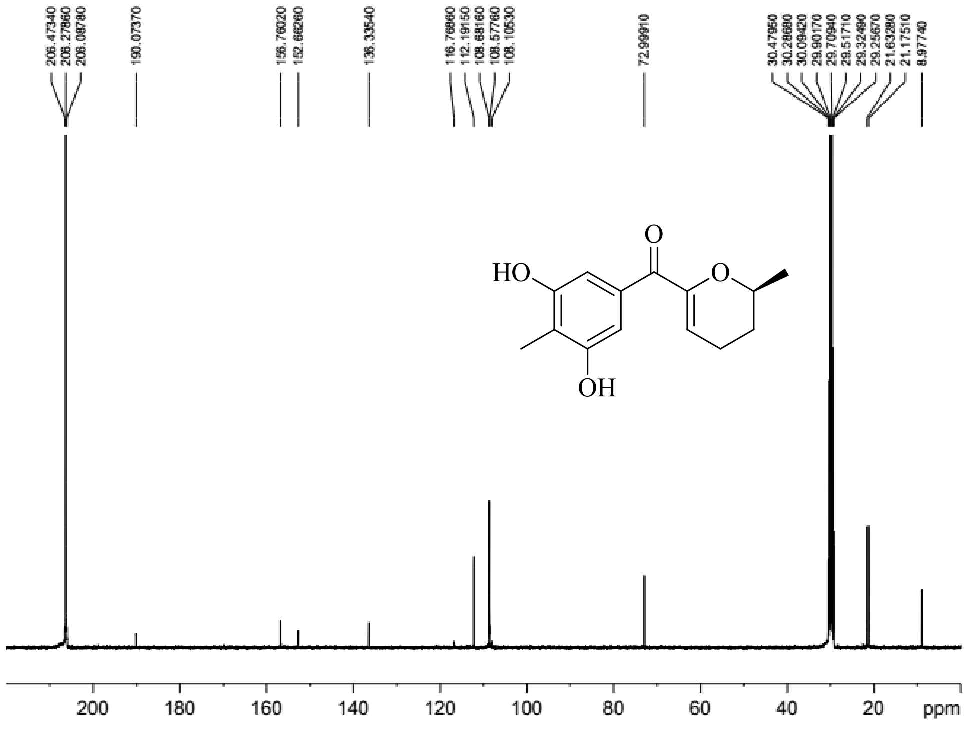

Figure S46. ^{13}C NMR spectrum (100 MHz acetone-d_6) of raistrickione E (**5**)

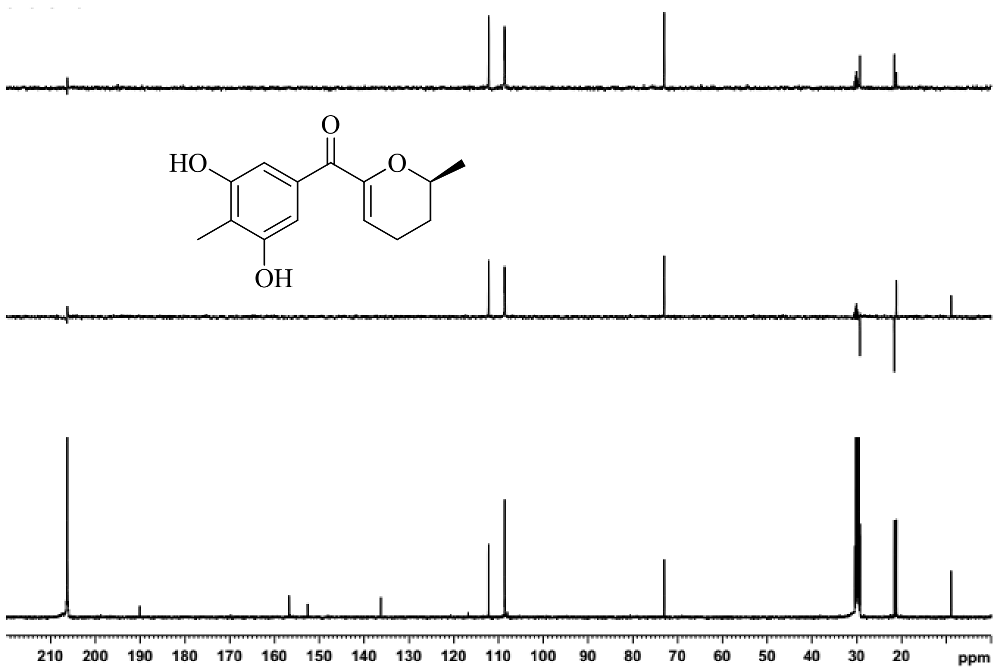

Figure S47. DEPT of raistrickione E (**5**)

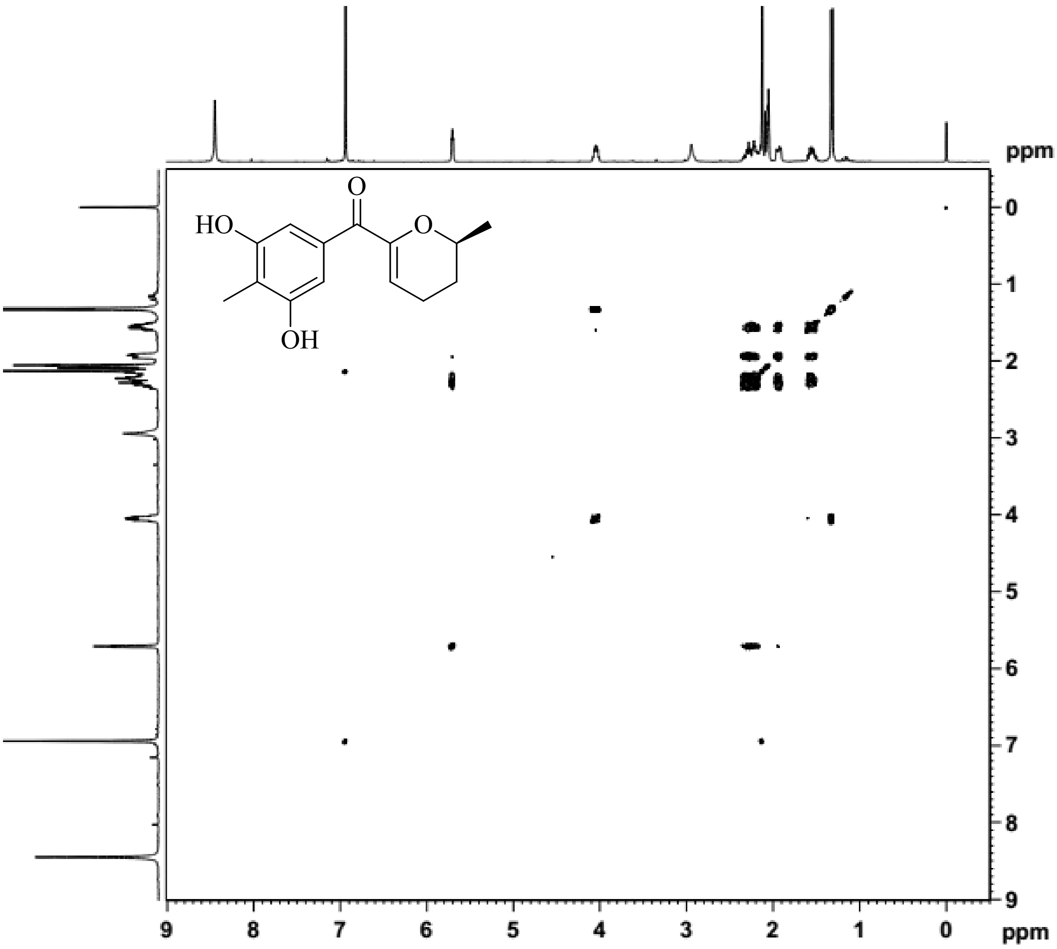

Figure S48. COSY of raistrickione E (**5**)

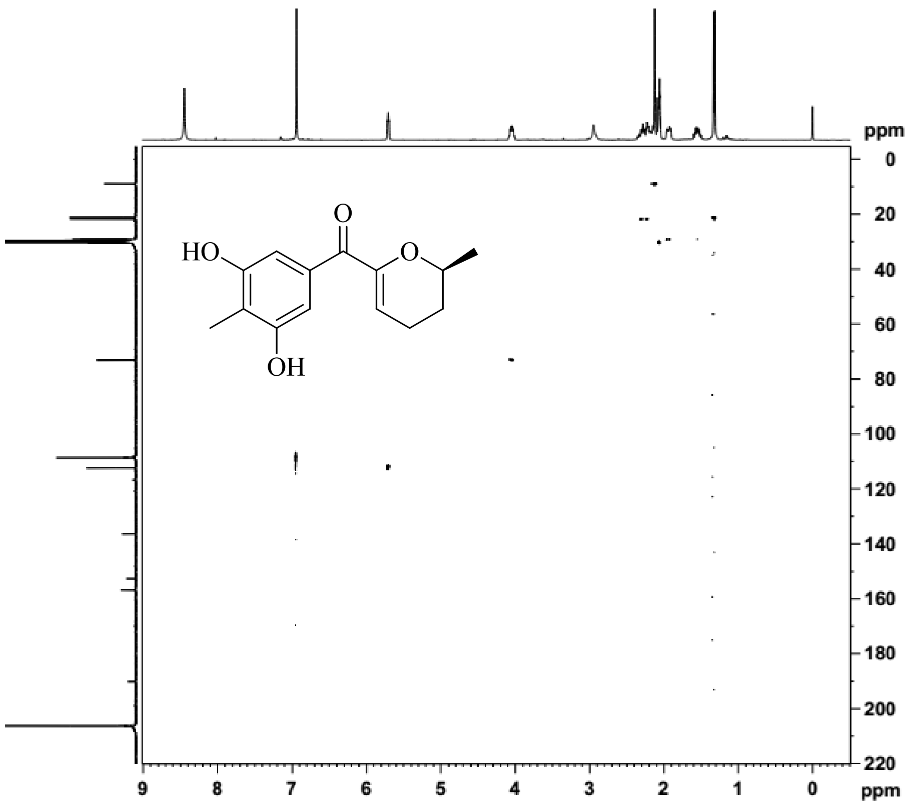

Figure S49. HSQC of raistrickione E (**5**)

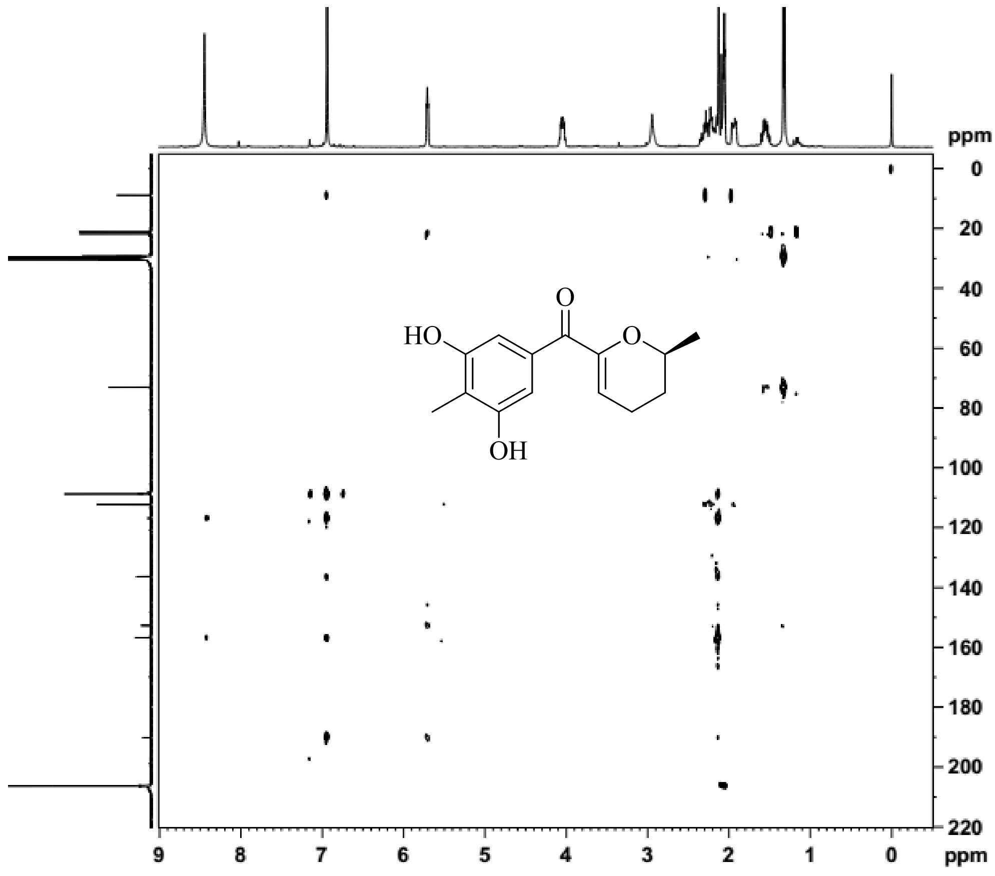

Figure S50. HMBC of raistrickione E (**5**)

Computational parts

Compound 1

1. Computational methods

1.1 ECD calculation

The initial three-dimensional structure of configuration a of compound **1** was obtained from the crystal structure while configuration b was built by mirror inverted of a. The theoretical calculations were carried out using Gaussian 09 [1]. Both structures were optimized at B3LYP/6-311G(d,p) in methanol using the IEFPCM model (**Table S1**). Vibrational frequency analysis confirmed the stable structures. Under the same condition, the ECD calculation was conducted using Time-dependent Density functional theory (TD-DFT). Rotatory strengths for a total of 30 excited states were calculated. The ECD spectrum was simulated in SpecDis [2] by overlapping Gaussian functions for each transition according to (3).

$$\Delta\varepsilon(E) = \frac{1}{2.297\times10^{-39}} \times \frac{1}{\sqrt{2\pi\sigma}} \sum_{i}^{A} \Delta E_i R_i e^{-\left(\frac{E-E_i}{2\sigma}\right)^2} \qquad \textbf{(1)}$$

where σ represents the width of the band at 1/*e* height, and ΔE_i and R_i are the excitation energies and rotatory strengths for transition *i*, respectively.

Parameters of σ and UV-shift for compound **1** were 0.26 eV and 0 nm, respectively.

1.2 References

1. M. J. Frisch, G. W. Trucks, H. B. Schlegel, G. E. Scuseria, M. A. Robb, J. R. Cheeseman, G. Scalmani, V. Barone, B. Mennucci, G. A. Petersson, H. Nakatsuji, M. Caricato, X. Li, H. P. Hratchian, A. F. Izmaylov, J. Bloino, G. Zheng, J. L. Sonnenberg, M. Hada, M. Ehara, K. Toyota, R. Fukuda, J. Hasegawa, M. Ishida, T. Nakajima, Y. Honda, O. Kitao, H. Nakai, T. Vreven, J. A. Montgomery, Jr., J. E. Peralta, F. Ogliaro, M. Bearpark, J. J. Heyd, E. Brothers, K. N. Kudin, V. N. Staroverov, R. Kobayashi, J. Normand, K. Raghavachari, A. Rendell, J. C. Burant, S. S. Iyengar, J. Tomasi, M. Cossi, N. Rega, J. M. Millam, M. Klene, J. E. Knox, J. B. Cross, V. Bakken, C. Adamo, J. Jaramillo, R. Gomperts, R. E. Stratmann, O. Yazyev, A. J. Austin, R. Cammi, C. Pomelli, J. W. Ochterski, R. L. Martin, K. Morokuma, V. G. Zakrzewski, G. A. Voth, P. Salvador, J. J. Dannenberg, S. Dapprich, A. D. Daniels, O. Farkas, J. B. Foresman, J. V. Ortiz, J. Cioslowski, and D. J. Fox. Gaussian 09 Revision D.01. Gaussian Inc. Wallingford CT 2009.
2. T. Bruhn, A. Schaumlöffel, Y. Hemberger and G. Bringmann, *Chirality*, 2013, **25**, 243-249.

2. Energies at B3LYP theory level

Structures for ECD calculations were optimized at B3LYP/6-311G(d,p) in methanol.

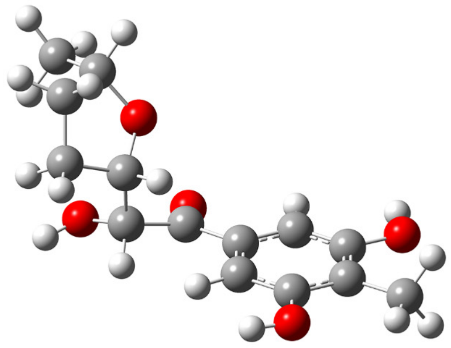

a

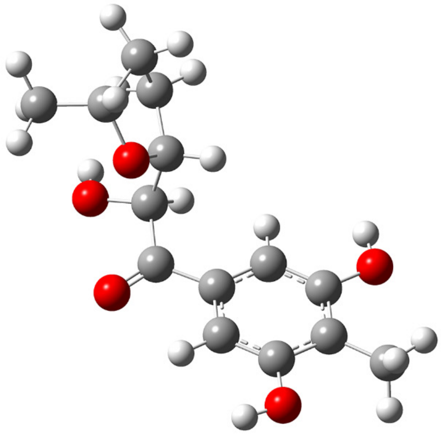

b

Both SCF Energy = -920.699470648 Hartree = -577747.64 kcal/mol.

3. Coordinates at B3LYP theory level

Table S1 Standard orientations of configurations a and b of compound **1** at B3LYP/6-311G(d,p) level in methanol.

Configuration a

Center Number	Atomic Number	Atomic Type	Coordinates (Angstroms) X	Y	Z
1	6	0	3.152089	-1.012046	-0.466066
2	6	0	3.597923	0.311792	-0.318257
3	6	0	2.716569	1.225981	0.273878
4	6	0	1.440348	0.843307	0.695680
5	1	0	0.805616	1.586053	1.162236
6	6	0	1.020362	-0.481095	0.535818
7	6	0	1.888723	-1.411707	-0.043375
8	1	0	1.564026	-2.439129	-0.156691
9	8	0	4.030318	-1.881033	-1.047090
10	1	0	3.627452	-2.755461	-1.104135
11	8	0	3.159189	2.510265	0.424253
12	1	0	2.468145	3.045544	0.830851
13	6	0	4.972531	0.707351	-0.784111
14	1	0	5.092124	0.508018	-1.853113
15	1	0	5.741054	0.124545	-0.267668
16	1	0	5.158028	1.763962	-0.604127
17	6	0	-0.319308	-0.976368	0.988462
18	6	0	-1.420268	0.044858	1.307181
19	1	0	-0.995125	0.841621	1.927871
20	6	0	-1.930510	0.699335	-0.000520
21	1	0	-1.097267	1.257209	-0.444637

22	6	0	-3.141328	1.624043	0.172932
23	1	0	-3.774315	1.284608	0.995575
24	1	0	-2.842723	2.653739	0.374440
25	6	0	-3.873295	1.456053	-1.165026
26	1	0	-3.424750	2.101365	-1.925085
27	1	0	-4.936655	1.693530	-1.103182
28	6	0	-3.619608	-0.020227	-1.509858
29	1	0	-3.512582	-0.161560	-2.589385
30	8	0	-2.331711	-0.324683	-0.921210
31	8	0	-0.543902	-2.167256	1.082833
32	6	0	-4.672521	-0.984484	-0.973460
33	1	0	-4.813591	-0.850617	0.101728
34	1	0	-4.357666	-2.016455	-1.146763
35	1	0	-5.630287	-0.827755	-1.478412
36	8	0	-2.445264	-0.634364	2.014324
37	1	0	-2.890763	0.006599	2.575950

Configuration b

Center Number	Atomic Number	Atomic Type	Coordinates (Angstroms) X	Y	Z
1	6	0	-3.152089	-1.012046	-0.466066
2	6	0	-3.597923	0.311792	-0.318257
3	6	0	-2.716569	1.225981	0.273878
4	6	0	-1.440348	0.843307	0.695680
5	1	0	-0.805616	1.586053	1.162236
6	6	0	-1.020362	-0.481095	0.535818
7	6	0	-1.888723	-1.411707	-0.043375
8	1	0	-1.564026	-2.439129	-0.156691
9	8	0	-4.030318	-1.881033	-1.047090
10	1	0	-3.627452	-2.755461	-1.104135
11	8	0	-3.159189	2.510265	0.424253
12	1	0	-2.468145	3.045544	0.830851
13	6	0	-4.972531	0.707351	-0.784111
14	1	0	-5.092124	0.508018	-1.853113
15	1	0	-5.741054	0.124545	-0.267668
16	1	0	-5.158028	1.763962	-0.604127
17	6	0	0.319308	-0.976368	0.988462
18	6	0	1.420268	0.044858	1.307181
19	1	0	0.995125	0.841621	1.927871
20	6	0	1.930510	0.699335	-0.000520
21	1	0	1.097267	1.257209	-0.444637
22	6	0	3.141328	1.624043	0.172932
23	1	0	3.774315	1.284608	0.995575
24	1	0	2.842723	2.653739	0.374440
25	6	0	3.873295	1.456053	-1.165026
26	1	0	3.424750	2.101365	-1.925085
27	1	0	4.936655	1.693530	-1.103182

28	6	0	3.619608	-0.020227	-1.509858
29	1	0	3.512582	-0.161560	-2.589385
30	8	0	2.331711	-0.324683	-0.921210
31	8	0	0.543902	-2.167256	1.082833
32	6	0	4.672521	-0.984484	-0.973460
33	1	0	4.813591	-0.850617	0.101728
34	1	0	4.357666	-2.016455	-1.146763
35	1	0	5.630287	-0.827755	-1.478412
36	8	0	2.445264	-0.634364	2.014324
37	1	0	2.890763	0.006599	2.575950

4. Experimental and calculated ECD spectra

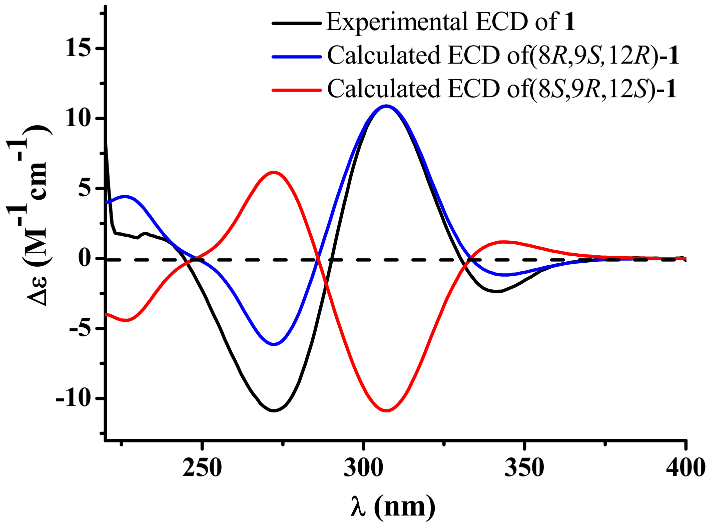

Figure S51 Calculated ECD spectra of compound **1** were compared with the experimental.

Compounds 4 and 5

1 Computational methods

1.1 Conformational analysis

Conformational analysis was initially performed using Confab[1] with systematic search at MMFF94 force field for undetermined relative configurations of compounds **4** and **5** (**Figure S52**). Room-temperature equilibrium populations were calculated according to Boltzmann distribution law (2). Energy calculated in MMFF94 is of inadequate accuracy and Boltzmann-populations concentrated on minority conformers. To avoid missing the authentic conformers, all output conformers were delivered to subsequent Quantum Mechanics (QM) calculations. The energies and populations of all conformers were provided in Table S2.

$$\frac{N_i}{N} = \frac{g_i e^{-\frac{E_i}{k_B T}}}{\sum g_i e^{-\frac{E_i}{k_B T}}} \qquad \textbf{(2)}$$

Where N_i is the number of conformer i with energy E_i and degeneracy g_i at temperature *T*, and k_B is Boltzmann constant.

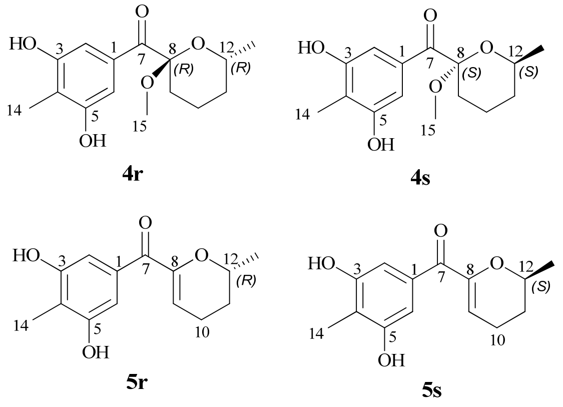

Figure S52. Relative configurations of compounds **4** and **5**

1.2 ECD calculation

The theoretical calculations were carried out using Gaussian 09.[2] At first, conformers were optimized at PM6 using semi-empirical theory method. The conformers with Boltzmann-population of over 1% were chosen for further optimization at B3LYP/6-311G(d,p) in methanol using the IEFPCM model (Table S4). Vibrational frequency analysis confirmed the stable structures. Under the same condition, the ECD calculation was conducted using Time-dependent Density functional theory (TD-DFT). Rotatory strengths for a total of 30 excited states were calculated. The ECD spectrum was simulated in SpecDis[3] by overlapping Gaussian functions for each transition according to (3).

$$\Delta\varepsilon(E) = \frac{1}{2.297\times10^{-39}} \times \frac{1}{\sqrt{2\pi\sigma}} \sum_i^A \Delta E_i R_i e^{-\left(\frac{E-E_i}{2\sigma}\right)^2} \qquad \textbf{(3)}$$

Where σ represents the width of the band at 1/*e* height, and ΔE_i and R_i are the excitation energies and rotatory strengths for transition *i*, respectively.

Parameters of σ and UV-shift for compounds **4** and **5** were list as follows.

Configuration	σ (eV)	UV-shift (nm)
4r	0.30	14
4s	0.30	12
5r	0.43	-7
5s	0.43	-7

1.3 References

1 N. M. O'Boyle, T. Vandermeersch, C. J. Flynn, A. R. Maguire and G. R. Hutchison, *J. Cheminform.*, 2011, **3**, 3-8.

2 M. J. Frisch, G. W. Trucks, H. B. Schlegel, G. E. Scuseria, M. A. Robb, J. R. Cheeseman, G. Scalmani, V. Barone, B. Mennucci, G. A. Petersson, H. Nakatsuji, M. Caricato, X. Li, H. P. Hratchian, A. F. Izmaylov,J. Bloino, G. Zheng, J. L. Sonnenberg, M. Hada, M. Ehara, K. Toyota, R. Fukuda, J. Hasegawa, M. Ishida, T. Nakajima, Y. Honda, O. Kitao, H. Nakai, T. Vreven, J. A. Montgomery, Jr., J. E. Peralta, F. Ogliaro, M. Bearpark, J. J. Heyd, E. Brothers, K. N. Kudin, V. N. Staroverov, R. Kobayashi, J. Normand, K. Raghavachari, A. Rendell, J. C. Burant, S. S. Iyengar, J. Tomasi, M. Cossi, N. Rega, J. M. Millam, M. Klene, J. E. Knox, J. B. Cross, V. Bakken, C. Adamo, J. Jaramillo, R. Gomperts, R. E. Stratmann, O. Yazyev, A. J. Austin, R. Cammi, C. Pomelli, J. W. Ochterski, R. L. Martin, K. Morokuma, V. G. Zakrzewski, G. A. Voth, P. Salvador, J. J. Dannenberg, S. Dapprich, A. D. Daniels, O. Farkas, J. B. Foresman, J. V. Ortiz, J. Cioslowski, and D. J. Fox. Gaussian 09, revision C.01. Gaussian, Inc.: Wallingford CT, 2010.

3 T. Bruhn, A. Schaumlöffel, Y. Hemberger and G. Bringmann, *Chirality*, 2013, **25**, 243-249.

2. Energies and Coordinates

2.1 Energies at MMFF94 force field

Systematic conformational search was performed by Confab program at MMFF94 force field. Conformers for each configuration were obtained with filtration by RMSD threshold of 0.5 Å.

Table S2 Energies of compound **4** and **5** at MMFF94 force field.

Compound	Conformer	Energy (kcal/mol)	Population (%)
4r	1	62.8417	50.74
	2	62.8912	46.67
	3	64.6395	2.44
	4	66.2971	0.15
	5	68.5542	0
	6	72.1436	0
	7	74.0531	0
	8	80.4148	0
	9	87.1893	0
	10	88.1756	0
	11	90.8256	0
	12	98.5946	0
	13	106.5830	0
4s	1	60.7387	98.98
	2	63.4457	1.02
	3	68.3388	0
	4	68.5738	0
	5	70.1233	0
	6	70.9081	0
	7	74.4711	0
	8	75.5671	0
	9	76.4813	0
	10	76.9425	0
	11	77.4402	0
	12	79.4152	0
5r	1	38.5954	99.78
	2	42.2604	0.2
	3	43.7405	0.02
	4	47.5363	0
	5	48.4513	0
5s	1	40.2693	99.68
	2	43.6886	0.31
	3	45.5821	0.01
	4	49.7547	0
	5	78.6294	0

2.2 Energies at B3LYP theory level

Structures for ECD calculations were optimized at B3LYP/6-311G(d,p) in methanol.

Table S3 Energies of compound **4** and **5** at B3LYP/6-311G(d,p) in methanol.

Configuration	Conformer	Structure	E (Hartree)	E (kcal/mol)	Population (%)
4r	2	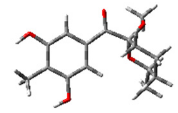	-960.01876206	-602420.86	34.69
4r	4	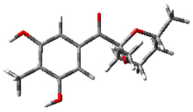	-960.01866575	-602420.80	31.33
4r	6	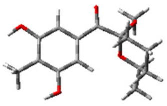	-960.01874228	-602420.85	33.98
4s	1	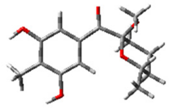	-960.01876206	-602420.86	34.69
4s	2	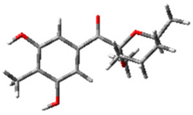	-960.01866575	-602420.80	31.33
4s	3	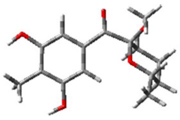	-960.01874229	-602420.85	33.98
5r	2	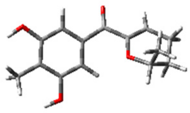	-844.23968902	-529768.40	8.07
5r	3	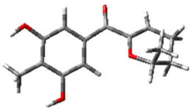	-844.23963208	-529768.36	7.60

5r	6	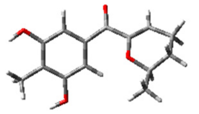	-844.24190460	-529769.79	84.33
		e			
5s	2	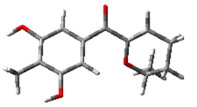	-844.23968902	-529768.40	8.07
5s	3	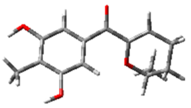	-844.23963208	-529768.36	7.60
5s	6	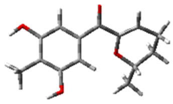	-844.24190460	-529769.79	84.33

2.3 Coordinates at B3LYP theory level

Table S4 Standard orientations of configurations of compounds **4** and **5** at B3LYP/6-311G(d,p) level in methanol.

Conformer 4r-2

Center Number	Atomic Number	Atomic Type	Coordinates (Angstroms) X	Y	Z
1	6	0	3.357080	-0.871243	-0.038734
2	6	0	3.499686	0.528894	-0.002378
3	6	0	2.321178	1.292185	0.024970
4	6	0	1.059474	0.699117	0.058316
5	1	0	0.189746	1.335189	0.063034
6	6	0	0.945698	-0.693990	0.051251
7	6	0	2.106577	-1.475187	0.000337
8	1	0	2.034518	-2.553845	-0.010915
9	6	0	-1.959205	-0.290151	1.686003
10	1	0	-2.051296	-1.194047	2.291986
11	1	0	-1.073272	0.251132	2.029014
12	6	0	-1.739228	-0.717055	0.221672
13	6	0	-0.354652	-1.440216	0.118730
14	6	0	4.861577	1.177611	-0.011688
15	1	0	5.604785	0.567595	0.508929
16	1	0	4.861414	2.135677	0.514067
17	1	0	5.229332	1.355945	-1.027765
18	8	0	4.434560	-1.710180	-0.096106
19	1	0	5.248623	-1.207879	-0.208322
20	8	0	2.337510	2.659684	0.031107
21	1	0	3.234209	2.987911	-0.095235
22	8	0	-0.357702	-2.655870	0.176161
23	6	0	-3.199484	0.600740	1.816482
24	1	0	-4.097462	0.005119	1.629461
25	1	0	-3.271657	0.982263	2.838575
26	6	0	-3.129786	1.758168	0.813088
27	1	0	-2.305623	2.433223	1.073387
28	1	0	-4.051972	2.346671	0.838734
29	6	0	-2.915058	1.235163	-0.605941
30	1	0	-3.779733	0.625798	-0.893696
31	8	0	-1.734493	0.387046	-0.664296
32	6	0	-2.699633	2.329472	-1.637231
33	1	0	-1.841247	2.951467	-1.369550
34	1	0	-3.584304	2.968286	-1.696215
35	1	0	-2.520607	1.897350	-2.624328
36	8	0	-2.789368	-1.610095	-0.075595
37	6	0	-2.861965	-2.094177	-1.420774
38	1	0	-2.926092	-1.272621	-2.138380
39	1	0	-3.772352	-2.690475	-1.473354
40	1	0	-2.004623	-2.725416	-1.664213

	Conformer 4r-4				
Center	Atomic	Atomic	Coordinates (Angstroms)		
Number	Number	Type	X	Y	Z
1	6	0	-3.378298	1.123385	-0.235510
2	6	0	-3.778380	-0.220062	-0.106967
3	6	0	-2.771394	-1.169050	0.133846
4	6	0	-1.423703	-0.815761	0.189942
5	1	0	-0.695388	-1.587130	0.387550
6	6	0	-1.050121	0.522341	0.028916
7	6	0	-2.039144	1.490982	-0.174443
8	1	0	-1.765091	2.531077	-0.284933
9	6	0	1.593168	-0.692862	-1.358500
10	1	0	0.718864	-1.334186	-1.472639
11	1	0	1.526072	0.093361	-2.117751
12	6	0	1.556701	-0.006350	0.018597
13	6	0	0.369054	1.013670	0.074335
14	6	0	-5.228826	-0.622962	-0.205897
15	1	0	-5.787451	0.028816	-0.882800
16	1	0	-5.732977	-0.603210	0.766210
17	1	0	-5.343017	-1.628079	-0.620088
18	8	0	-4.275240	2.133263	-0.441675
19	1	0	-5.177779	1.801238	-0.386168
20	8	0	-3.048963	-2.495516	0.313905
21	1	0	-4.000143	-2.637777	0.367322
22	8	0	0.606242	2.204639	0.092811
23	6	0	2.891256	-1.486014	-1.548710
24	1	0	2.892143	-2.350836	-0.878282
25	1	0	2.935762	-1.872187	-2.570648
26	6	0	4.104343	-0.599765	-1.252280
27	1	0	4.182483	0.193943	-2.005252
28	1	0	5.030086	-1.181821	-1.293279
29	6	0	3.975312	0.040973	0.127789
30	1	0	3.986890	-0.748822	0.889083
31	8	0	2.719157	0.757927	0.239051
32	6	0	5.063040	1.056935	0.432547
33	1	0	5.046894	1.865638	-0.303097
34	1	0	6.045460	0.579199	0.402579
35	1	0	4.919820	1.489529	1.425305
36	8	0	1.421462	-1.031325	0.993444
37	6	0	1.396319	-0.587477	2.354300
38	1	0	2.288838	-0.008205	2.599610
39	1	0	1.364614	-1.487518	2.966567
40	1	0	0.506691	0.017179	2.562646

	Conformer 4r-6				
Center	Atomic	Atomic	Coordinates (Angstroms)		
Number	Number	Type	X	Y	Z

1	6	0	3.358232	-0.872560	-0.017779
2	6	0	3.498466	0.528160	-0.031097
3	6	0	2.320694	1.290331	0.034447
4	6	0	1.058915	0.697486	0.060274
5	1	0	0.189345	1.333622	0.075401
6	6	0	0.945463	-0.695634	0.044625
7	6	0	2.107288	-1.476436	0.012594
8	1	0	2.035953	-2.555168	0.019829
9	6	0	-1.956789	-0.304001	1.681906
10	1	0	-2.049136	-1.212687	2.280643
11	1	0	-1.069774	0.233443	2.028094
12	6	0	-1.739296	-0.719168	0.213755
13	6	0	-0.355216	-1.442232	0.103008
14	6	0	4.855467	1.183945	-0.096945
15	1	0	5.578393	0.571645	-0.642103
16	1	0	5.269678	1.381968	0.897399
17	1	0	4.821576	2.133223	-0.638215
18	8	0	4.436303	-1.712292	-0.046813
19	1	0	5.255475	-1.213411	0.040522
20	8	0	2.338661	2.657509	0.057202
21	1	0	3.243429	2.982747	0.115466
22	8	0	-0.358908	-2.658439	0.146903
23	6	0	-3.195774	0.587391	1.821479
24	1	0	-4.094776	-0.005558	1.630895
25	1	0	-3.265933	0.960568	2.846790
26	6	0	-3.125969	1.752871	0.827476
27	1	0	-2.300498	2.424685	1.091987
28	1	0	-4.047295	2.342414	0.859373
29	6	0	-2.914163	1.241112	-0.596031
30	1	0	-3.780147	0.635237	-0.887283
31	8	0	-1.734764	0.392069	-0.663182
32	6	0	-2.699123	2.343414	-1.618834
33	1	0	-1.839569	2.962267	-1.347622
34	1	0	-3.583130	2.983735	-1.671206
35	1	0	-2.522232	1.919025	-2.609657
36	8	0	-2.790527	-1.608870	-0.089182
37	6	0	-2.866329	-2.080987	-1.438471
38	1	0	-2.930530	-1.253009	-2.148636
39	1	0	-3.777758	-2.675379	-1.494591
40	1	0	-2.010392	-2.711335	-1.688946

Conformer 4s-1

Center	Atomic	Atomic	Coordinates (Angstroms)		
Number	Number	Type	X	Y	Z
1	6	0	-2.321178	1.292185	0.024970
2	6	0	-3.499686	0.528894	-0.002378
3	6	0	-3.357080	-0.871243	-0.038734

4	6	0	-2.106577	-1.475187	0.000337
5	1	0	-2.034518	-2.553845	-0.010915
6	6	0	-0.945698	-0.693990	0.051251
7	6	0	-1.059474	0.699117	0.058316
8	1	0	-0.189746	1.335189	0.063034
9	6	0	1.959205	-0.290151	1.686003
10	1	0	1.073272	0.251132	2.029014
11	1	0	2.051296	-1.194046	2.291986
12	6	0	1.739228	-0.717055	0.221672
13	6	0	0.354652	-1.440216	0.118730
14	6	0	-4.861577	1.177611	-0.011688
15	1	0	-5.229333	1.355943	-1.027765
16	1	0	-4.861414	2.135678	0.514066
17	1	0	-5.604784	0.567595	0.508931
18	8	0	-2.337510	2.659684	0.031107
19	1	0	-3.234209	2.987911	-0.095235
20	8	0	-4.434560	-1.710180	-0.096106
21	1	0	-5.248623	-1.207879	-0.208322
22	8	0	0.357702	-2.655870	0.176161
23	6	0	3.199484	0.600740	1.816482
24	1	0	3.271657	0.982263	2.838575
25	1	0	4.097462	0.005120	1.629461
26	6	0	3.129786	1.758168	0.813088
27	1	0	4.051972	2.346671	0.838733
28	1	0	2.305623	2.433223	1.073387
29	6	0	2.915058	1.235162	-0.605941
30	1	0	3.779733	0.625798	-0.893696
31	8	0	1.734493	0.387046	-0.664296
32	6	0	2.699633	2.329472	-1.637231
33	1	0	1.841247	2.951467	-1.369551
34	1	0	2.520607	1.897349	-2.624328
35	1	0	3.584304	2.968286	-1.696215
36	8	0	2.789368	-1.610095	-0.075595
37	6	0	2.861965	-2.094177	-1.420774
38	1	0	2.004623	-2.725416	-1.664212
39	1	0	3.772353	-2.690475	-1.473354
40	1	0	2.926091	-1.272621	-2.138380

Conformer 4s-2

Center	Atomic	Atomic	Coordinates (Angstroms)		
Number	Number	Type	X	Y	Z
1	6	0	2.771394	-1.169050	0.133846
2	6	0	3.778380	-0.220062	-0.106967
3	6	0	3.378298	1.123385	-0.235510
4	6	0	2.039144	1.490982	-0.174443
5	1	0	1.765091	2.531077	-0.284933
6	6	0	1.050121	0.522341	0.028916

7	6	0	1.423703	-0.815761	0.189942
8	1	0	0.695388	-1.587130	0.387550
9	6	0	-1.593168	-0.692862	-1.358500
10	1	0	-1.526072	0.093361	-2.117751
11	1	0	-0.718864	-1.334186	-1.472639
12	6	0	-1.556701	-0.006350	0.018597
13	6	0	-0.369054	1.013670	0.074335
14	6	0	5.228826	-0.622963	-0.205896
15	1	0	5.343017	-1.628079	-0.620090
16	1	0	5.732976	-0.603213	0.766211
17	1	0	5.787452	0.028817	-0.882798
18	8	0	3.048963	-2.495515	0.313905
19	1	0	4.000143	-2.637777	0.367321
20	8	0	4.275240	2.133263	-0.441675
21	1	0	5.177779	1.801239	-0.386168
22	8	0	-0.606242	2.204639	0.092811
23	6	0	-2.891256	-1.486014	-1.548710
24	1	0	-2.935762	-1.872187	-2.570648
25	1	0	-2.892143	-2.350837	-0.878282
26	6	0	-4.104343	-0.599765	-1.252280
27	1	0	-5.030086	-1.181821	-1.293279
28	1	0	-4.182483	0.193943	-2.005252
29	6	0	-3.975312	0.040973	0.127789
30	1	0	-3.986890	-0.748822	0.889083
31	8	0	-2.719157	0.757926	0.239051
32	6	0	-5.063040	1.056935	0.432547
33	1	0	-5.046893	1.865638	-0.303097
34	1	0	-4.919820	1.489529	1.425305
35	1	0	-6.045460	0.579199	0.402579
36	8	0	-1.421462	-1.031325	0.993444
37	6	0	-1.396319	-0.587477	2.354300
38	1	0	-0.506692	0.017181	2.562645
39	1	0	-1.364611	-1.487518	2.966567
40	1	0	-2.288840	-0.008208	2.599611

Conformer 4s-3

Center	Atomic	Atomic	Coordinates (Angstroms)		
Number	Number	Type	X	Y	Z
1	6	0	-2.320700	1.290322	0.034379
2	6	0	-3.498476	0.528149	-0.031100
3	6	0	-3.358241	-0.872569	-0.017744
4	6	0	-2.107293	-1.476441	0.012605
5	1	0	-2.035959	-2.555173	0.019860
6	6	0	-0.945467	-0.695641	0.044582
7	6	0	-1.058921	0.697480	0.060193
8	1	0	-0.189351	1.333620	0.075270
9	6	0	1.956710	-0.304000	1.681916

10	1	0	1.069675	0.233438	2.028062
11	1	0	2.049037	-1.212681	2.280664
12	6	0	1.739294	-0.719173	0.213759
13	6	0	0.355217	-1.442243	0.102924
14	6	0	-4.855469	1.183955	-0.096913
15	1	0	-4.821606	2.133136	-0.638358
16	1	0	-5.269559	1.382165	0.897443
17	1	0	-5.578482	0.571588	-0.641877
18	8	0	-2.338672	2.657501	0.057084
19	1	0	-3.243442	2.982742	0.115303
20	8	0	-4.436308	-1.712308	-0.046706
21	1	0	-5.255484	-1.213421	0.040558
22	8	0	0.358914	-2.658451	0.146712
23	6	0	3.195682	0.587401	1.821547
24	1	0	3.265792	0.960579	2.846860
25	1	0	4.094698	-0.005540	1.631003
26	6	0	3.125914	1.752882	0.827542
27	1	0	4.047232	2.342434	0.859487
28	1	0	2.300423	2.424687	1.092014
29	6	0	2.914183	1.241121	-0.595974
30	1	0	3.780188	0.635254	-0.887184
31	8	0	1.734796	0.392060	-0.663184
32	6	0	2.699179	2.343416	-1.618792
33	1	0	1.839611	2.962265	-1.347619
34	1	0	2.522331	1.919018	-2.609619
35	1	0	3.583185	2.983742	-1.671132
36	8	0	2.790539	-1.608871	-0.089123
37	6	0	2.866457	-2.080947	-1.438421
38	1	0	2.010493	-2.711208	-1.689018
39	1	0	3.777841	-2.675417	-1.494461
40	1	0	2.930812	-1.252946	-2.148544

Conformer 5r-2

Center Number	Atomic Number	Atomic Type	Coordinates (Angstroms) X	Y	Z
1	6	0	-3.093121	0.864106	0.340404
2	6	0	-3.310868	-0.486113	0.007571
3	6	0	-2.205439	-1.221120	-0.452163
4	6	0	-0.928341	-0.665072	-0.515170
5	1	0	-0.110094	-1.284200	-0.849674
6	6	0	-0.736723	0.670607	-0.154765
7	6	0	-1.829766	1.438537	0.258295
8	1	0	-1.700044	2.482575	0.508706
9	6	0	2.997358	1.151236	-0.749882
10	1	0	2.932198	2.152235	-1.155424
11	6	0	1.874918	0.613669	-0.244768
12	6	0	0.580456	1.374565	-0.287439

13	6	0	-4.679755	-1.108947	0.131605
14	1	0	-5.231740	-0.700665	0.982826
15	1	0	-5.289795	-0.958161	-0.765476
16	1	0	-4.623621	-2.184286	0.317397
17	8	0	-4.104266	1.677710	0.770580
18	1	0	-4.953096	1.227881	0.700072
19	8	0	-2.309404	-2.528444	-0.841436
20	1	0	-3.234032	-2.794736	-0.883057
21	8	0	0.607122	2.588846	-0.440558
22	6	0	4.298694	0.408893	-0.776909
23	1	0	4.990401	0.840824	-0.041765
24	1	0	4.784313	0.541434	-1.749019
25	6	0	4.064318	-1.081441	-0.498467
26	1	0	3.659459	-1.560998	-1.394584
27	1	0	4.999314	-1.590625	-0.252920
28	6	0	3.066323	-1.276924	0.644722
29	1	0	2.793733	-2.331278	0.714104
30	8	0	1.801367	-0.624860	0.331213
31	6	0	3.562204	-0.797372	2.006191
32	1	0	3.801666	0.268059	1.999448
33	1	0	2.798753	-0.972297	2.766998
34	1	0	4.462486	-1.349517	2.288634
	Conformer 5r-3				
Center Number	Atomic Number	Atomic Type	Coordinates (Angstroms)		
			X	Y	Z
1	6	0	-3.091846	0.862218	0.353521
2	6	0	-3.319674	-0.476022	-0.018424
3	6	0	-2.205997	-1.227963	-0.428496
4	6	0	-0.928878	-0.672839	-0.497487
5	1	0	-0.108310	-1.299044	-0.812258
6	6	0	-0.737936	0.667634	-0.154576
7	6	0	-1.827946	1.435296	0.266459
8	1	0	-1.694323	2.475744	0.529493
9	6	0	2.993765	1.146601	-0.765430
10	1	0	2.925267	2.143963	-1.179281
11	6	0	1.874580	0.611880	-0.250160
12	6	0	0.578972	1.371059	-0.293249
13	6	0	-4.700823	-1.081358	0.037227
14	1	0	-4.921638	-1.526872	1.013077
15	1	0	-5.477531	-0.341587	-0.174230
16	1	0	-4.835429	-1.858237	-0.720026
17	8	0	-4.097634	1.674581	0.798171
18	1	0	-4.910724	1.171760	0.915278
19	8	0	-2.308090	-2.542653	-0.791639
20	1	0	-3.198972	-2.869600	-0.626526
21	8	0	0.604119	2.584461	-0.453153

22	6	0	4.295929	0.405648	-0.792638
23	1	0	4.990421	0.843981	-0.063917
24	1	0	4.777001	0.531166	-1.767921
25	6	0	4.064473	-1.082719	-0.501596
26	1	0	3.655314	-1.569453	-1.391879
27	1	0	5.001247	-1.589169	-0.257158
28	6	0	3.072858	-1.270486	0.648352
29	1	0	2.801400	-2.324496	0.726870
30	8	0	1.805530	-0.621645	0.336936
31	6	0	3.575403	-0.780656	2.003644
32	1	0	3.813939	0.284898	1.988035
33	1	0	2.816074	-0.950706	2.769673
34	1	0	4.477590	-1.330048	2.285386

Conformer 5r-6

Center Number	Atomic Number	Atomic Type	Coordinates (Angstroms) X	Y	Z
1	6	0	3.076671	-0.835323	0.403546
2	6	0	3.242997	0.490730	-0.038085
3	6	0	2.110789	1.144876	-0.552029
4	6	0	0.855150	0.539384	-0.563741
5	1	0	0.015716	1.098025	-0.948807
6	6	0	0.714387	-0.769197	-0.096496
7	6	0	1.835115	-1.460948	0.371981
8	1	0	1.744526	-2.485810	0.705440
9	6	0	-3.004478	-1.389570	-0.614480
10	1	0	-2.903808	-2.375357	-1.049233
11	6	0	-1.893511	-0.807189	-0.135608
12	6	0	-0.576522	-1.526174	-0.172255
13	6	0	4.585217	1.177162	0.025058
14	1	0	5.179036	0.829719	0.874604
15	1	0	5.178104	1.018533	-0.882109
16	1	0	4.482002	2.255515	0.171116
17	8	0	4.116818	-1.574025	0.894698
18	1	0	4.948789	-1.101667	0.782692
19	8	0	2.167122	2.418765	-1.047076
20	1	0	3.081063	2.716043	-1.111587
21	8	0	-0.562762	-2.746484	-0.267928
22	6	0	-4.352767	-0.738687	-0.535638
23	1	0	-5.091062	-1.466316	-0.181646
24	1	0	-4.685937	-0.444697	-1.539228
25	6	0	-4.309880	0.478081	0.397705
26	1	0	-5.173573	1.127137	0.234471
27	1	0	-4.337377	0.152101	1.442875
28	6	0	-3.027119	1.276974	0.174091
29	1	0	-2.974762	1.584454	-0.878672
30	8	0	-1.863179	0.441107	0.422014

31	6	0	-2.884825	2.486852	1.078158
32	1	0	-2.927732	2.187365	2.128579
33	1	0	-1.933306	2.992011	0.899277
34	1	0	-3.693000	3.196223	0.885313

Conformer 5s-2

Center	Atomic	Atomic	Coordinates (Angstroms)		
Number	Number	Type	X	Y	Z
1	6	0	3.093121	0.864106	0.340404
2	6	0	3.310868	-0.486113	0.007571
3	6	0	2.205439	-1.221120	-0.452163
4	6	0	0.928341	-0.665072	-0.515170
5	1	0	0.110094	-1.284200	-0.849674
6	6	0	0.736723	0.670607	-0.154765
7	6	0	1.829766	1.438537	0.258295
8	1	0	1.700044	2.482575	0.508706
9	6	0	-2.997358	1.151236	-0.749882
10	1	0	-2.932198	2.152235	-1.155424
11	6	0	-1.874918	0.613669	-0.244768
12	6	0	-0.580456	1.374565	-0.287439
13	6	0	4.679755	-1.108947	0.131605
14	1	0	5.231740	-0.700665	0.982826
15	1	0	5.289795	-0.958161	-0.765476
16	1	0	4.623621	-2.184286	0.317397
17	8	0	4.104266	1.677710	0.770580
18	1	0	4.953096	1.227881	0.700072
19	8	0	2.309404	-2.528444	-0.841436
20	1	0	3.234032	-2.794736	-0.883057
21	8	0	-0.607122	2.588846	-0.440558
22	6	0	-4.298694	0.408893	-0.776909
23	1	0	-4.990401	0.840824	-0.041765
24	1	0	-4.784313	0.541434	-1.749019
25	6	0	-4.064318	-1.081441	-0.498467
26	1	0	-3.659459	-1.560998	-1.394584
27	1	0	-4.999314	-1.590625	-0.252920
28	6	0	-3.066323	-1.276924	0.644722
29	1	0	-2.793733	-2.331278	0.714104
30	8	0	-1.801367	-0.624860	0.331213
31	6	0	-3.562204	-0.797372	2.006191
32	1	0	-3.801666	0.268059	1.999448
33	1	0	-2.798753	-0.972297	2.766998
34	1	0	-4.462486	-1.349517	2.288634

Conformer 5s-3

Center	Atomic	Atomic	Coordinates (Angstroms)		
Number	Number	Type	X	Y	Z
1	6	0	3.091846	0.862218	0.353521
2	6	0	3.319674	-0.476022	-0.018424

3	6	0	2.205997	-1.227963	-0.428496
4	6	0	0.928878	-0.672839	-0.497487
5	1	0	0.108310	-1.299044	-0.812258
6	6	0	0.737936	0.667634	-0.154576
7	6	0	1.827946	1.435296	0.266459
8	1	0	1.694323	2.475744	0.529493
9	6	0	-2.993765	1.146601	-0.765430
10	1	0	-2.925267	2.143963	-1.179281
11	6	0	-1.874580	0.611880	-0.250160
12	6	0	-0.578972	1.371059	-0.293249
13	6	0	4.700823	-1.081358	0.037227
14	1	0	4.921638	-1.526872	1.013077
15	1	0	5.477531	-0.341587	-0.174230
16	1	0	4.835429	-1.858237	-0.720026
17	8	0	4.097634	1.674581	0.798171
18	1	0	4.910724	1.171760	0.915278
19	8	0	2.308090	-2.542653	-0.791639
20	1	0	3.198972	-2.869600	-0.626526
21	8	0	-0.604119	2.584461	-0.453153
22	6	0	-4.295929	0.405648	-0.792638
23	1	0	-4.990421	0.843981	-0.063917
24	1	0	-4.777001	0.531166	-1.767921
25	6	0	-4.064473	-1.082719	-0.501596
26	1	0	-3.655314	-1.569453	-1.391879
27	1	0	-5.001247	-1.589169	-0.257158
28	6	0	-3.072858	-1.270486	0.648352
29	1	0	-2.801400	-2.324496	0.726870
30	8	0	-1.805530	-0.621645	0.336936
31	6	0	-3.575403	-0.780656	2.003644
32	1	0	-3.813939	0.284898	1.988035
33	1	0	-2.816074	-0.950706	2.769673
34	1	0	-4.477590	-1.330048	2.285386

Conformer 5s-6

Center	Atomic	Atomic	Coordinates (Angstroms)		
Number	Number	Type	X	Y	Z
1	6	0	-3.076671	-0.835323	0.403546
2	6	0	-3.242997	0.490730	-0.038085
3	6	0	-2.110789	1.144876	-0.552029
4	6	0	-0.855150	0.539384	-0.563741
5	1	0	-0.015716	1.098025	-0.948807
6	6	0	-0.714387	-0.769197	-0.096496
7	6	0	-1.835115	-1.460948	0.371981
8	1	0	-1.744526	-2.485810	0.705440
9	6	0	3.004478	-1.389570	-0.614480
10	1	0	2.903808	-2.375357	-1.049233
11	6	0	1.893511	-0.807189	-0.135608

12	6	0	0.576522	-1.526174	-0.172255
13	6	0	-4.585217	1.177162	0.025058
14	1	0	-5.179036	0.829719	0.874604
15	1	0	-5.178104	1.018533	-0.882109
16	1	0	-4.482002	2.255515	0.171116
17	8	0	-4.116818	-1.574025	0.894698
18	1	0	-4.948789	-1.101667	0.782692
19	8	0	-2.167122	2.418765	-1.047076
20	1	0	-3.081063	2.716043	-1.111587
21	8	0	0.562762	-2.746484	-0.267928
22	6	0	4.352767	-0.738687	-0.535638
23	1	0	5.091062	-1.466316	-0.181646
24	1	0	4.685937	-0.444697	-1.539228
25	6	0	4.309880	0.478081	0.397705
26	1	0	5.173573	1.127137	0.234471
27	1	0	4.337377	0.152101	1.442875
28	6	0	3.027119	1.276974	0.174091
29	1	0	2.974762	1.584454	-0.878672
30	8	0	1.863179	0.441107	0.422014
31	6	0	2.884825	2.486852	1.078158
32	1	0	2.927732	2.187365	2.128579
33	1	0	1.933306	2.992011	0.899277
34	1	0	3.693000	3.196223	0.885313
